# Circumdatin-Aspyrone Conjugates from the Coral-Associated *Aspergillus ochraceus* LCJ11-102

**DOI:** 10.3390/md17070400

**Published:** 2019-07-06

**Authors:** Yaqin Fan, Yalin Zhou, Yuqi Du, Yi Wang, Peng Fu, Weiming Zhu

**Affiliations:** 1 Key Laboratory of Marine Drugs, Ministry of Education of China, School of Medicine and Pharmacy, Ocean University of China, Qingdao 266003, China; 2 Open Studio for Druggability Research of Marine Natural Products, Laboratory for Marine Drugs and Bioproducts, Pilot National Laboratory for Marine Science and Technology (Qingdao), Qingdao 266003, China

**Keywords:** ochrazepines, conjugates, semisynthesis, cytotoxic activity, *Aspergillus ochraceus*

## Abstract

Ochrazepines A−D (**1**−**4**), four new conjugates dimerized from 2-hydroxycircumdatin C (**5**) and aspyrone (**6**) by a nucleophilic addition to epoxide, were isolated from the fermentation broth of the coral-associated *Aspergillus ochraceus* strain LCJ11-102. Their structures including absolute configurations were determined based on spectroscopic analysis and chemical methods. Compounds **1**−**4** were also obtained by the semisynthesis from a nucleophilic addition of 2-hydroxycircumdatin C (**5**) to aspyrone (**6**). New compound **1** exhibited cytotoxic activity against 10 human cancer cell lines while new compounds **2** and **4** selectively inhibited U251 (human glioblastoma cell line) and compound **3** was active against A673 (human rhabdomyoma cell line), U87 (human glioblastoma cell line), and Hep3B (human liver cancer cell line) with IC_50_ (half maximal inhibitory concentration) values of 2.5–11.3 μM among 26 tested human cancer cell lines.

## 1. Introduction

As more and more dimeric compounds were discovered from organisms, dimers have become an important and large part of the natural products (NPs) family [1]. Xanthone dimers [2] and bioflavonoids [3] are typical representatives of dimeric NPs. Some compounds possessing dimeric structures were found to have more significant biological activity than their corresponding monomers. Therefore, the NPs’ dimerization has been developed as an effective strategy for drug discovery [4]. Moreover, the hybrid compounds formed from two different types of NPs have also attracted a lot of attention. For example, citrifurans A−D were thought to be formed from azaphilone and furanone derivatives through a Michael addition [5]. Cnidimonins A−C were postulated to be formed through hybrid-dimerization patterns of coumarin with flavanol, benzofuran, and chromone, respectively [6]. The formation of some hybrid NPs depends on non-enzymatic steps. For example, discoipyrroles A−D from the marine bacterium *Bacillus hunanensis* [7], were found to be originated from 4-hydroxysattabacin, 4-hydroxybenzaldehyde, and 2-amino benzoic acid through non-enzymatic reactions [8]. Dibohemamines A−C, three bohemamine dimers from a marine-derived *Streptomyces* sp., were found to be dimerized from the monomeric bohemamines and formaldehyde by a non-enzymatic reaction [9]. The formation of hybrids can greatly expand the chemical space of NPs, which will provide more compounds for bioactivity studies.

**Figure 1:**
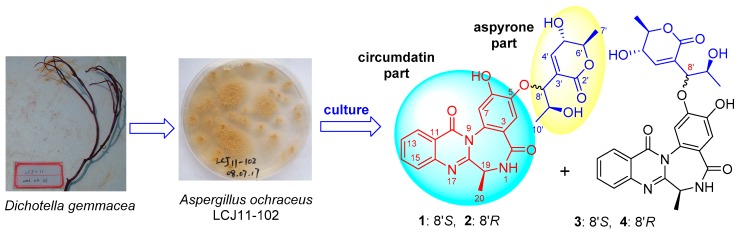
Structures of compounds **1**–**4**.

Coral-associated microorganisms are an important part of our research. From this type of organism, we have identified some bioactive metabolites with new structures, such as cottoquinazolines B−D [10], versicoloritides A−C and tetraorcinol A [11], and strepchloritides A and B [12]. As part of an ongoing search for bioactive NPs with novel structures from coral-associated microorganisms, we screened 78 fungal strains isolated from 12 coral samples. Among them, the *Aspergillus ochraceus* strain LCJ11-102, isolated from *Dichotella gemmacea*, showed a rich chemical diversity. Previously, we identified pyranone and furanone derivatives [13] and pyrazinone derivatives [14] from the fermentation broth of *A*. *ochraceus* LCJ11-102 under a nutrition-poor medium and high iodide salt, respectively. In a re-fermentation broth with large scale, a series of minor metabolites with UV absorptions at λ_max_ 230, 275, and 325 nm were observed, which are similar to those of circumdatins [15,16,17,18,19,20,21]. To the best of our knowledge, only 13 circumdatins have been isolated from *Aspergillus* sp. Some of them were found to have different biological activities. For example, circumdatins E and H showed inhibitory activity against the integrated electron transfer chain [18], and circumdatins C, G, and I exhibited potent ultraviolet-A (UV-A) protecting activity [20]. Four circumdatin analogues with molecular weight of 507 Da were observed in the LC-MS profile of the extract of the culture of LCJ11-102, larger than those of known circumdatins. These metabolites attracted our attention. Chemical investigation led to the identification of 2-hydroxycircumdatin C (**5**) [21] and aspyrone (**6**) [22], as well as their conjugates, ochrazepines A−D (**1**−**4**) (Figure 1), four new hybrid dimers.

## 2. Results and Discussion

**Figure 2:**
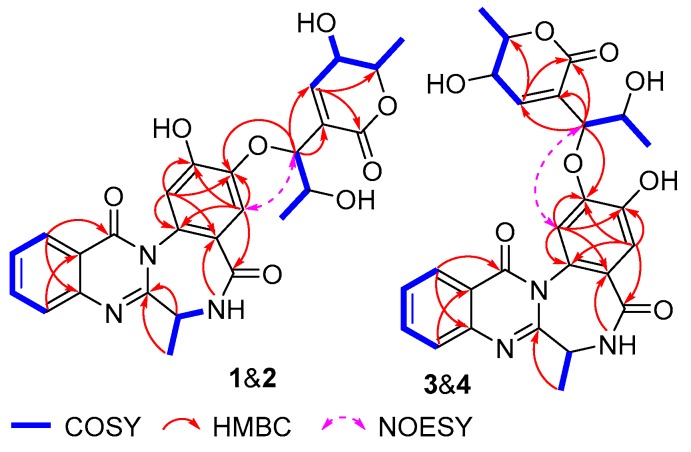
Key 2D NMR correlations for the structural assignment of compounds **1**–**4**.

Ochrazepine A (**1**) was obtained as a reddish-brown powder. Its molecular formula was determined as C_26_H_25_N_3_O_8_ based on the HRESIMS (high resolution electrospray ionization mass spectroscopy) peak at *m*/*z* 508.1718 [M + H]^+^ (Figure S4). The ^13^C NMR (nuclear magnetic resonance) spectrum (Figure S7) showed 26 signals which were classified by HSQC (heteronuclear single quantum correlation) (Figures S8 and S9) as 11 non-protonated carbons, seven sp^2^-methine carbons, five sp^3^-methine carbons including four oxygenated ones, and three methyl carbons (Table 1). Its ^1^H NMR spectrum (Figures S5 and S6) displayed the signals for one 1,2-disubstituted benzene ring (*δ*_H_ 8.17, dd, *J* = 7.9, 1.5; *δ*_H_ 7.58, td, *J* = 8.1, 1.2; *δ*_H_ 7.89, td, *J* = 8.4, 1.5 and *δ*_H_ 7.72, d, *J* = 8.2), two 1,4-phenyl hydrogen (*δ*_H_ 7.03 s; *δ*_H_ 6.99, s), one NH (*δ*_H_ 8.55, d, *J* =5.8), one sp^3^ methine (*δ*_H_ 4.32, m) and one methyl (*δ*_H_ 1.51, d, *J* = 6.7) (Table 1), which revealed the presence of a 2-hydroxycircumdatin C fragment [21]. This structural unit could be verified by the COSY (correlation spectroscopy) correlations of H-12/H-13/H-14/H-15 and HN-1/H-19/H-20 (Figures S10 and S11), and the key HMBC (heteronuclear multiple bond correlation) correlations (Figures S12‒S15) of HN-1 to C-3, H-4 to C-2/C-5/C-6/C-8, H-7 to C-3/C-5/C-6/C-8, H-12 to C-10/C-16, H-15 to C-11, as well as H-19 and H_3_-20 to C-18 (Figure 2). Careful analysis of the remaining ^1^H and ^13^C NMR data led to the identification of one ester carbonyl (*δ*_C_ 162.9), one trisubstituted ethenyl (*δ*_C_ 127.4_,_ *δ*_C/H_ 146.4/6.83), four oxygenated sp^3^ methines (*δ*_C/H_ 65.9/4.19, *δ*_C/H_, 79.1/4.27, *δ*_C/H_ 79.1/4.88, *δ*_C/H_ 68.1/3.89) and two methyls (*δ*_C/H_ 17.9/1.32_,_ *δ*_C/H_ 19.6/1.19) (Table 1), which allowed the construction of an α,β-unsaturated *δ*-lactone moiety. This unit was confirmed by the COSY correlations of H-4’/H-5’/5’-OH, and H-5’/H-6’/H_3_-7’ (Figures S10 and S11), along with the HMBC correlations of H-4’ to C-2’/C-6’ (Figures S12 and S15). In addition, the COSY correlations of H_3_-10’/H-9’/H-8’ (Figures S10 and S11), as well as the HMBC correlations of H-8’ to C-3’/4’ (Figures S13 and S14) displayed a similar correlative pattern to aspyrone (**6**) [22], except that the epoxy group was replaced by vicinal diol. The skeletal structure of **1** was further assigned by the analysis of key HMBC correlation of H-8’ to C-5 (Figure S14) and the NOE (nuclear Overhauser effect) correlation between H-8’ and H-4 (Figures S16 and S17).

Ochrazepine B (**2**) with the same molecular formula as **1** was assigned on the basis of the HRESIMS peak at *m*/*z* 508.1717 [M + H]^+^ (Figure S21), which indicated compound **2** is an isomer of ochrazepine A (**1**). Compound **2** also showed similar UV, IR (infrared), ^1^H (Figures S22 and S23) and ^13^C NMR (Figure S24) spectra to compound **1**. The most significant difference is the chemical shift of C-10’, that is *δ*_C_ 19.6 for **1** and *δ*_C_ 18.2 for **2** (Table 1), respectively. In addition, compound **2** showed almost the same 2D NMR correlations (Figures S25‒S31) with **1** (Figure 2), implying the same constitution. All these results indicated that compound **2** may be an 8’- or 9’-epimer of **1**.

Ochrazepines C (**3**) and D (**4**) also have the same molecular formulae based on their HRESIMS spectra at *m*/*z* 508.1721 (Figure S37) and 508.1711 [M + H]^+^ (Figure S51), respectively. Examination of their NMR data (Table 1, Figures S38‒S41 and S52‒S55) showed that they both have the same aspyrone and 2-hydroxycircumdatin C fragments, which could be confirmed by the 2D NMR correlations (Figure 2, Figures S42‒S46 and S56‒S60). The key HMBC correlations of H-8’ to C-6 were observed in the HMBC spectra of **3** (Figure S45) and **4** (Figure S59), different from those correlations of H-8’ to C-5 in **1** and **2**. Moreover, the NOE correlations of H-8’/H-4 in **1** and **2** were replaced by H-8’/H-7 in **3** (Figures S47 and S48) and **4** (Figures S61 and S62). These data showed that the aspyrone moiety of stereoisomers **3** and **4** was added to 6-OH of the 2-hydroxycircumdatin C fragment.

**Scheme 1:**
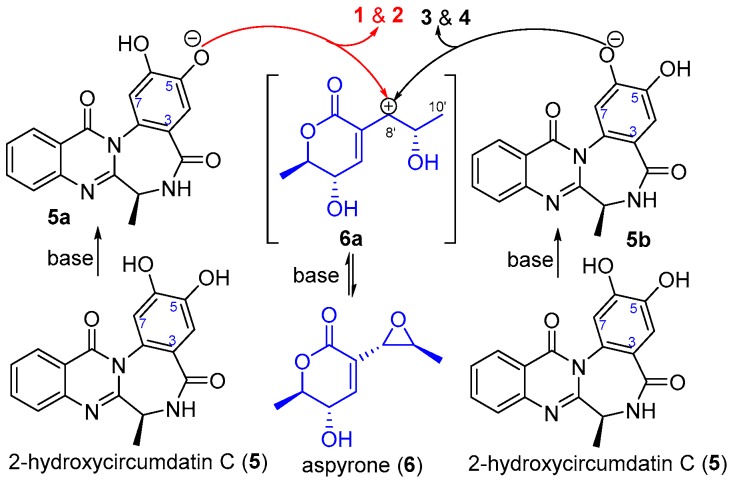
The postulated biosynthesis of compounds **1**–**4**.

**Scheme 2:**
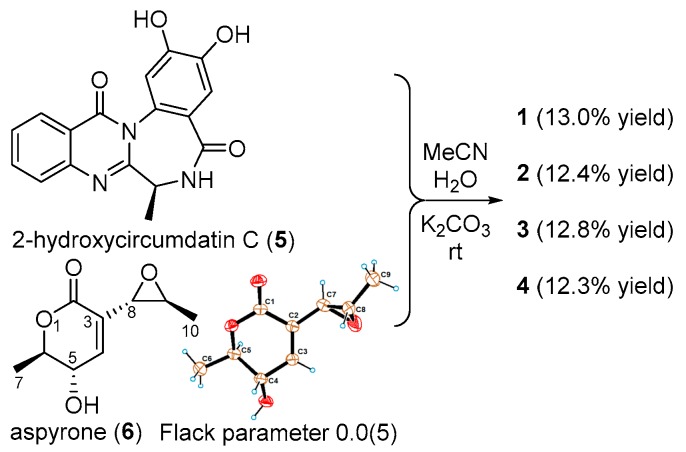
Semisynthesis of compounds **1**–**4**.

Analysis of the structures of ochrazepines A−D (**1**−**4**) and the compounds **5** and **6** isolated from this fungal strain allowed us to speculate that compounds **1**−**4** may be derived from 2-hydroxycircumdatin C (**5**) and aspyrone (**6**) by a non-enzymatic addition reaction (Scheme 1). To confirm this speculation and determine the absolute configurations of the new compounds **1**−**4**, the nucleophilic addition reactions of **6** with **5** were carried out in MeCN/H_2_O under basic (K_2_CO_3_), neutural and acidic (CF_3_CO_2_H) conditions, respectively. Ochrazepines A−D (**1**−**4**) were only isolated and identified from the reaction mixtures under basic condition (Scheme 2 and Figure S1) while the reaction did not take place under neutral condition (Figure S2) or compound **6** undergoes an epoxide ring-opening reaction with CF_3_CO_2_H in acidic condition (Figure S3). The results indicated that aspyrone (**6**) possibly underwent a S_N_1-like process to form the more stable allyl carbon positive ion (**6a**) under basic condition and then rapidly reacted with the oxygen anions of 5-OH (**5a**) and 6-OH (**5b**) of 2-hydroxycircumdatin C (**5**) to yield two pairs of epimers **1**/**2** and **3**/**4**, respectively (Scheme 1). Thus, compounds **1**−**4** have the same absolute configurations except C-8’ chiral center. Therefore, we unambiguously determine the absolute configurations of 2-hydroxycircumdatin C (**5**) as 19*S* (Figure S66) by Marfey’s method [23] and aspyrone (**6**) as (5*S*,6*R*,8*S*,9*S*) through single-crystal X-ray diffraction with Cu-Kα irradiation (CCDC No. 1876311) (Scheme 2), respectively.

**Figure 3:**
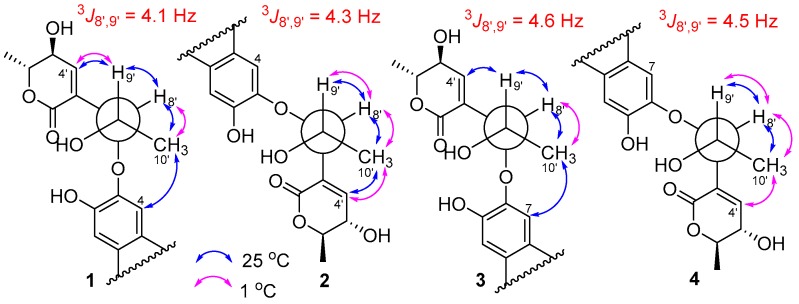
Analysis of coupling constants and NOE correlations for compounds **1**–**4**.

In order to establish the C-8’ configuration, we analyzed the NOESY (nuclear Overhauser enhancement spectroscopy) spectra of compounds **1**−**4** and the coupling constants between H-8’ and H-9’ (Figure 3). The *J*_8’, 9’_ values of **1**−**4** are all located at 4−5 Hz (Table 1). Both compounds **1** and **3** displayed key correlations of H-4’/H-9’ and H-4/H_3_-10’ (for **1**) (Figures S16 and S17) or H-7/H_3_-10’ (for **3**) (Figures S47 and S48) in NOESY spectra at 25 °C. The NOESY spectrum of **1** at 1 °C also showed the key correlations of H-4’/H-9’ (Figures S18‒S20). These data indicated that both **1** and **3** are *threo*- configurations between C-8’ and C-9’. Meanwhile, the key NOESY correlations of H-4’/H_3_-10’/H-8’ of **2** (Figures S32‒S36) and **4** (Figures S61−S65) at 25 °C and/or at 1 °C suggested *erythro*- configuration between C-8’ and C-9’. Thus, the absolute configurations of compounds **1**/**3** and **2**/**4** were determined as (19*S*, 5’*S*, 6’*R*, 8’*S*, 9’*S*) and (19*S*, 5’*S*, 6’*R*, 8’*R*, 9’*S*), respectively.

The cytotoxic activity of compounds **1**–**6** against 26 human cancer cell lines and two normal ones were evaluated by the Cell Titer Glo (CTG) assay [24] (the names of cell lines and experimental details could be found in Supporting Information). The cytotoxic assay displayed some interesting results (Table 2). Compound **6** showed the most broad-spectrum of cytotoxic activities against 16 cancer cells with IC_50_ values ranging from 2.54 to 9.79 μM, because the epoxide could alkylate DNA by a nucleophilic addition with the base of DNA or sulfydryl of protein. However, the nucleophilic addition of epoxide of **6** with **5** preferred to reduce its activity against cancer cells. Among the four conjugates, compound **1** showed the most wide spectrum of cytotoxicity against 10 cancer cells with IC_50_ values ranging from 3.10 to 11.32 μM (Table 2). Compounds **2**, **4**, and **5** showed a selective cytotoxicity against U251 (glioblastoma cell line), while compound **3** selectively inhibited A673 (rhabdomyoma cell line), U87 (glioblastoma cell line), and Hep3B (human liver cancer cell line) (Table 2). More interestingly, the conjugation between **5** and **6** enhanced the inhibitory effects on two tumorigenic glioblastoma cell lines, U87 and U251. The capacity for migration and invasion of U87 cells are greater than those of U251 cells [25]. New compounds **1** and **3** with 8’*S*-configuration displayed cytotoxic activity against U87 cells while compounds **2** and **4** with 8’*R*-configuration were active against U251 cells. Moreover, compounds **2**–**4** exhibited low cytotoxicity for two human normal cells, HEK-293F (human embryonic kidney-293F cell line) and L02 (human liver cell line). U251 and U87 are two common experimental glioblastoma cell lines with different phenotypes. U251 expressed lower levels of metastasis-associated lung adenocarcinoma transcript 1 (MALAT1) but higher levels of epidermal growth factor receptor variant III (EGFRvIII), human epidermal growth factor receptor 2 (HER2) and cytochrome P450 17A1 (CYP17A1) than those of U87 [26,27,28]. This investigation provides novel and bioactive compounds against human glioblastoma cells in noncytotoxic concentration to human normal cells.

## 3. Experimental Section

### 3.1. General Experimental Procedures

Specific rotations were obtained on a JASCO P-1020 digital polarimeter (JASCO Corporation, Tokyo, Japan) equipped with a halogen lamp (589 nm). UV spectra were recorded on Beckman DU 640 spectrophotometer. ECD (electronic circular dichroism) spectra were measured on JASCO J-815 spectropolarimeter (JASCO Corporation). IR spectra were obtained on a Nicolet Nexus 470 spectrophotometer (Thermo Nicolet Corporation, Madison, WI, USA) in KBr discs. NMR spectra were recorded on a JEOL JNM-ECP 600 spectrometer (JEOL Corporation, Akishima, Japan) and Varian System 500 spectrometer (Varian, Palo Alto, CA, USA), and the corresponding residual solvent signals (*δ*_H/C_ 2.50/39.52 for DMSO-*d*_6_) were used to reference the chemical shifts. HRESIMS spectra were recorded using the Q-TOF (quadrupole time-of-flight) Ultima Global GAA076 LC mass spectrometer (Waters Asia Ltd., Singapore). Semi-preparative HPLC was performed using an ODS (octadecylsilyl) column (YMC-pak ODS-A, 10 × 250 mm, 5 μm, 4 mL/min). Thin-layer chromatography (TLC) was performed on plates precoated with silica gel GF_254_ (10–40 μm). Column chromatography (CC) was performed over silica gel (200–300 mesh, Qingdao Marine Chemical Factory, Qingdao, China) and Sephadex LH-20 (Amersham Biosciences, Uppsala, Sweden). Vacuum-liquid chromatography (VLC) was carried out over silica gel H (Qingdao Marine Chemical Factory, Qingdao, China). Sea salt used was made from the evaporation of sea water collected in Laizhou Bay (Weifang Haisheng Chemical Factory, Weifang, China).

### 3.2. Fungus Material

The fungus *Aspergillus ochraceus* LCJ11-102 was isolated from the coral *Dichotella gemmacea* (Valenciennes) collected in Lingao, Hainan province, China. The coral sample (LCJ-11) was cut into small pieces, which were stamped onto the agar plates using potato dextrose agar (PDA) media. Fungal colonies were selected and streaked to purity using the same agar media. *A. ochraceus* LCJ11-102 was identified according to its morphological characteristics and 18S rRNA gene sequences (GenBank accession No. GU227340).

### 3.3. Fermentation and Extraction

The fungus *Aspergillus ochraceus* LCJ11-102 (~8 × 10^7^ conidiophores) was cultivated in 1000 mL Erlenmeyer flasks containing 300 mL fermentation media (10 g soluble starch, 1 g peptone, and 2 g sea salt dissolved in 1 L of tap water, pH 7.5). The flasks were statically incubated at 28 °C for 30 days. The whole fermentation broth (90 L) was filtered by cheesecloth to separate into mycelia and filtrate. The filtrate was extracted with ethyl acetate (EtOAc) three times (100 L each time), yielding 11.0 g of extract. The mycelia were extracted with acetone, yielding 37.0 g of extract. The extracts of the filtrate and the mycelia were combined after HPLC analysis.

### 3.4. Purification

The extract (48.0 g) was separated into eight fractions (Fr.1–Fr.8) on a silica gel VLC column using step gradient elution with CH_2_Cl_2_–petroleum ether (50–100%) and then with MeOH–CH_2_Cl_2_ (0–50%). Fraction 4 (2.1 g) was separated into 11 subfractions (Fr.4.1–Fr.4.11) by chromatography on a silica gel column using stepwise gradient elution with 5–50% acetone/petroleum ether. Fr.4.8 (350 mg) was recrystallized from MeOH to give compound **6** (115 mg). Fraction 8 (3.2 g) was separated into 10 subfractions (Fr.8.1–Fr.8.10) by chromatography on a silica gel column using stepwise gradient elution with 10–50% acetone/petroleum ether. Fr.8.8 (602 mg) was further separated into eight subfractions (Fr.8.8.1–Fr.8.8.8) on Sephadex LH-20, eluting with MeOH. Fr.8.8.6 (50 mg) was purified by HPLC on an ODS column using the solvent system of 45% CH_3_CN–H_2_O to yield compounds **1** (5.4 mg, *t*_R_13.2 min), **2** (5.0 mg, *t*_R_14.0 min), **3** (6.1 mg, *t*_R_14.5 min), and **4** (3.2 mg, *t*_R_13.5 min). Fr.8.8.8 (215 mg) was purified by HPLC on an ODS column using the solvent system of 50% MeOH–H_2_O to yield **5** (170.0 mg, *t*_R_ 13.6 min).

**Ochrazepine A (1)**: Reddish-brown powder; ${\left[\alpha \right]}_{D}^{25}$ −110.0 (*c* 0.1, MeOH); UV (MeOH) *λ*_max_ (log *ε*) 206 (4.46), 227 (4.62) nm; IR (KBr) *ν*_max_ 3386, 2924, 1684, 1618, 1680, 1456, 1385, 1279, 1060, 1024, 779 cm^−1^; ECD (0.5 mM, MeOH) *λ*_max_ (Δ*ε*) 218 (+61.8), 240 (−35.7), 265.5 (+0.9), 296.5 (+2.0), 307 (−2.5) nm. ^1^H and ^13^C NMR data, see Table 1; HRESIMS *m*/*z* 508.1718 [M + H]^+^ (calcd. for C_26_H_26_N_3_O_8_, 508.1714).

**Ochrazepine B (2)**: Reddish-brown powder; ${\left[\alpha \right]}_{D}^{25}$ −66.6 (*c* 0.1, MeOH); UV (MeOH) *λ*_max_ (log *ε*) 203 (4.05), 224 (3.96) nm; IR (KBr) *ν*_max_ 3396, 2981, 1683, 1613, 1515, 1452, 1384, 1279, 1208, 1136, 1062, 1024, 778 cm^−1^; ECD (0.5 mM, MeOH) *λ*_max_ (Δ*ε*) 218 (+79.6), 238 (−54.5), 265 (+5.8), 295.5 (+3.7), 311 (−1.5) nm. ^1^H and ^13^C NMR data, see Table 1; HRESIMS *m*/*z* 508.1717 [M + H]^+^ (calcd. for C_26_H_26_N_3_O_8_, 508.1714). 

**Ochrazepine C (3)**: Reddish-brown powder; ${\left[\alpha \right]}_{D}^{25}$ −144.4 (*c* 0.1, MeOH); UV (MeOH) *λ*_max_ (log *ε*) 201 (4.45), 227 (4.50) nm; IR (KBr) *ν*_max_ 3405, 2983, 1671, 1610, 1513, 1456, 1363, 1272, 1219, 1060, 777 cm^−1^; ECD (0.5 mM, MeOH) *λ*_max_ (Δ*ε*) 218 (+80.0), 238 (−48.7), 269 (+0.7), 296.5 (+3.0), 313 (−7.7) nm. ^1^H and ^13^C NMR data, see Table 1; HRESIMS *m*/*z* 508.1721 [M + H]^+^ (calcd. for C_26_H_26_N_3_O_8_, 508.1714).

**Ochrazepine D (4)**: Reddish-brown powder; ${\left[\alpha \right]}_{D}^{25}$ −101.1 (*c* 0.1, MeOH); UV (MeOH) *λ*_max_ (log *ε*) 201 (3.80), 226 (3.97) nm; IR (KBr) *ν*_max_ 3399, 2983, 1671, 1611, 1513, 1453, 1382, 1277, 1219, 1061, 778 cm^−1^. ECD (0.5 mM, MeOH) *λ*_max_ (Δ*ε*) 218 (+68.8), 238 (−53.1), 269 (+3.8), 295.5 (+1.2), 312 (−8.0) nm. ^1^H and ^13^C NMR data, see Table 1; HRESIMS *m*/*z* 508.1711 [M + H]^+^ (calcd. for C_26_H_26_N_3_O_8_, 508.1714).

### 3.5. X-Ray Diffraction Data of Compound ***6***

Colorless crystal (MeOH), C_9_H_12_O_4_, 293 (2) K, orthorhombic. Space group: *P*2(1)2(1)2(1) with *a* = 4.4269(4) Å, *b* = 14.2731(10) (5) Å, *c* = 14.9494(14) Å, *V* = 944.59(14) Å^3^. *Z* = 4, *D*_calcd_ = 1.295 mg/m^3^, *μ* = 0.861 mm^−1^, and *F* (000) = 392. Crystal size: 0.30 × 0.20 × 0.11 mm^3^. Reflections collected/unique: 1307/1072 [*R*(int) = 0.0437]; Final R indices [*I* > 2 sigma (*I*)]: R_1_ = 0.0541, wR_2_ = 0.1122. Absolute structure parameter: 0.0(5). The data was obtained on a Bruker Smart-1000 CCD area detector diffractometer with graphite monochromated Cu-Kα radiation (*λ* = 1.54178 Å). Structures were expanded using full-matrix least-squares difference Fourier techniques and solved by direct methods (SHELXS-97). The deposited number of **6** in the Cambridge Crystallographic Data Centre is CCDC 1876311.

### 3.6. Absolute Configuration Determination of Alanine of ***5*** by Marfey’s Method

A solution of compound **5** (1.0 mg) in 6.0 M HCl (0.6 mL) was placed in an ampoule flask and then sealed and heated at 105 °C for 17 h. The solution was evaporated to dryness and the residue was dissolved in H_2_O (250 μL). Standard L-Ala and (D,L)-Ala were separately made into the aqueous solutions (50 mM). Then, each solution (50 μL) was treated with 200 μL of 1% solution of L-FDAA in acetone followed by 1.0 M NaHCO_3_ (40 μL). The reaction mixture was incubated at 45 °C for 1 h and quenched with 2.0 M HCl (10 μL). The FDAA-derivatives of the hydrolysates and standard amino acids were subjected to LC-MS analysis. The retention times for the FDAA-derivatives of the hydrolysate of **5**, standard L-Ala, and (D,L)-Ala were 2.73, 2.70, and 2.90/2.70 min, respectively (Figure S66).

### 3.7. Synthesis of ***1***–***4*** from ***5*** and ***6***

K_2_CO_3_ (2.06 mg, 0.01 mmol) was added to a solution of **5** (12.92 mg, 0.04 mmol) and **6** (7.36 mg, 0.04 mmol) in MeCN (5.0 mL) at room temperature. The mixture was stirred for 72 h and then saturated NH_4_Cl solution was added to adjust the pH value to pH 6. The obtained reaction mixture was extracted with EtOAc (3 × 5 mL). The EtOAc solution was combined and evaporated to dryness and the product was subjected to LC-MS analysis (Figure S1) and then purified by HPLC on a C_18_ column using the solvent system of 45% MeCN-H_2_O to yield compounds **1** (2.63 mg, *t*_R_13.2 min, 13.0% yield), **2** (2.51 mg, *t*_R_ 14.0 min, 12.4% yield), **3** (2.59 mg, *t*_R_ 14.5 min, 12.8% yield), and **4** (2.50 mg, *t*_R_ 13.5 min, 12.3% yield). These products were also identified as the same as the natural ones by the same NMR and specific rotations.

### 3.8. Reaction of Compounds ***5*** and ***6*** under Neutral and Acidic Conditions

Two solutions of **5** (1 mg, 3 μmol) and **6** (1 mg, 5 μmol) in MeCN (0.5 mL) were added CF_3_CO_2_H (10 μL, 0.13 μmol) and were not added CF_3_CO_2_H, respectively. The reaction mixtures were stirred for 72 h and then were subjected to HPLC-MS analysis (Figures S2 and S3).

## 4. Conclusions

In summary, we identified four new circumdatin-aspyrone conjugates (**1**–**4**) from the coral-associated *Aspergillus ochraceus* LCJ11-102. These compounds could be obtained from the nucleophilic addition of 2-hydroxycircumdatin C (**5**) with aspyrone (**6**) under basic condition. Compounds **1** and **6** showed a relatively-broad spectrum of cytotoxic activity against human cancer cells. The observation of the selective cytotoxicities for compounds **2**, **4**, and **5** against U251 cells and **3** against A673, U87, and Hep3B cells as well as their low cytotoxicity to human normal cells (HEK-293F and L-02) indicated their potential use in the discovery of new anti-cancer drugs for the treatment of glioblastoma (U87 and U251), rhabdomyoma (A673), and liver cancer (Hep3B).

## Figures and Tables

**Table 1:** ^1^H and ^13^C NMR data for ochrazepines A−D (**1**−**4**) in DMSO-*d*_6_.

No.	1 ^a^		2 ^a^		3 ^a^		4 ^b^	
	*δ*_C_	*δ*_H_, mult. (*J* in Hz)	*δ*_C_	*δ*_H_, mult. (*J* in Hz)	*δ*_C_	*δ*_H_, mult. (*J* in Hz)	*δ*_C_	*δ*_H_, mult. (*J* in Hz)
2	166.5, C		166.5, C		166.4, C		166.4, C	
3	122.6, C		122.6, C		124.5, C		124.8, C	
4	113.1, CH	7.03, s	113.3, CH	7.08, s	114.4, CH	7.10, s	114.7, CH	7.15, s
5	145.6, C		145.3, C		145.9, C		146.5, C	
6	148.7, C		148.7, C		146.9, C		147.4 C	
7	115.8, CH	6.99, s	115.7, CH	7.00, s	114.0, CH	6.75, s	114.3, CH	6.90, s
8	127.3, C		127.4, C		125.1, C		124.9, C	
10	161.1, C		161.1, C		161.1, C		161.0, C	
11	121.0, C		120.9, C		120.9, C		120.9, C	
12	126.9, CH	8.17, dd (7.9, 1.5)	126.8, CH	8.16, d (7.9)	126.6, CH	8.12, dd (7.9, 1.5)	126.9, CH	8.17, dd (8.0, 1.2)
13	127.4, CH	7.58, td (8.1, 1.2)	127.4, CH	7.58, t (7.5)	127.4, CH	7.61, td (8.2, 1.2)	127.3, CH	7.56, t (7.9)
14	135.1, CH	7.89, td (8.4, 1.5)	135.0, CH	7.89, t (7.6)	135.1, CH	7.84, td (8.4, 1.5)	135.1, CH	7.87, td (8.4, 1.5)
15	127.4, CH	7.72, d (8.2)	127.4, CH	7.72, d (8.1)	127.5, CH	7.72, d (7.6)	127.4, CH	7.71, d (8.0)
16	145.9, C		145.9, C		146.1, C		145.9, C	
18	156.8, C		156.9, C		157.0, C		157.0, C	
19	49.5, CH	4.32, m	49.5, CH	4.33, m	49.5, CH	4.35, m	49.5, CH	4.28, m
20	14.9, CH_3_	1.51, d (6.7)	14.9, CH_3_	1.49, d (6.7)	15.0, CH_3_	1.49, d (6.7)	14.9, CH_3_	1.50, d (6.7)
2’	162.9, C		162.9, C		163.5, C		162.9, C	
3’	127.4, C		127.4, C		126.7, C		126.5, C	
4’	146.4, CH	6.83, d (2.7)	147.4, CH	6.86, brs	149.0, CH	6.85, d (1.7)	147.8, CH	6.74, s
5’	65.9, CH	4.19, d (7.8)	66.6, CH	4.13, d (9.5)	67.0, CH	4.23, d (8.8)	66.6, CH	4.03, d (9.8)
6’	79.1, CH	4.27, m	78.1, CH	4.23, m	78.5, CH	4.60, m	78.0, CH	4.16, m
7’	17.9, CH_3_	1.32, d (6.4)	17.6, CH_3_	1.34, d (6.2)	17.8, CH_3_	1.40, d (6.4)	17.4, CH_3_	1.26, d (6.3)
8’	79.1, CH	4.88, d (4.2)	78.1, CH	5.10, d (4.3)	78.7, CH	4.66, d (4.6)	78.2, CH	4.97, d (4.6)
9’	68.1, CH	3.89, qd (6.5, 4.2)	68.1, CH	3.99, qd (6.4, 4.3)	68.2, CH	3.87, qd (6.4, 4.6)	67.9, CH	3.99, qd (6.5, 4.6)
10’	19.6, CH_3_	1.19, d (6.4)	18.2, CH_3_	1.12, d (6.4)	19.5, CH_3_	1.11, d (6.4)	18.3, CH_3_	1.11, d (6.4)
1-NH		8.55, d (5.8)		8.57, d (5.8)		8.57, d (5.8)		8.56, d (5.5)
6/5-OH		9.77, s		9.90, s		9.52, s		
5’-OH		5.83, s		5.91, s		5.81, s		5.81, s
9’-OH		5.21, s		5.16, s		5.02, s		5.02, s

^a^ Spectra were recorded at 600 MHz for ^1^H and 150 MHz for ^13^C. ^b^ Spectra were recorded at 500 MHz for ^1^H and 125 MHz for ^13^C.

**Table 2:** The cytotoxicity of compounds **1**–**6** against human cancer cell lines (IC_50_, μM) by Cell Titer Glo (CTG) assay ^a^.

	1	2	3	4	5	6	Adramycin
MV-4-11	3.94	>100	>100	>100	>100	2.54	0.16
K562	6.05	>100	>100	>100	>100	5.22	0.02
A673	3.10	>100	8.24	>100	>100	8.55	0.13
U87	8.67	>100	9.04	>100	>100	>100	0.12
A549	9.62	>100	>100	>100	>100	>100	0.10
N87	6.10	>100	>100	>100	>100	4.57	0.05
H1299	7.14	>100	>100	>100	>100	5.83	0.49
HUCCT1	11.32	>100	>100	>100	>100	9.79	0.05
B16F10	11.22	>100	>100	>100	>100	5.89	0.02
Karpass299	5.89	>100	>100	>100	>100	2.57	0.39
U251	>100	9.91	>100	8.26	8.95	>100	0.19
Hep3B	>100	>100	10.28	>100	>100	5.48	17.58
A431	>100	>100	>100	>100	>100	5.92	0.17
143B	>100	>100	>100	>100	>100	6.32	0.10
MKN-45	>100	>100	>100	>100	>100	5.79	0.20
H1975	>100	>100	>100	>100	>100	2.99	0.09
HL60	>100	>100	>100	>100	>100	6.89	0.21
DU145	>100	>100	>100	>100	>100	5.61	0.05
SPC-A1	>100	>100	>100	>100	>100	9.51	0.19
HEK-293F ^b^	12.91	73.96	76.03	54.58	>100	50.35	0.05
L02 ^b^	27.42	>100	>100	>100	>100	14.2	0.10

^a^ Compounds **1**−**6** exhibited no cytotoxicity (IC_50_ > 100 μM) against the cancer cell lines HCC1954, MCF-7, HCT116, BT474, H2228, MDA-MB-231, and MDA-MB-468. ^b^ Human normal cells.

## Supplementary Materials

The following are available online at https://www.mdpi.com/1660-3397/17/7/400/s1, Cytotoxicity Assay, Figure S1: LC-MS profile for the synthesis of **1**–**4** from **5** and **6** under basic condition, Figure S2: LC-MS profile of the reaction mixture of **5** and **6** under neutral condition, Figure S3: LC-MS profile of the reaction mixture of **5** and **6** under acidic condition, Figure S4: HRESIMS spectrum of ochrazepine A (**1**), Figure S5: ^1^H-NMR spectrum of ochrazepine A (**1**) in DMSO-*d*_6_ (1), Figure S6: ^1^H-NMR spectrum of ochrazepine A (**1**) in DMSO-*d*_6_ (2), Figure S7: ^13^C-NMR spectrum of ochrazepine A (**1**) in DMSO-*d*_6_, Figure S8: HMQC spectrum of ochrazepine A (**1**) in DMSO-*d*_6_ (1), Figure S9: HMQC spectrum of ochrazepine A (**1**) in DMSO-*d*_6_ (2), Figure S10: ^1^H-^1^H COSY spectrum of ochrazepine A (**1**) in DMSO-*d*_6_ (1), Figure S11: ^1S^H-^1^H COSY spectrum of ochrazepine A (**1**) in DMSO-*d*_6_ (2), Figure S12: HMBC spectrum of ochrazepine A (**1**) in DMSO-*d*_6_ (1), Figure S13: HMBC spectrum of ochrazepine A (**1**) in DMSO-*d*_6_ (2), Figure S14: HMBC spectrum of ochrazepine A (**1**) in DMSO-*d*_6_ (3), Figure S15: HMBC spectrum of ochrazepine A (**1**) in DMSO-*d*_6_ (4), Figure S16: NOESY spectrum of ochrazepine A (**1**) in DMSO-*d*_6_ at 25 °C (1), Figure S17: NOESY spectrum of ochrazepine A (**1**) in DMSO-*d*_6_ at 25 °C (2), Figure S18: ^1^H-NMR spectrum of ochrazepine A (**1**) in MeOH-*d*_4_ at 1 °C, Figure S19: NOESY spectrum of ochrazepine A (**1**) in MeOH-*d*_4_ at 1 °C (1), Figure S20: NOESY spectrum of ochrazepine A (**1**) in MeOH-*d*_4_ at 1 °C (2), Figure S21: HRESIMS spectrum of ochrazepine B (**2**), Figure S22: ^1^H-NMR spectrum of ochrazepine B (**2**) in DMSO-*d*_6_ (1), Figure S23: ^1^H-NMR spectrum of ochrazepine B (**2**) in DMSO-*d*_6_ (2), Figure S24: ^13^C-NMR spectrum of ochrazepine B (**2**) in DMSO-*d*_6_, Figure S25: HMQC spectrum of ochrazepine B (**2**) in DMSO-*d*_6_ (1), Figure S26: HMQC spectrum of ochrazepine B (**2**) in DMSO-*d*_6_ (2), Figure S27: ^1^H-^1^H COSY spectrum of ochrazepine B (**2**) in DMSO-*d*_6_ (1), Figure S28: ^1^H-^1^H COSY spectrum of ochrazepine B (**2**) in DMSO-*d*_6_ (2), Figure S29: HMBC spectrum of ochrazepine B (**2**) in DMSO-*d*_6_ (1), Figure S30: HMBC spectrum of ochrazepine B (**2**) in DMSO-*d*_6_ (2), Figure S31: HMBC spectrum of ochrazepine B (**2**) in DMSO-*d*_6_ (3), Figure S32: NOESY spectrum of ochrazepine B (**2**) in DMSO-*d*_6_ at 25 °C (1), Figure S33: NOESY spectrum of ochrazepine B (**2**) in DMSO-*d*_6_ at 25 °C (2), Figure S34: ^1^H-NMR spectrum of ochrazepine B (**2**) in MeOH-*d*_4_ at 1 °C, Figure S35: NOESY spectrum of ochrazepine B (**2**) in MeOH-*d*_4_ at 1 °C (1), Figure S36: NOESY spectrum of ochrazepine B (**2**) in MeOH-*d*_4_ at 1 °C (2), Figure S37: HRESIMS spectrum of ochrazepine C (3), Figure S38: ^1^H-NMR spectrum of ochrazepine C (**3**) in DMSO-*d*_6_, Figure S39: ^13^C-NMR spectrum of ochrazepine C (**3**) in DMSO-*d*_6_, Figure S40: HMQC spectrum of ochrazepine C (**3**) in DMSO-*d*_6_ (1), Figure S41: HMQC spectrum of ochrazepine C (**3**) in DMSO-*d*_6_ (2), Figure S42: ^1^H-^1^H COSY spectrum of ochrazepine C (**3**) in DMSO-*d*_6_ (1), Figure S43: ^1^H-^1^H COSY spectrum of ochrazepine C (**3**) in DMSO-*d*_6_ (2), Figure S44: HMBC spectrum of ochrazepine C (**3**) in DMSO-*d*_6_ (1), Figure S45: HMBC spectrum of ochrazepine C (**3**) in DMSO-*d*_6_ (2), Figure S46: HMBC spectrum of ochrazepine C (**3**) in DMSO-*d*_6_ (3), Figure S47: NOESY spectrum of ochrazepine C (**3**) in DMSO-*d*_6_ at 25 °C (1), Figure S48: NOESY spectrum of ochrazepine C (**3**) in DMSO-*d*_6_ at 25 °C (2), Figure S49: ^1^H-NMR spectrum of ochrazepine C (**3**) in MeOH-*d*_4_ at 1 °C, Figure S50: NOESY spectrum of ochrazepine C (**3**) in MeOH-*d*_4_ at 1 °C, Figure S51: HRESIMS spectrum of ochrazepine D (**4**), Figure S52: ^1^H-NMR spectrum of ochrazepine D (**4**) in DMSO-*d*_6_, Figure S53: ^13^C-NMR spectrum of ochrazepine D (**4**) in DMSO-*d*_6_, Figure S54: HSQC spectrum of ochrazepine D (**4**) in DMSO-*d*_6_ (1), Figure S55: HSQC spectrum of ochrazepine D (**4**) in DMSO-*d*_6_ (2), Figure S56: ^1^H-^1^H COSY spectrum of ochrazepine D (**4**) in DMSO-*d*_6_ (1), Figure S57: ^1^H-^1^H COSY spectrum of ochrazepine D (**4**) in DMSO-*d*_6_ (2), Figure S58: HMBC spectrum of ochrazepine D (**4**) in DMSO-*d*_6_ (1), Figure S59: HMBC spectrum of ochrazepine D (**4**) in DMSO-*d*_6_ (2), Figure S60: HMBC spectrum of ochrazepine D (**4**) in DMSO-*d*_6_ (3), Figure S61: NOESY spectrum of ochrazepine D (**4**) in DMSO-*d*_6_ at 25 °C (1), Figure S62: NOESY spectrum of ochrazepine D (**4**) in DMSO-*d*_6_ at 25 °C (2), Figure S63: ^1^H-NMR spectrum of ochrazepine D (**4**) in MeOH-*d*_4_ at 1 °C, Figure S64: NOESY spectrum of ochrazepine D (**4**) in MeOH-*d*_4_ at 1 °C (1), Figure S65: NOESY spectrum of ochrazepine D (**4**) in MeOH-*d*_4_ at 1 °C (2), Figure S66: The determination of the absolute configuration of **5** by Marfey’s method, Figure S67: ECD curves of compounds **1**–**4**.
