# Supplementary material for: Circumdatin-Aspyrone Conjugates from the Coral-Associated Aspergillus ochraceus LCJ11-102
Source: Mar Drugs. 2019 Jul 6;17(7):400. doi: 10.3390/md17070400 (PMC6669671; doi:10.3390/md17070400)

Supplementary Material

Circumdatin-aspyrone conjugates from the coral-associated *Aspergillus ochraceus* LCJ11-102

Yaqin Fan[1], Yalin Zhou[1], Yuqi Du[1], Yi Wang[1], Peng Fu[1,2]*, Weiming Zhu[1,2]*

[1] Key Laboratory of Marine Drugs, Ministry of Education of China, School of Medicine and Pharmacy, Ocean University of China, Qingdao 266003, China; fanyaqin.826@163.com (Y.F.), zhouyalincpu@tom.com (Y.Z.), mystagedu@foxmail.com (Y.D.), wangyi0213@ouc.edu.cn (Y.W.)

[2] Open Studio for Druggability Research of Marine Natural Products, Laboratory for Marine Drugs and Bioproducts, Pilot National Laboratory for Marine Science and Technology (Qingdao), Qingdao 266003, China

* Correspondence: fupeng@ouc.edu.cn (P.F.); weimingzhu@ouc.edu.cn (W.Z.);
Tel./Fax: +86-532-8203-1268 (W.Z.)

List of Supplementary Material

Cytotoxicity Assay……………………………………………………………………………………..S4
Figure S1. LC-MS profile for the synthesis of **1**–**4** from **5** and **6** under basic condition……………………………S4
Figure S2. LC-MS profile of the reaction mixture of **5** and **6** under neutral condition………………………………S5
Figure S3. LC-MS profile of the reaction mixture of **5** and **6** under acidic condition………………………………S5
Figure S4. HRESIMS spectrum of ochrazepine A (**1**) ……………………………………………………………S6
Figure S5. ^{1}H-NMR spectrum of ochrazepine A (**1**) in DMSO-d_6 (1)……………………………...……………S7
Figure S6. ^{1}H-NMR spectrum of ochrazepine A (**1**) in DMSO-d_6 (2)……………………………...……………S8
Figure S7. ^{13}C-NMR spectrum of ochrazepine A (**1**) in DMSO-d_6……………………………….………S9
Figure S8. HMQC spectrum of ochrazepine A (**1**) in DMSO-d_6 (1)………………………………………S10
Figure S9. HMQC spectrum of ochrazepine A (**1**) in DMSO-d_6 (2)………………………………………S11
Figure S10. ^{1}H-^{1}H COSY spectrum of ochrazepine A (**1**) in DMSO-d_6 (1)……………………..…………S12
Figure S11. ^{1}H-^{1}H COSY spectrum of ochrazepine A (**1**) in DMSO-d_6 (2)……………………..…………S13
Figure S12. HMBC spectrum of ochrazepine A (**1**) in DMSO-d_6 (1)…………………………………………S14
Figure S13. HMBC spectrum of ochrazepine A (**1**) in DMSO-d_6 (2)…………………………………………S15
Figure S14. HMBC spectrum of ochrazepine A (**1**) in DMSO-d_6 (3)…………………………………………S16
Figure S15. HMBC spectrum of ochrazepine A (**1**) in DMSO-d_6 (4)…………………………………………S17
Figure S16. NOESY spectrum of ochrazepine A (**1**) in DMSO-d_6 at 25 °C (1)……………………………………S18
Figure S17. NOESY spectrum of ochrazepine A (**1**) in DMSO-d_6 at 25 °C (2)……………………………………S19

Figure S18. ^{1}H-NMR spectrum of ochrazepine A (**1**) in MeOH-d_4 at 1 °C..S20
Figure S19. NOESY spectrum of ochrazepine A (**1**) in MeOH-d_4 at 1 °C (1)..S21
Figure S20. NOESY spectrum of ochrazepine A (**1**) in MeOH-d_4 at 1 °C (2)..S22
Figure S21. HRESIMS spectrum of ochrazepine B (**2**) S23
Figure S22. ^{1}H-NMR spectrum of ochrazepine B (**2**) in DMSO-d_6 (1)..S24
Figure S23. ^{1}H-NMR spectrum of ochrazepine B (**2**) in DMSO-d_6 (2)..S25
Figure S24. ^{13}C-NMR spectrum of ochrazepine B (**2**) in DMSO-d_6..S26
Figure S25. HMQC spectrum of ochrazepine B (**2**) in DMSO-d_6 (1)..S27
Figure S26. HMQC spectrum of ochrazepine B (**2**) in DMSO-d_6 (2)..S28
Figure S27. ^{1}H-^{1}H COSY spectrum of ochrazepine B (**2**) in DMSO-d_6 (1)..S29
Figure S28. ^{1}H-^{1}H COSY spectrum of ochrazepine B (**2**) in DMSO-d_6 (2)..S30
Figure S29. HMBC spectrum of ochrazepine B (**2**) in DMSO-d_6 (1)..S31
Figure S30. HMBC spectrum of ochrazepine B (**2**) in DMSO-d_6 (2)..S32
Figure S31. HMBC spectrum of ochrazepine B (**2**) in DMSO-d_6 (3)..S33
Figure S32. NOESY spectrum of ochrazepine B (**2**) in DMSO-d_6 at 25 °C (1)..S34
Figure S33. NOESY spectrum of ochrazepine B (**2**) in DMSO-d_6 at 25 °C (2)..S35
Figure S34. ^{1}H-NMR spectrum of ochrazepine B (**2**) in MeOH-d_4 at 1 °C..S36
Figure S35. NOESY spectrum of ochrazepine B (**2**) in MeOH-d_4 at 1 °C (1)..S37
Figure S36. NOESY spectrum of ochrazepine B (**2**) in MeOH-d_4 at 1 °C (2)..S38
Figure S37. HRESIMS spectrum of ochrazepine C (**3**) S39
Figure S38. ^{1}H-NMR spectrum of ochrazepine C (**3**) in DMSO-d_6..S40
Figure S39. ^{13}C-NMR spectrum of ochrazepine C (**3**) in DMSO-d_6..S41
Figure S40. HMQC spectrum of ochrazepine C (**3**) in DMSO-d_6 (1)..S42
Figure S41. HMQC spectrum of ochrazepine C (**3**) in DMSO-d_6 (2)..S43
Figure S42. ^{1}H-^{1}H COSY spectrum of ochrazepine C (**3**) in DMSO-d_6 (1)..S44
Figure S43. ^{1}H-^{1}H COSY spectrum of ochrazepine C (**3**) in DMSO-d_6 (2)..S45
Figure S44. HMBC spectrum of ochrazepine C (**3**) in DMSO-d_6 (1)..S46
Figure S45. HMBC spectrum of ochrazepine C (**3**) in DMSO-d_6 (2)..S47
Figure S46. HMBC spectrum of ochrazepine C (**3**) in DMSO-d_6 (3)..S48
Figure S47. NOESY spectrum of ochrazepine C (**3**) in DMSO-d_6 at 25 °C (1)..S49
Figure S48. NOESY spectrum of ochrazepine C (**3**) in DMSO-d_6 at 25 °C (2)..S50
Figure S49. ^{1}H-NMR spectrum of ochrazepine C (**3**) in MeOH-d_4 at 1 °C..S51
Figure S50. NOESY spectrum of ochrazepine C (**3**) in MeOH-d_4 at 1 °C..S52
Figure S51. HRESIMS spectrum of ochrazepine D (**4**) S53
Figure S52. ^{1}H-NMR spectrum of ochrazepine D (**4**) in DMSO-d_6..S54
Figure S53. ^{13}C-NMR spectrum of ochrazepine D (**4**) in DMSO-d_6..S55

Figure S54. HSQC spectrum of ochrazepine D (**4**) in DMSO-d_6 (1)……………………………………………S56
Figure S55. HSQC spectrum of ochrazepine D (**4**) in DMSO-d_6 (2)……………………………………………S57
Figure S56. ^{1}H-^{1}H COSY spectrum of ochrazepine D (**4**) in DMSO-d_6 (1)……………..………………………S58
Figure S57. ^{1}H-^{1}H COSY spectrum of ochrazepine D (**4**) in DMSO-d_6 (2)……………..………………………S59
Figure S58 HMBC spectrum of ochrazepine D (**4**) in DMSO-d_6 (1)………………...………………………S60
Figure S59. HMBC spectrum of ochrazepine D (**4**) in DMSO-d_6 (2)………………...………………………S61
Figure S60. HMBC spectrum of ochrazepine D (**4**) in DMSO-d_6 (3)………………...………………………S62
Figure S61. NOESY spectrum of ochrazepine D (**4**) in DMSO-d_6 at 25°C (1)…………………………………S63
Figure S62. NOESY spectrum of ochrazepine D (**4**) in DMSO-d_6 at 25°C (2)…………………………………S64
Figure S63. ^{1}H-NMR spectrum of ochrazepine D (**4**) in MeOH-d_4 at 1 °C………………………………………S65
Figure S64. NOESY spectrum of ochrazepine D (**4**) in MeOH-d_4 at 1 °C (1)………………………...………S66
Figure S65. NOESY spectrum of ochrazepine D (**4**) in MeOH-d_4 at 1 °C (2)………………………...………S67
Figure S66. The determination of the absolute configuration of **5** by Marfey's method ………………………………S68
Figure S67. ECD curves of compounds **1**–**4**……………………………………………………….………………S68

Cytotoxicity Assay. By the Cell Titer Glo (CTG) assay,[24] compounds **1**–**6** were evaluated for cytotoxicity against A431 (Epidermoid carcinoma cell line), A673 (rhabdomyoma cell line), U87 (glioblastoma cell line), U251 (glioblastoma cell line), HCC1954 (grade 3 invasive ductal carcinoma cell line), MCF-7 (human breast adenocarcinoma cell line), MKN-45 (human gastric cancer cell line), Hep3B (human liver cancer cell line), H1975 (human non-small cell lung carcinoma with L858R and T790M mutation cell line), DU145 (human prostate cancer cell line), MV-4-11 (biphenotypic B myelomonocytic leukemia cell line), K562 (human erythroleukemic cell line), A549 (lung cancer cell line), N87 (gastric carcinoma cell line), H1299 (human non-small cell lung carcinoma cell line), HUCCT1 (bile duct carcinoma cell line), 143B (human bone osteosarcoma cell line), B16F10 (highly metastatic mouse melanoma cell line), SPC-A1 (human lung cancer cell line overexpressing maspin cell line), HCT116 (colon carcinoma cell line), BT474 (hormone-sensitive breast cancer cell line), H2228 (non-small cell lung cancer cell line), MDA-MB-231 (breast cancer cell line), MDA-MB-468 (basal breast cancer cell line), Karpass299 (human T cell lymphoma cell line), HL-60 (human promyelocytic leukemia cell line), HEK-293F(human embryonic kidney-293F cell line) and L02 (human liver cell line). In the CTG assay, 26 cell lines above were grown in DMEM supplemented with 10% fetal bovine serum and 1% penicillin-streptomycin solution under a humidified atmosphere of 5% CO_2 and 95% air at 37 °C. 90 μL culture solution (containing fetal bovine serum) and 100 μL of cell suspension at a density of 2 × 10^3 cell/mL was plated in 96-well microtiter plates, allowed to attach overnight, and then exposed to 10 μL varying concentrations (0.032−100 μM) of compounds for 72 h. The CTG solution (100 μL) was then added to each well and incubated for 10 min. Absorbance was then determined on a Spectra Max Plus plate reader at 500 nm. Adriamycin was used as the positive control.

Figure S1. LC-MS profile for the synthesis of **1**–**4** from **5** and **6** under basic condition

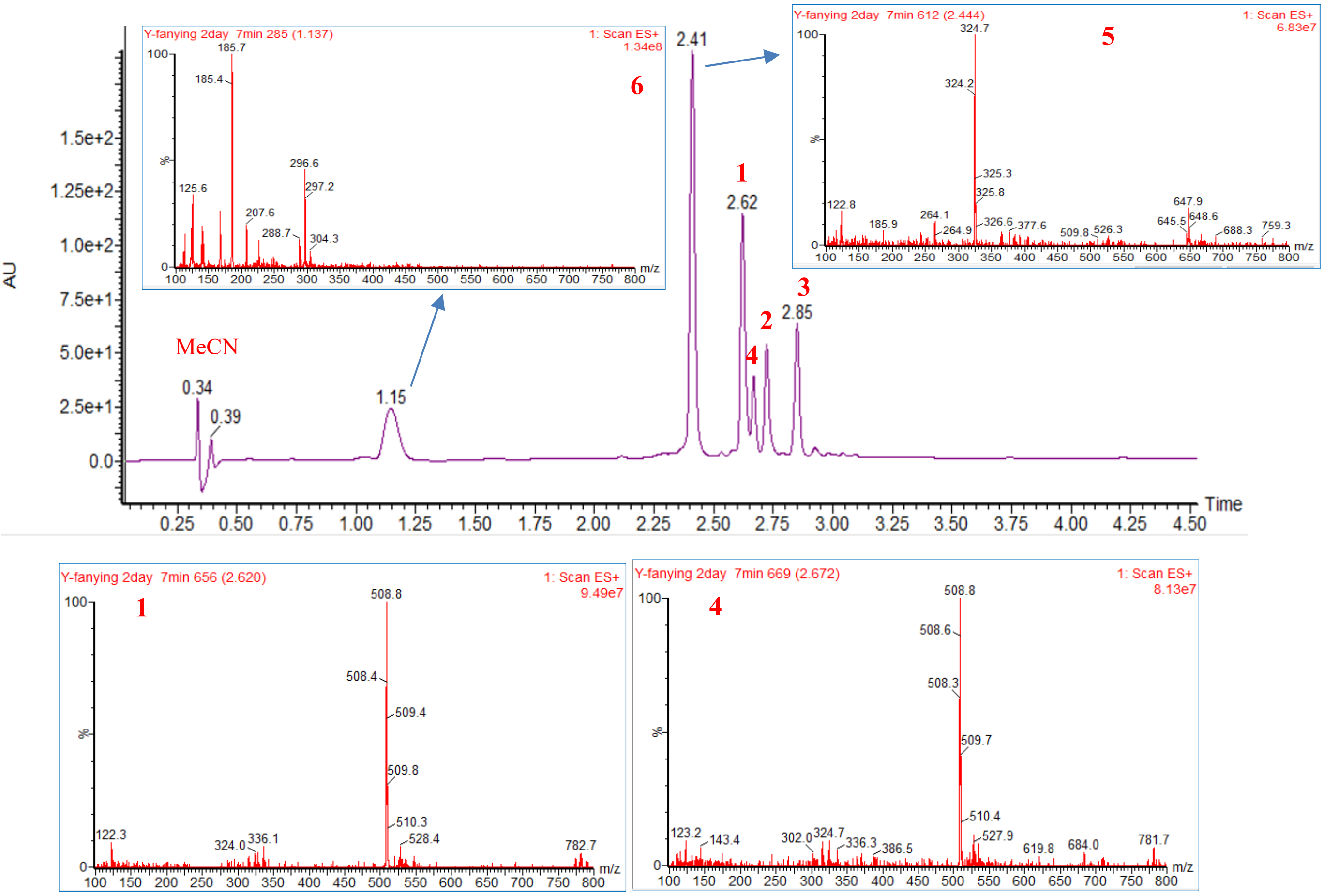

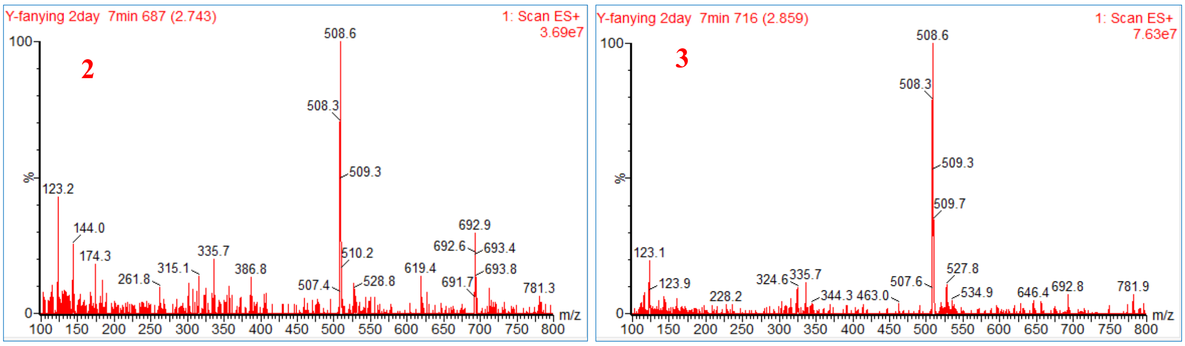

Figure S2. LC-MS profile of the reaction mixture of **5** and **6** under neutral condition

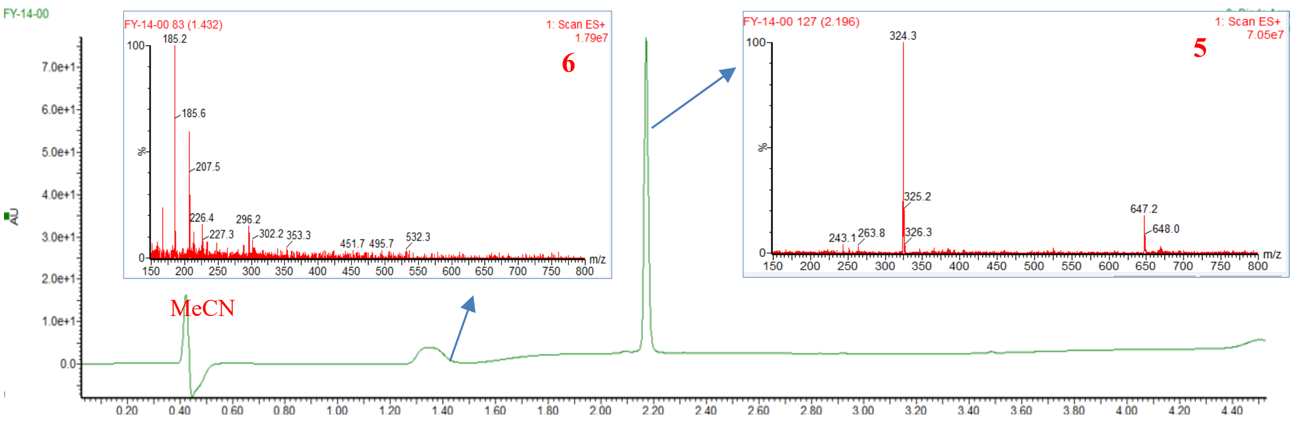

Figure S3. LC-MS profile of the reaction mixture of **5** and **6** under acidic condition.

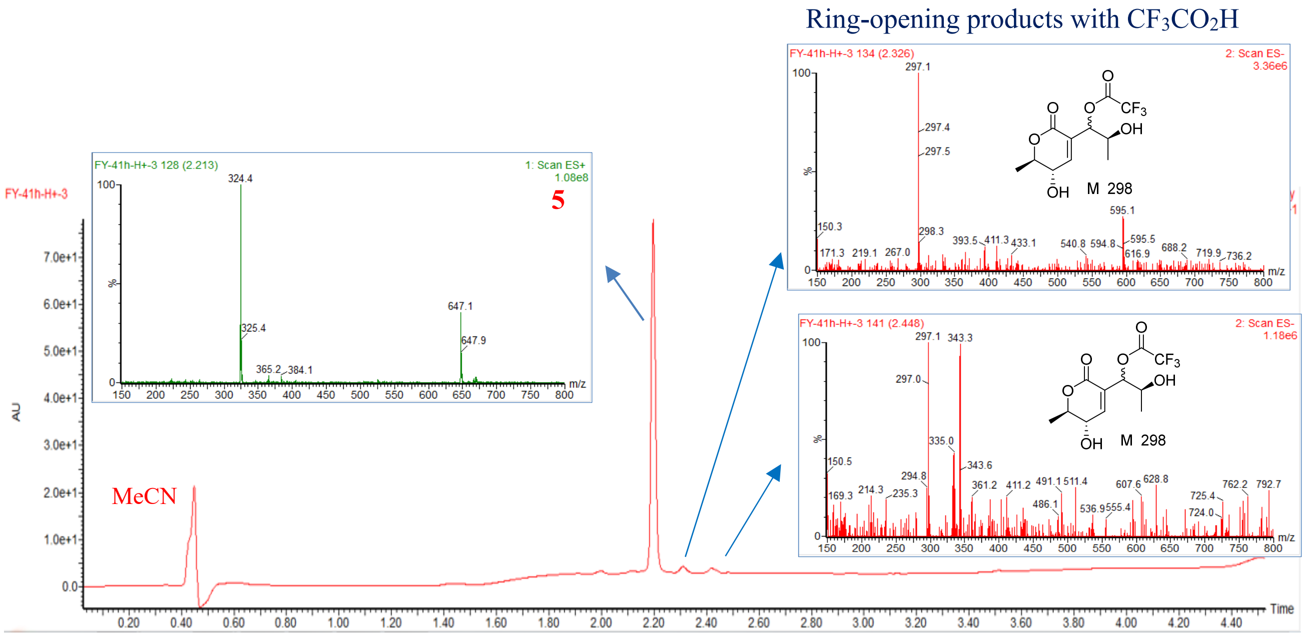

Figure S4. HRESIMS spectrum of ochrazepine A (**1**)

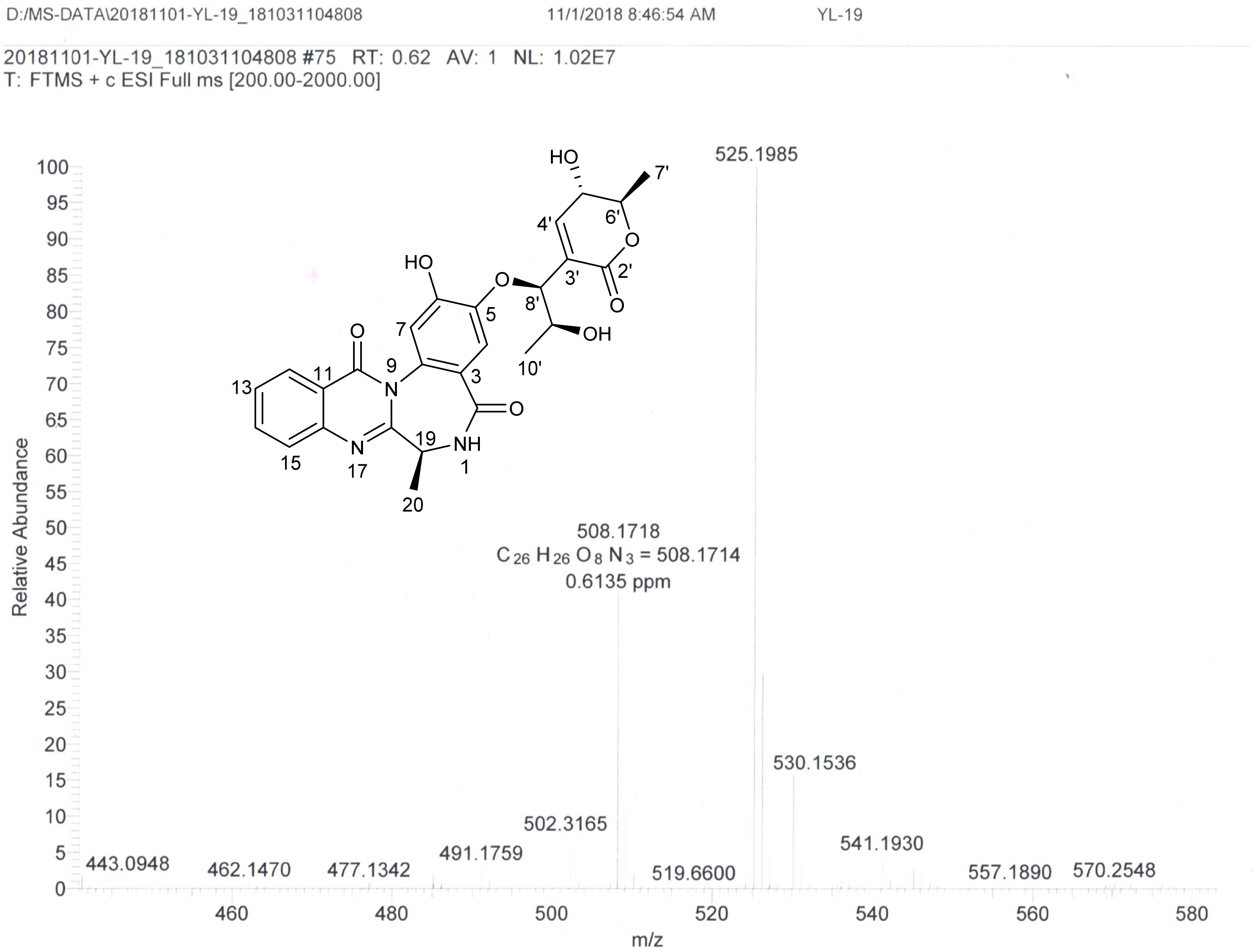

Figures S5. ^{1}H-NMR spectrum of ochrazepine A (**1**) in DMSO-d_6 (1)

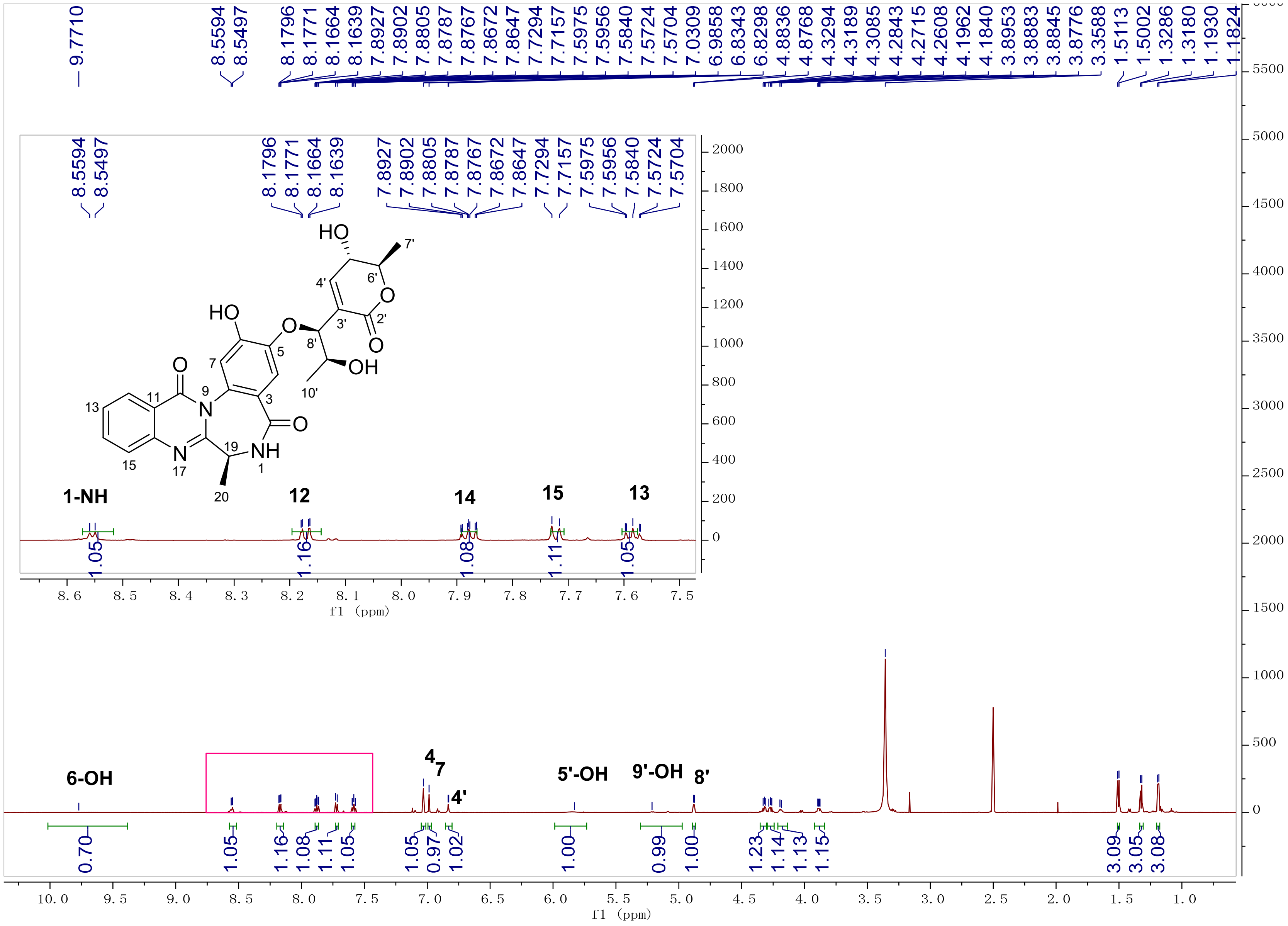

Figure S6. ^{1}H-NMR spectrum of ochrazepine A (**1**) in DMSO-d_6 (2)

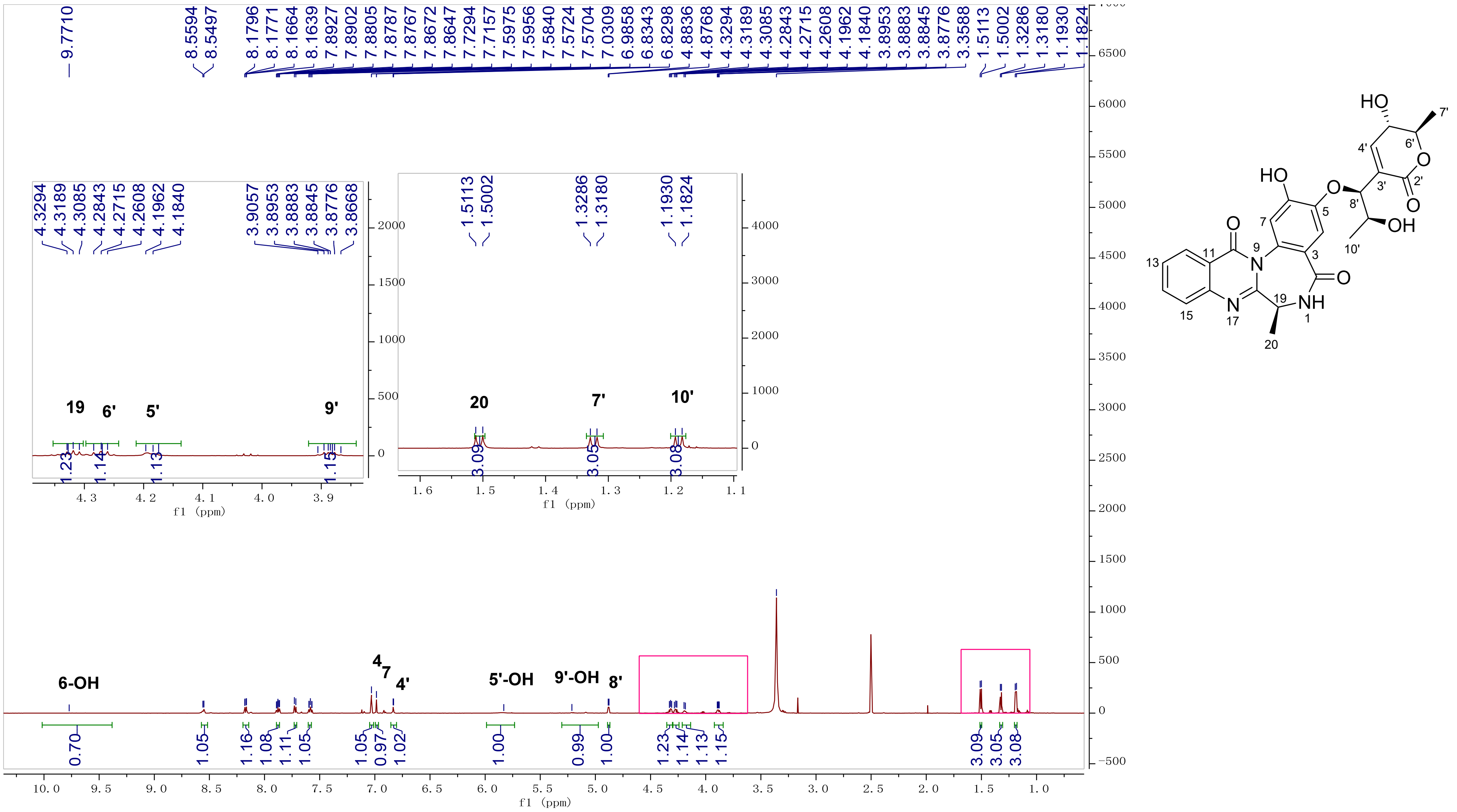

Figure S7. ^{13}C-NMR spectrum of ochrazepine A (**1**) in DMSO-d_6

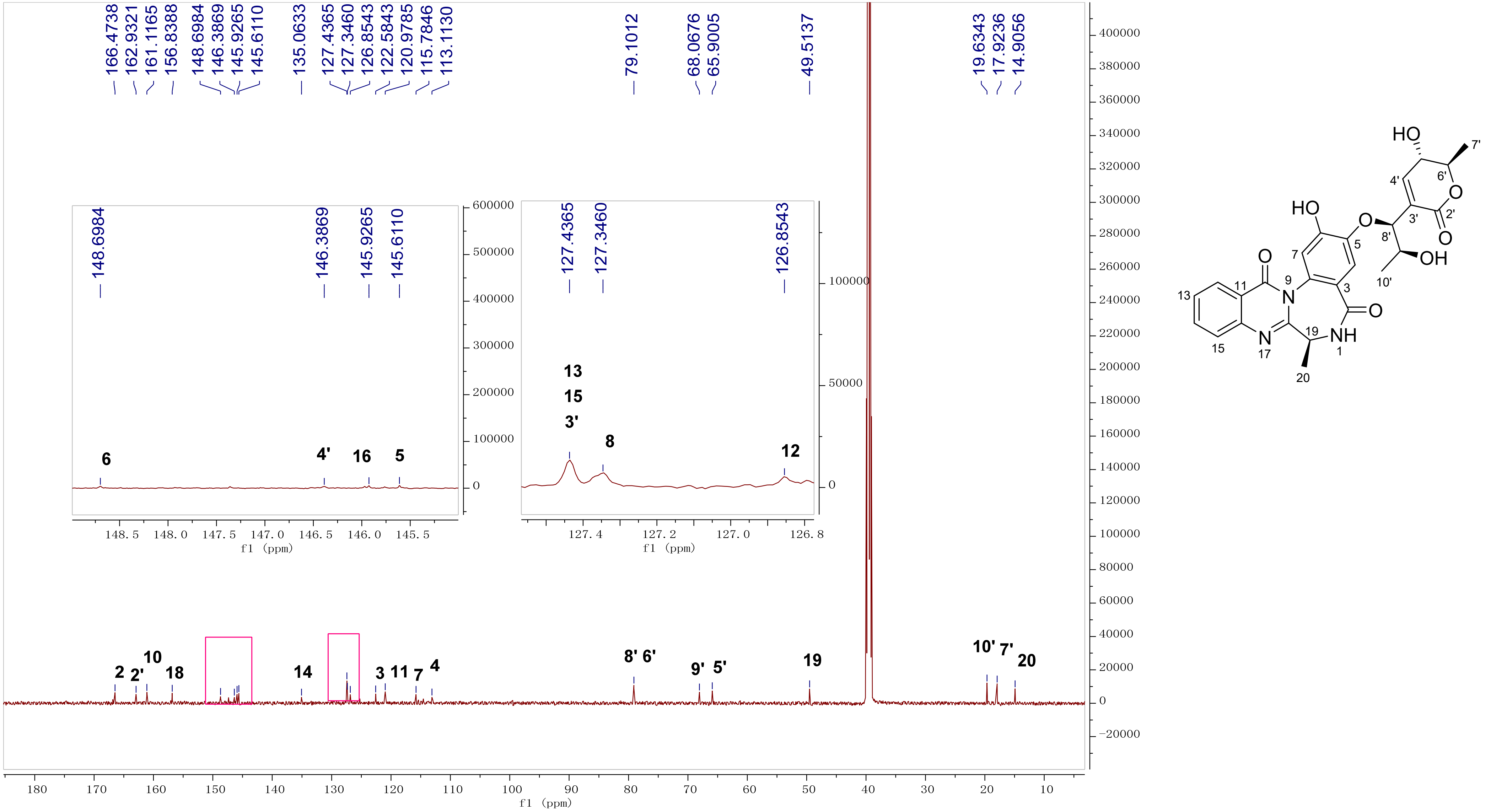

Figure S8. HMQC spectrum of ochrazepine A (**1**) in DMSO-d_6 (1)

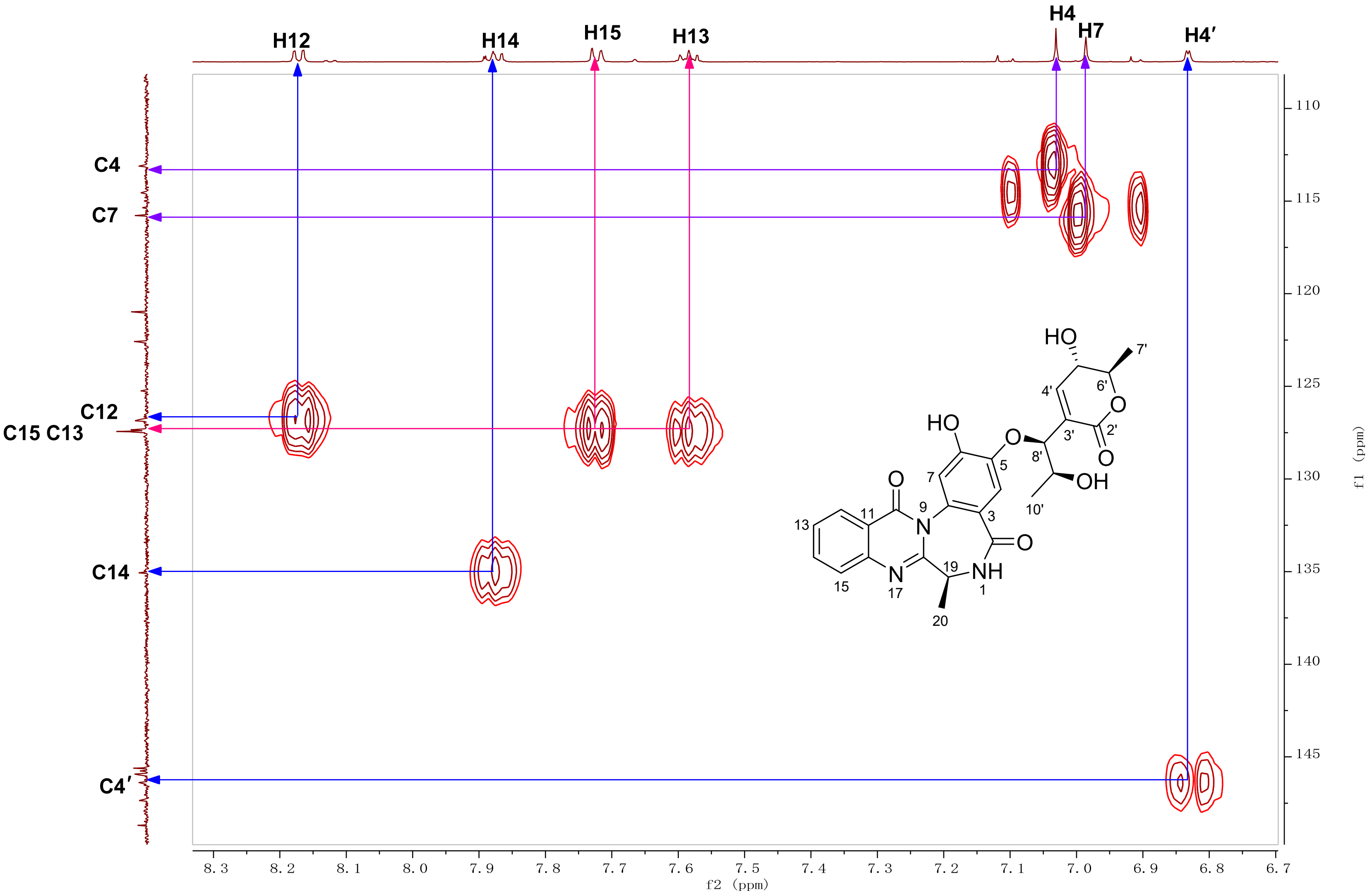

Figure S9. HMQC spectrum of ochrazepine A (**1**) in DMSO-d_6 (2)

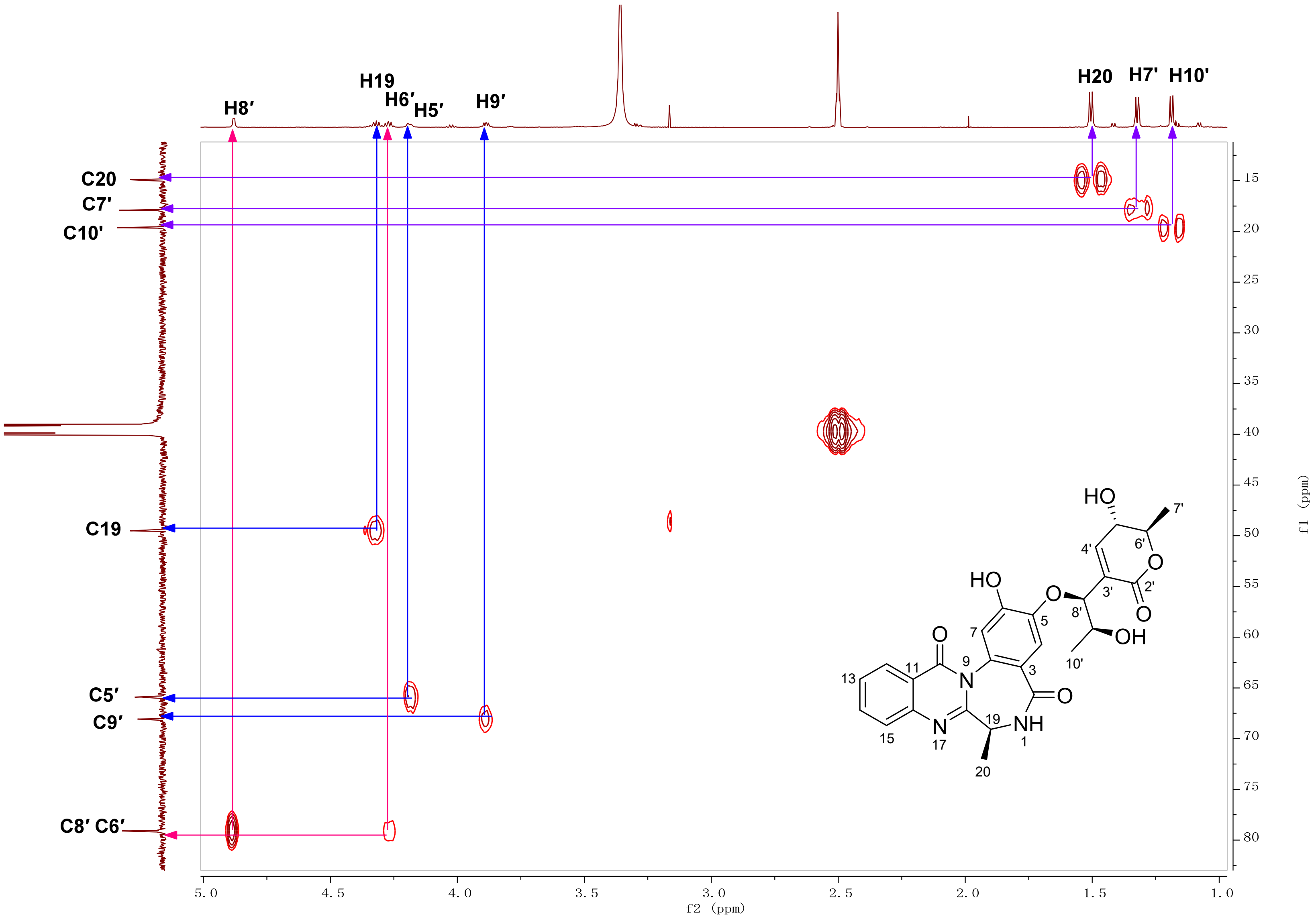

Figure S10. ^{1}H-^{1}H COSY spectrum of ochrazepine A (**1**) in DMSO-d_6 (1)

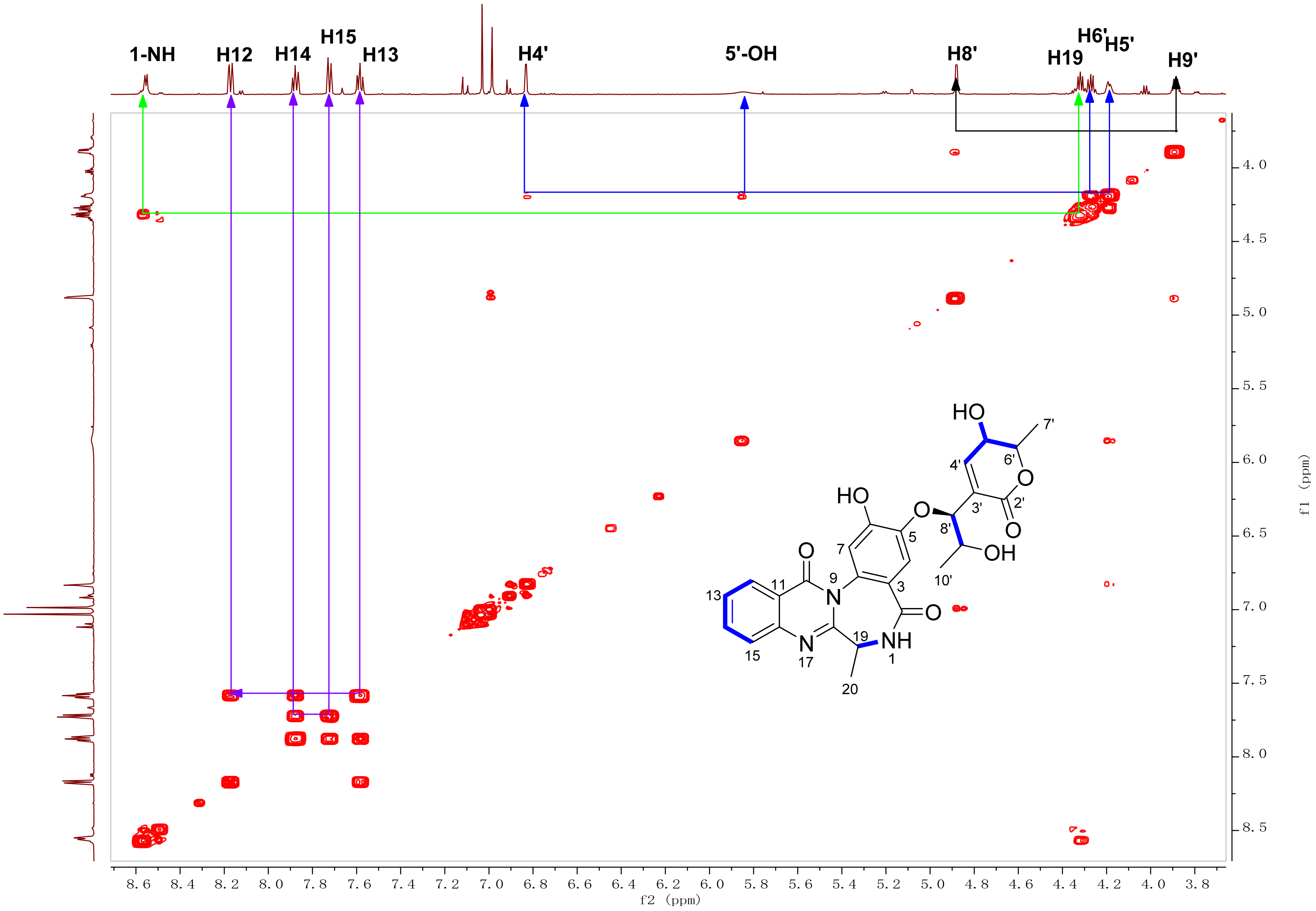

Figure S11. ^{1}H-^{1}H COSY spectrum of ochrazepine A (**1**) in DMSO-d_6 (2)

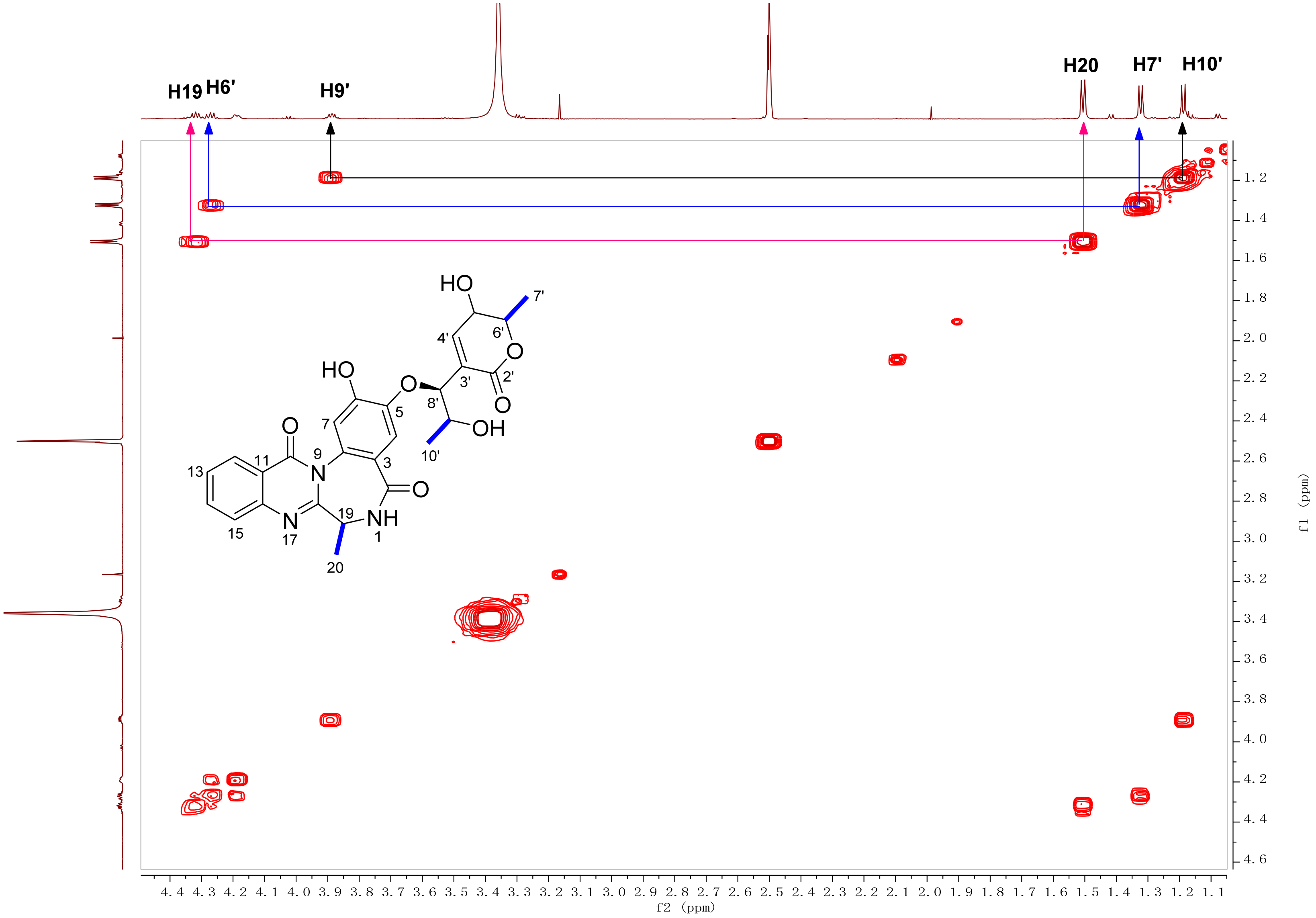

Figure S12. HMBC spectrum of ochrazepine A (**1**) in DMSO-d_6 (1)

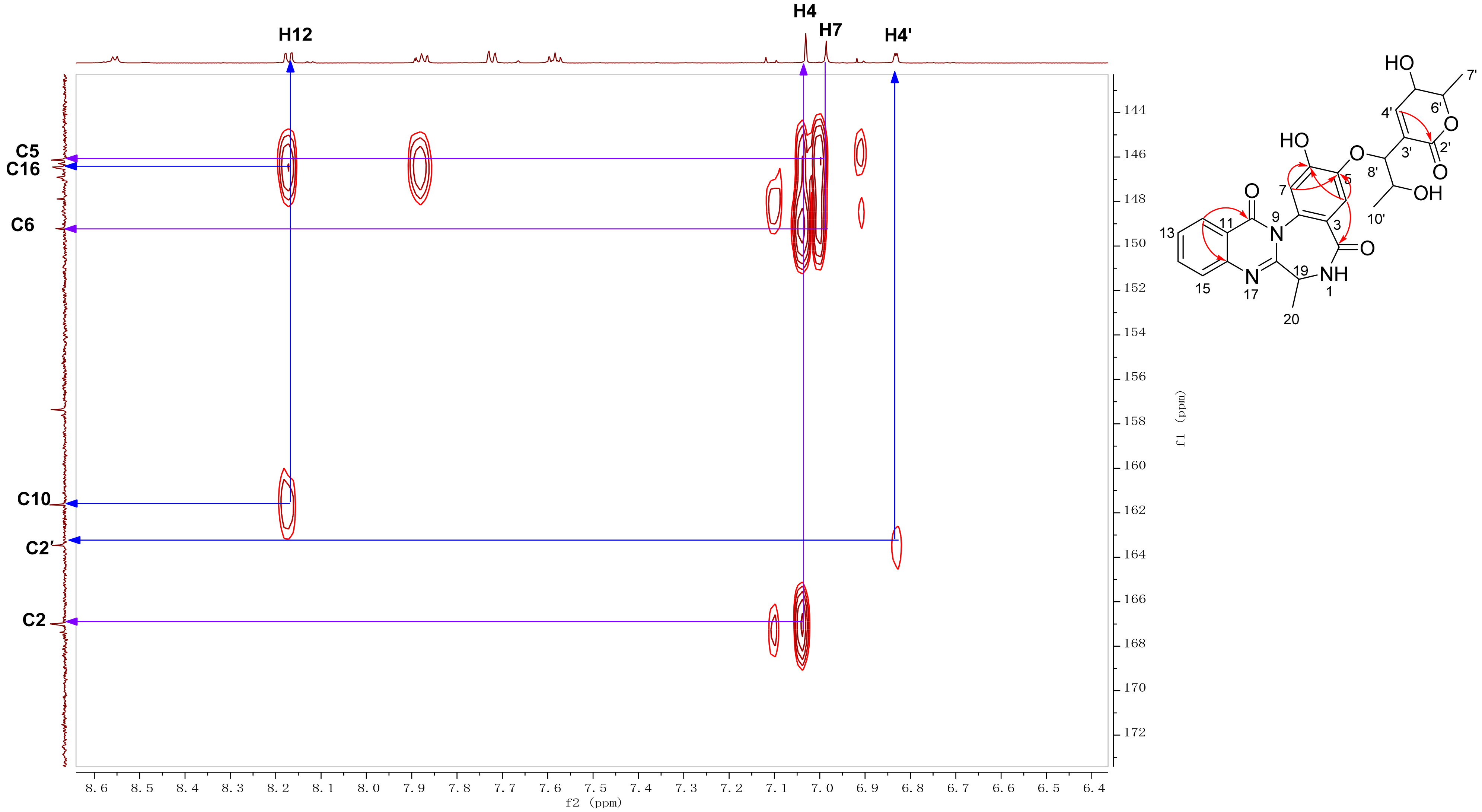

Figure S13. HMBC spectrum of ochrazepine A (**1**) in DMSO-d_6 (2)

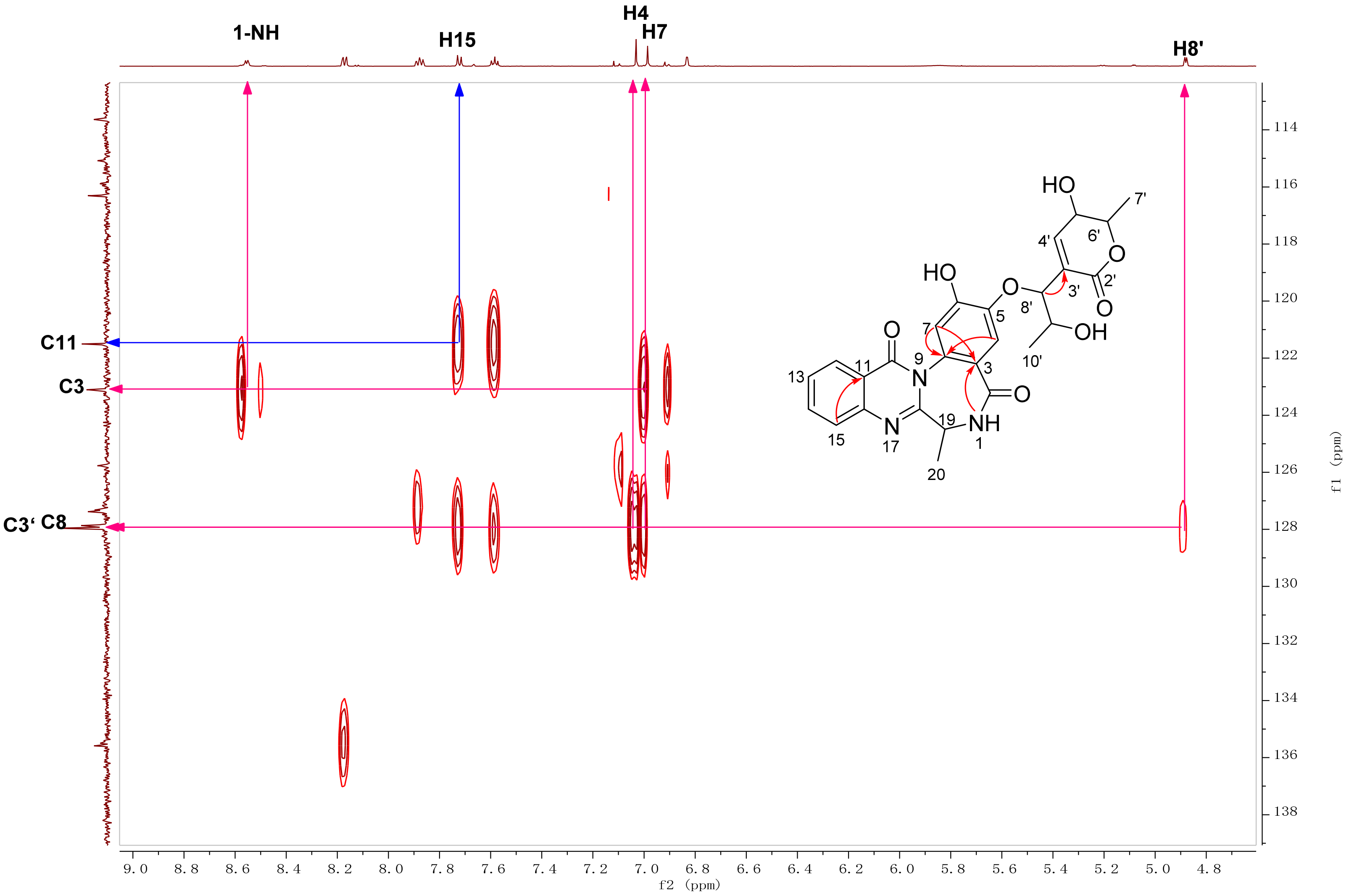

Figure S14. HMBC spectrum of ochrazepine A (**1**) in DMSO-d_6 (3)

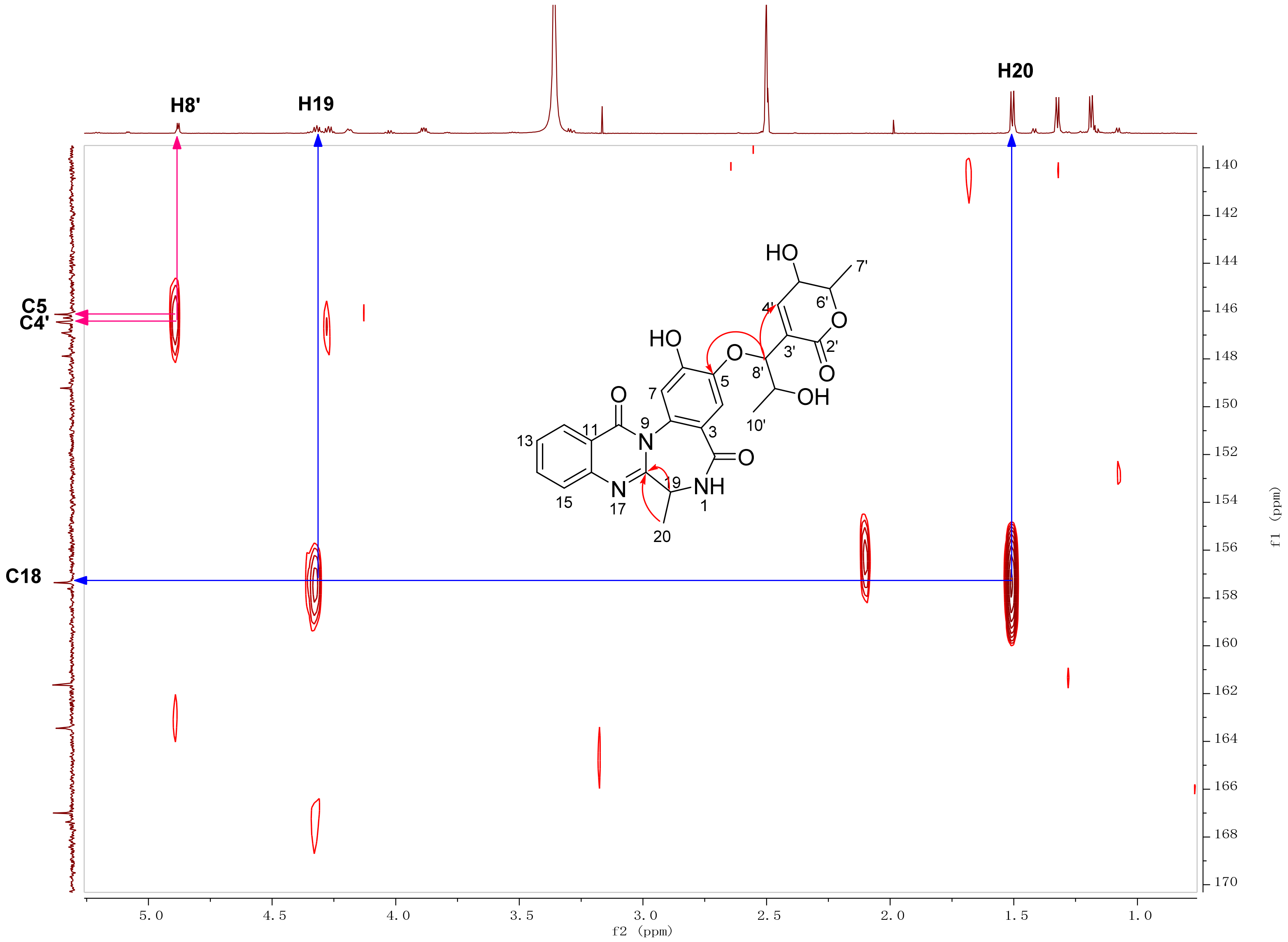

Figure S15. HMBC spectrum of ochrazepine A (**1**) in DMSO-d_6 (4)

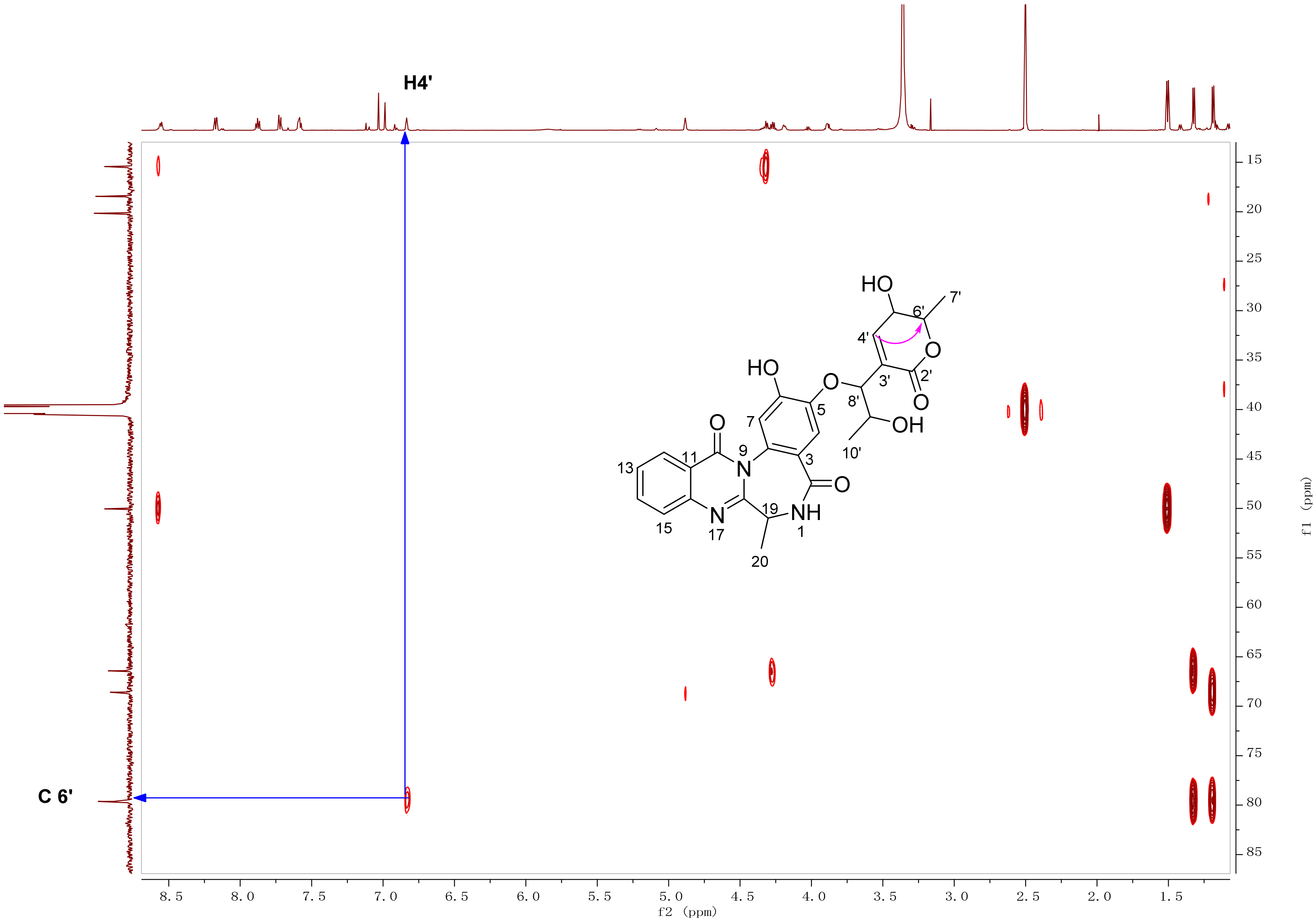

Figure S16. NOESY spectrum of ochrazepine A (**1**) in DMSO-d_6 at 25°C (1)

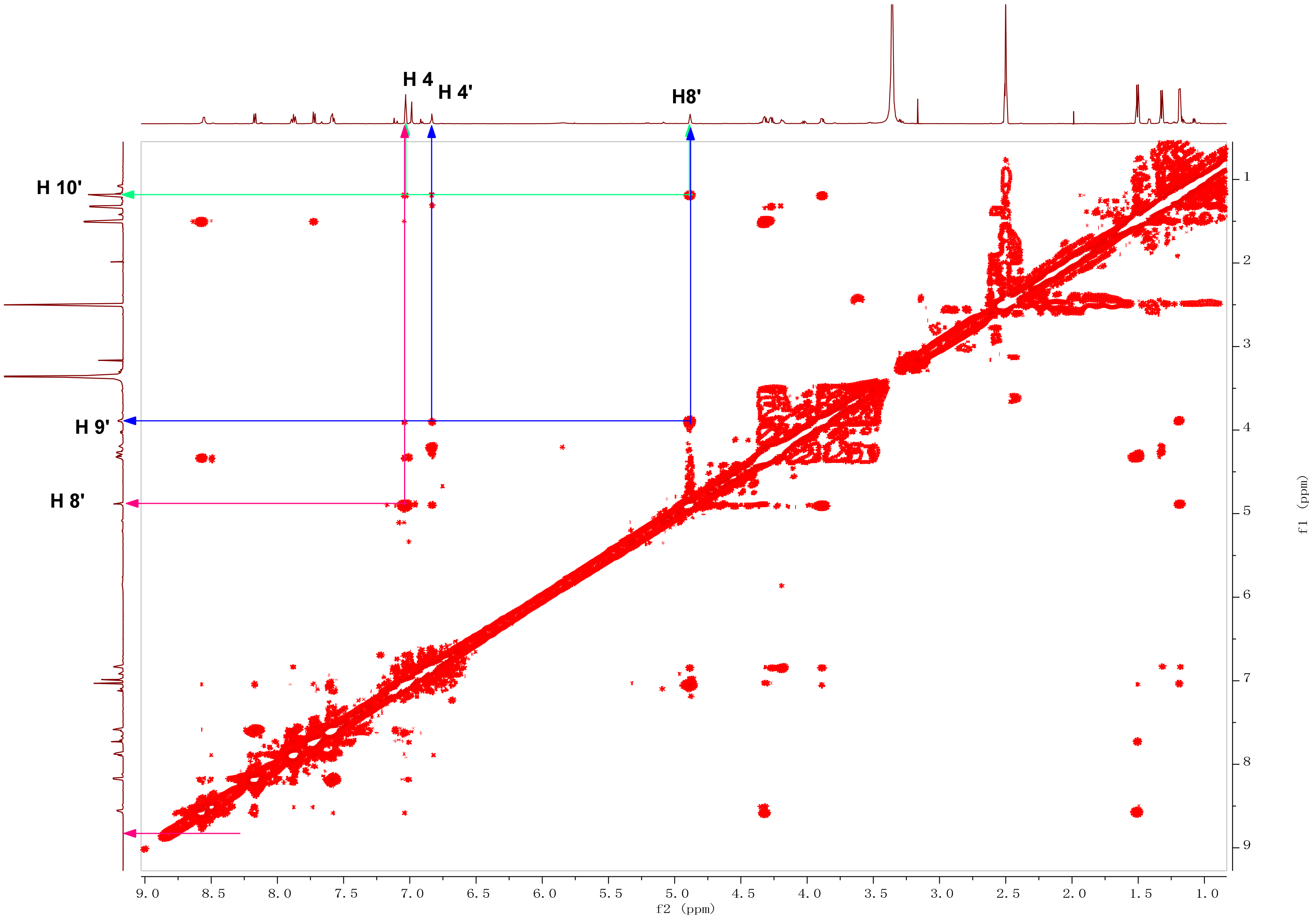

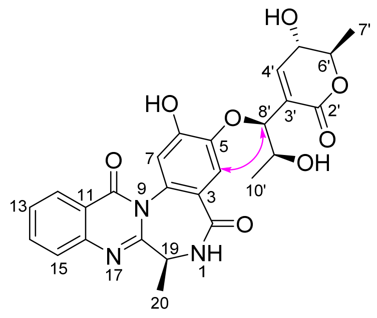

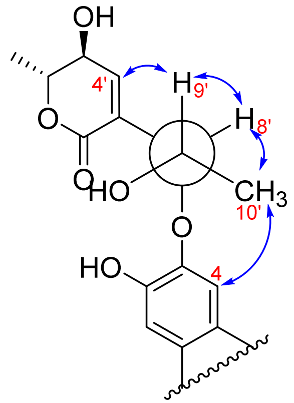

Figure S17. NOESY spectrum of ochrazepine A (**1**) in DMSO-d_6 at 25°C (2)

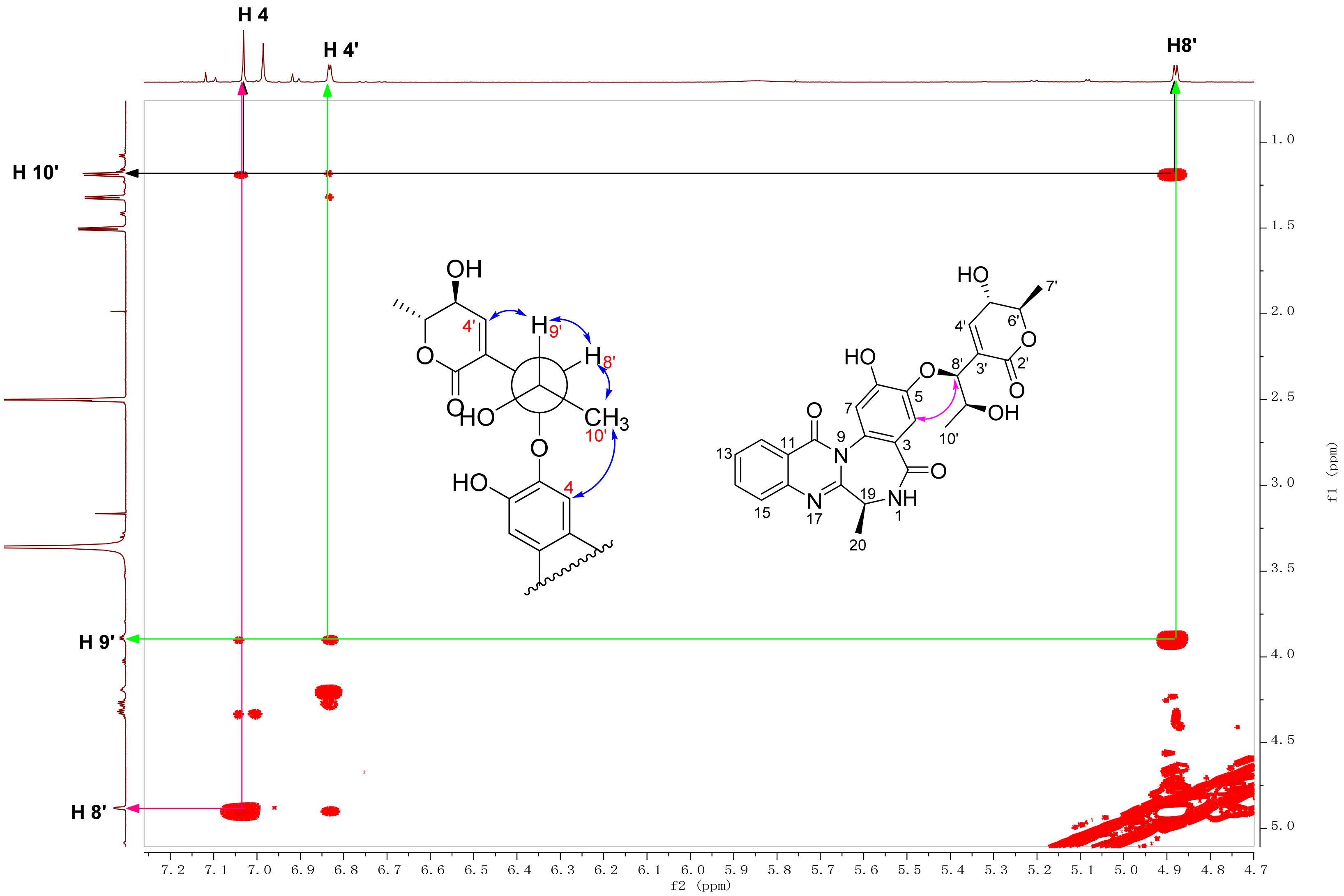

Figure S18. ^{1}H-NMR spectrum of ochrazepine A (**1**) in MeOH-d_4 at 1°C

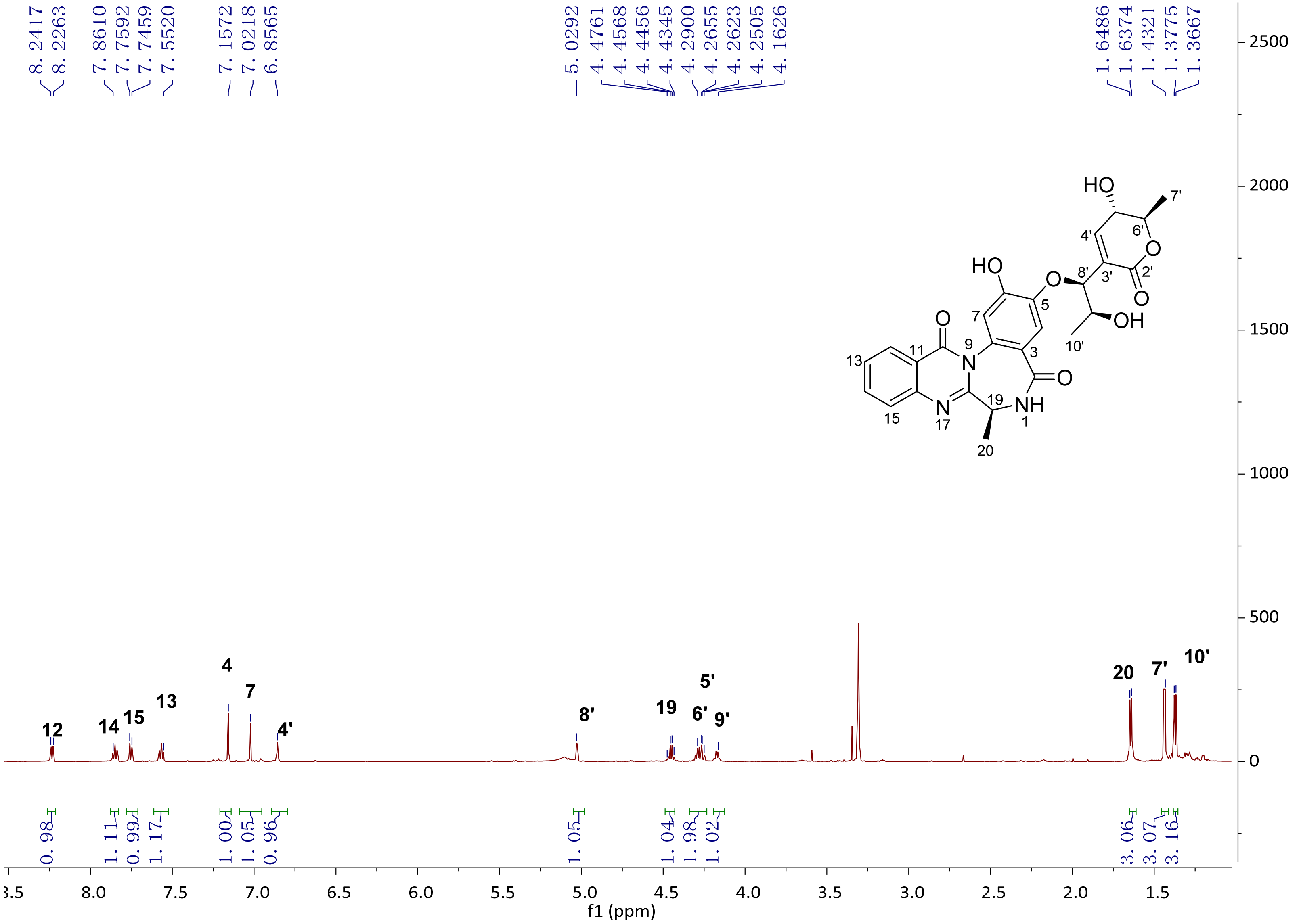

Figures S19. NOESY spectrum of ochrazepine A (**1**) in MeOH-d_4 at 1°C (1)

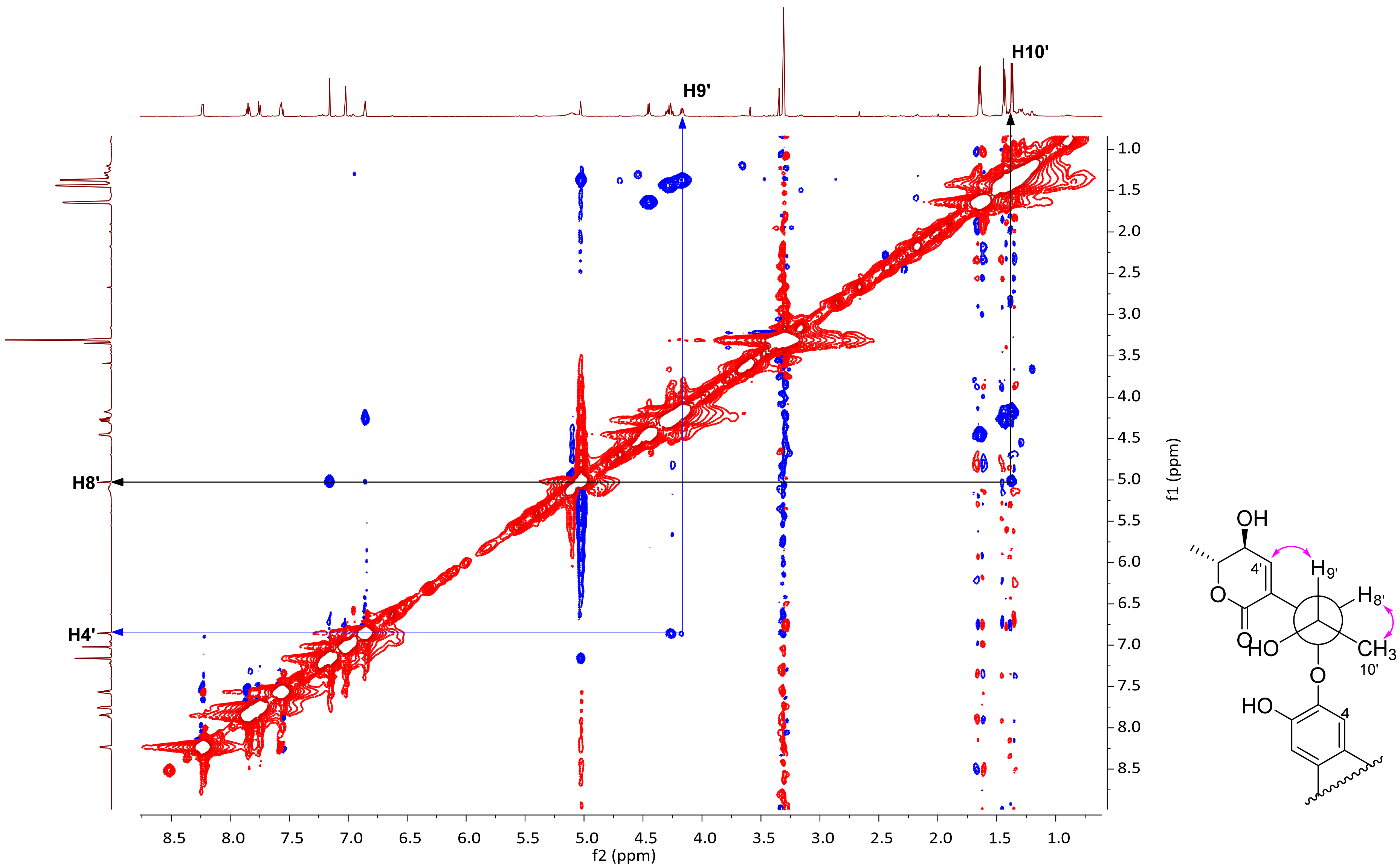

Figure S20. NOESY spectrum of ochrazepine A (**1**) in MeOH-d_4 at 1°C (2)

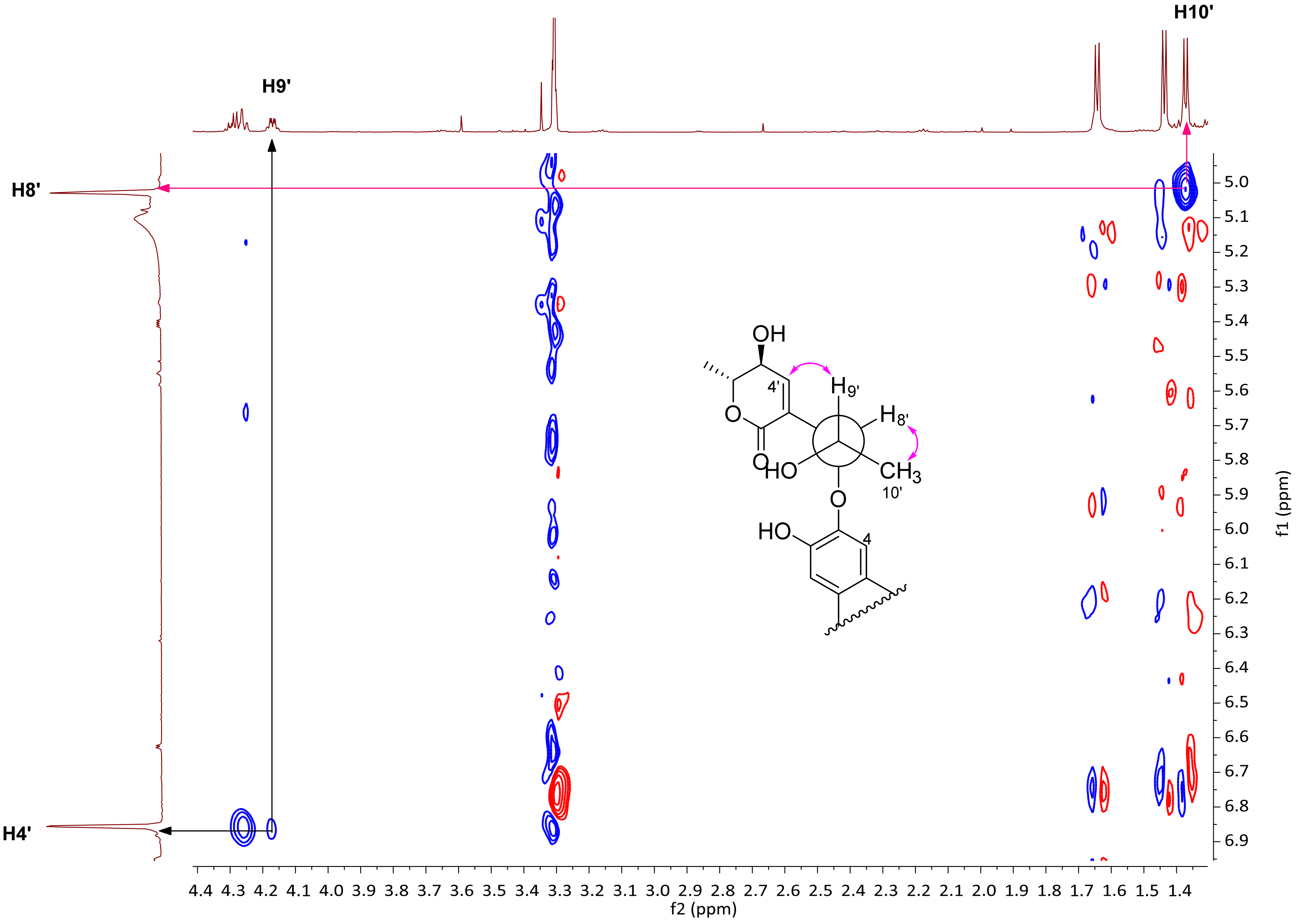

Figure S21. HRESIMS spectrum of ochrazepine B (**2**)

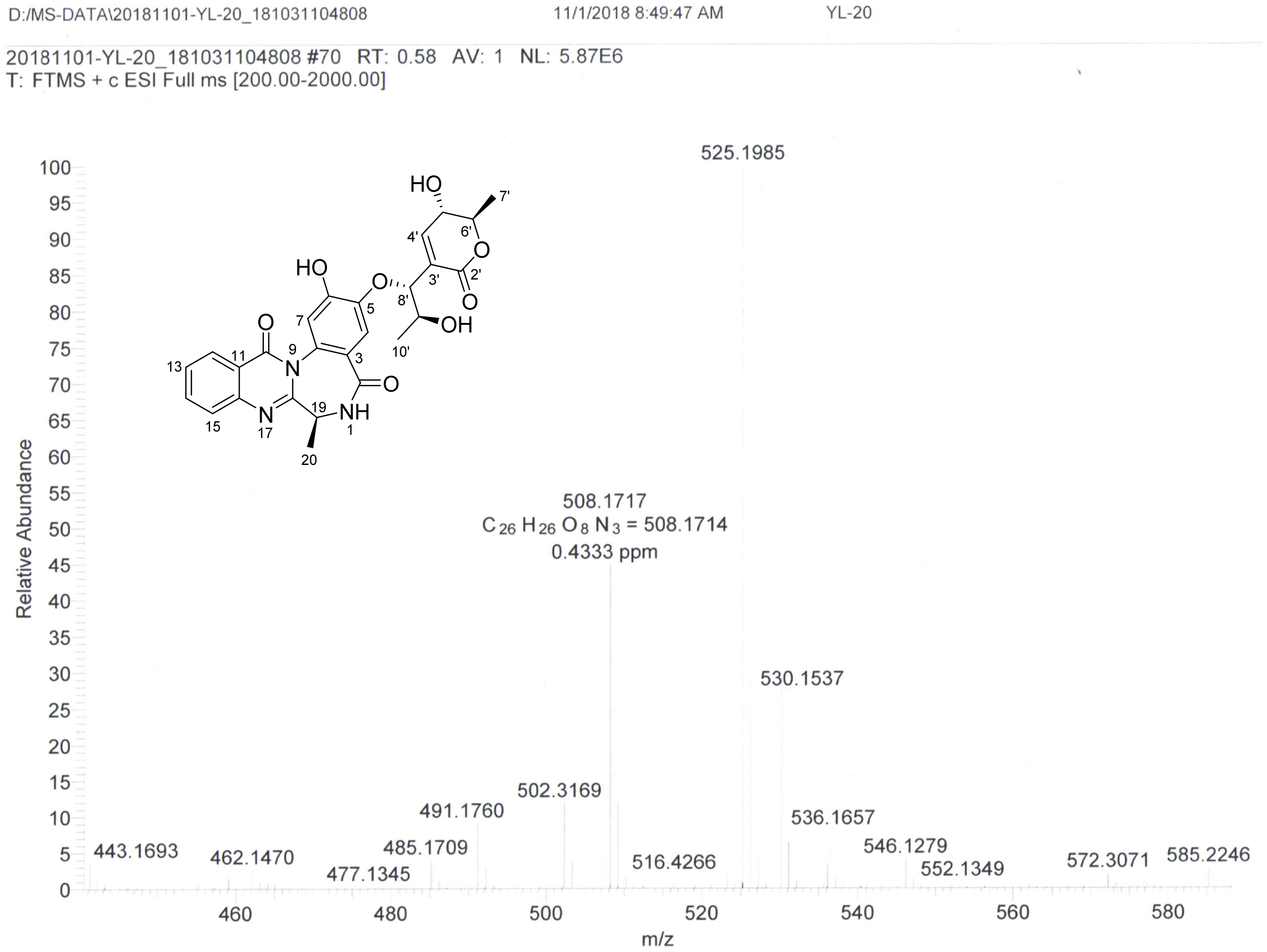

Figure S22. ^{1}H-NMR spectrum of ochrazepine B (**2**) in DMSO-d_6 (1)

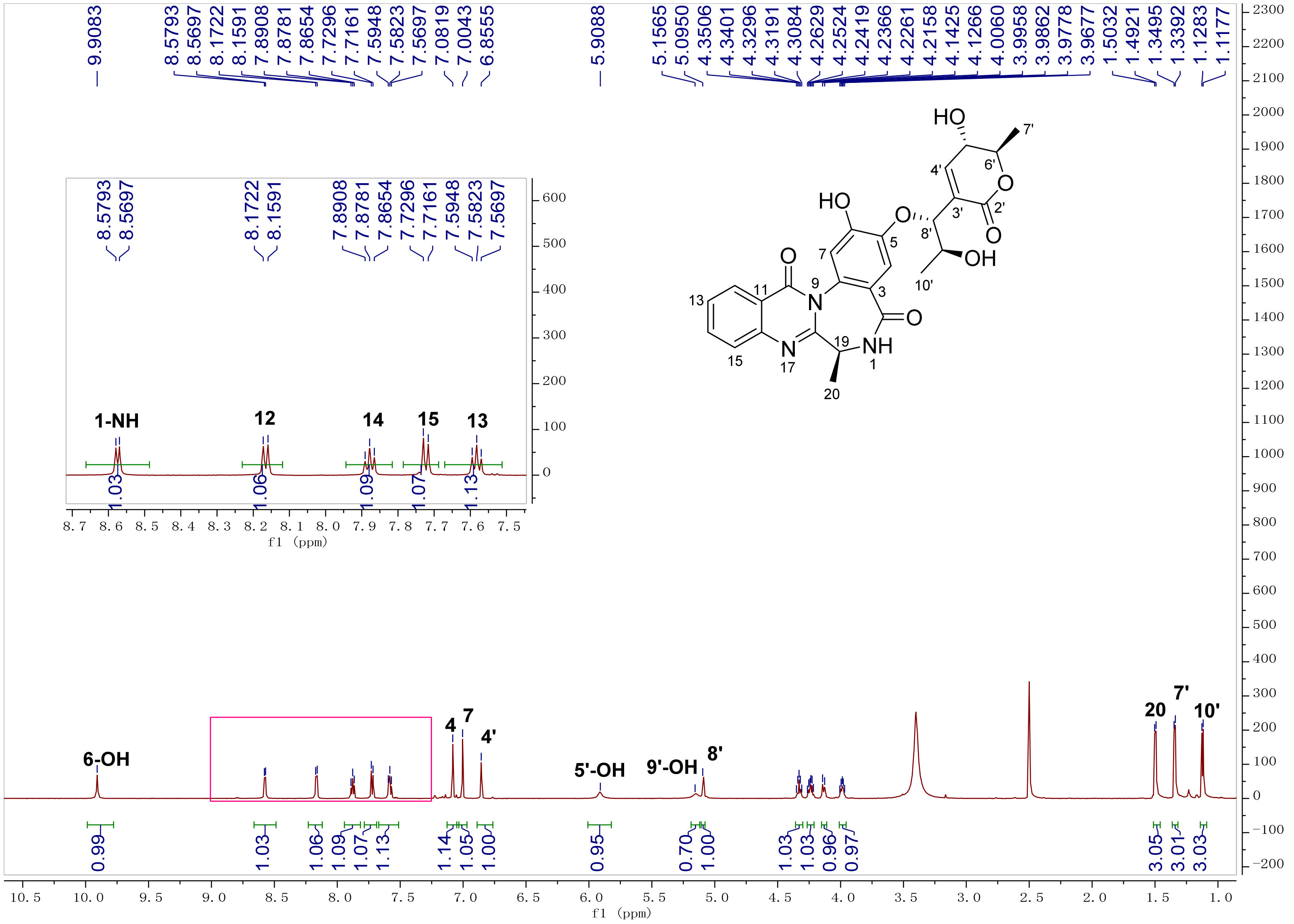

Figure S23. ^{1}H-NMR spectrum of ochrazepine B (**2**) in DMSO-d_6 (2)

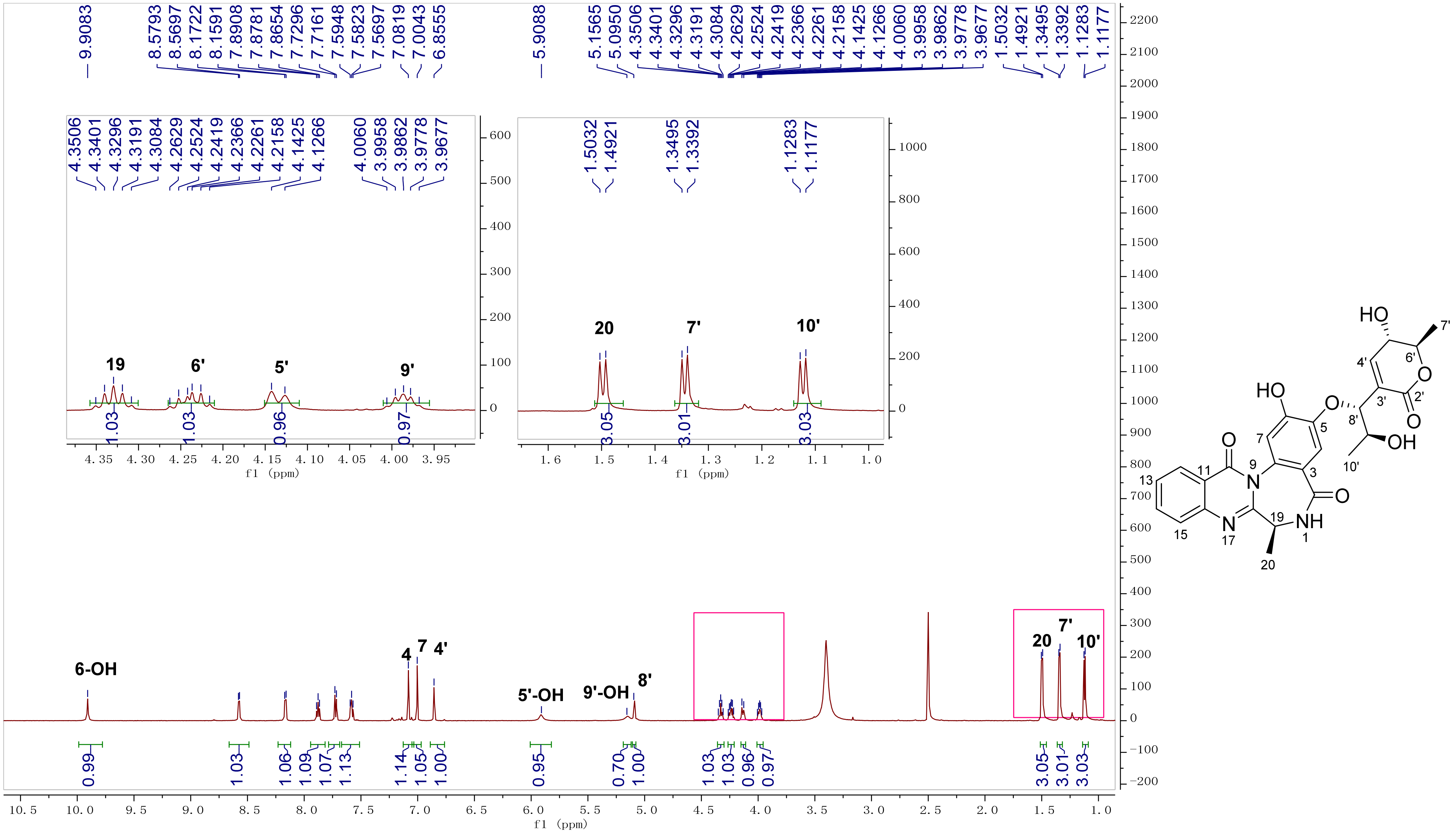

Figure S24. ^{13}C-NMR spectrum of ochrazepine B (**2**) in DMSO-d_6

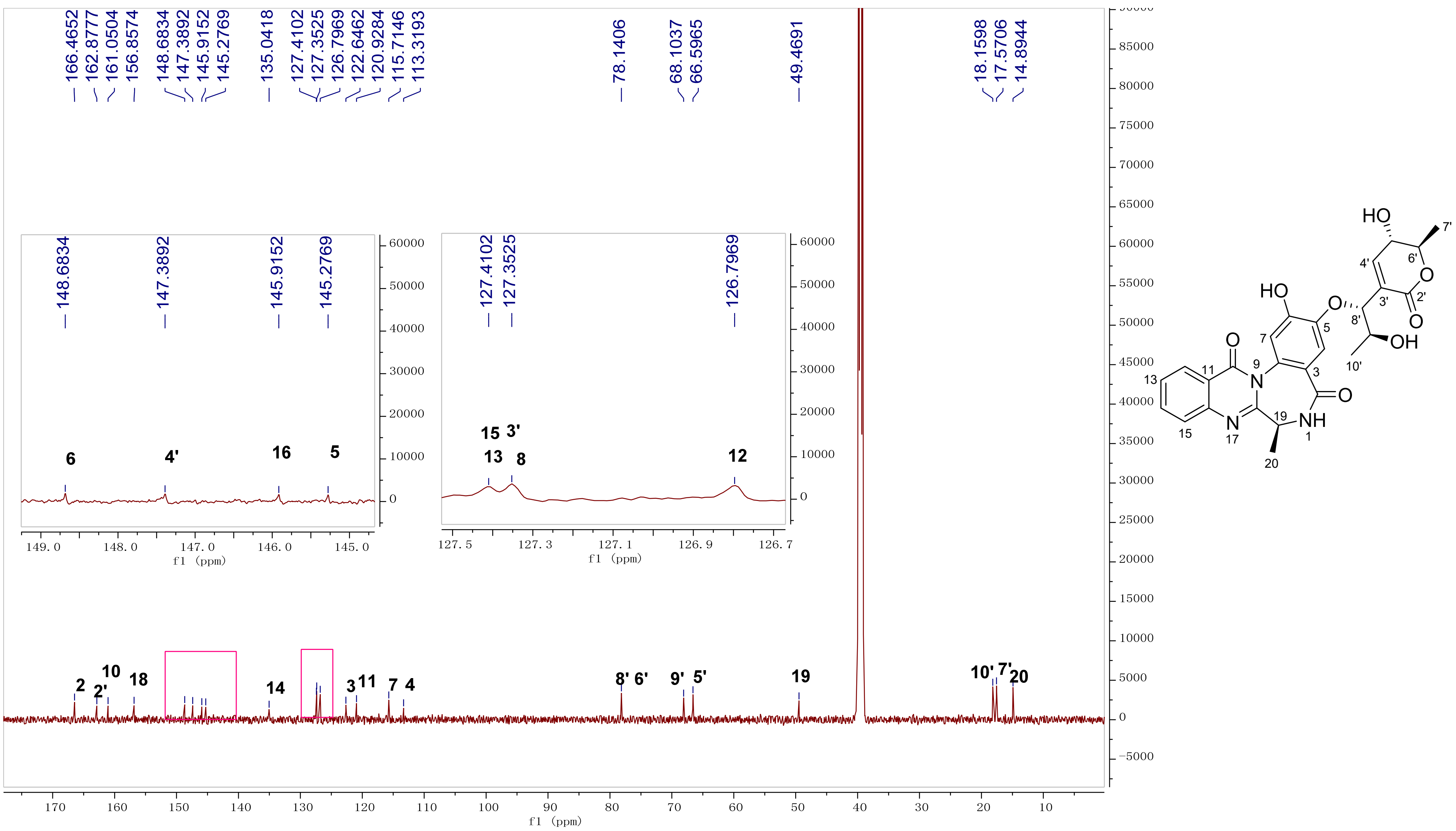

Figure S25. HMQC spectrum of ochrazepine B (**2**) in DMSO-d_6 (1)

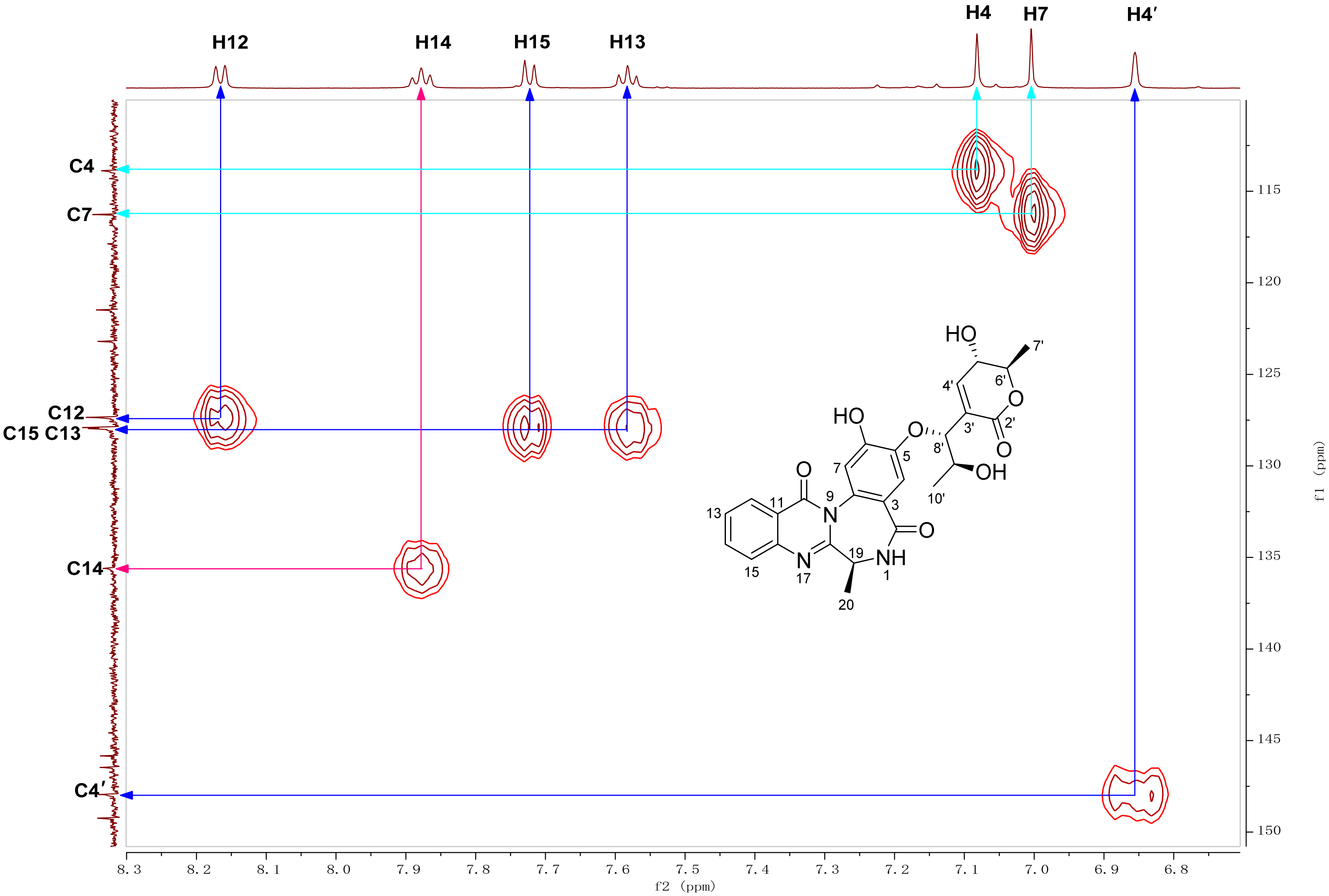

Figure S26. HMQC spectrum of ochrazepine B (**2**) in DMSO-d_6 (2)

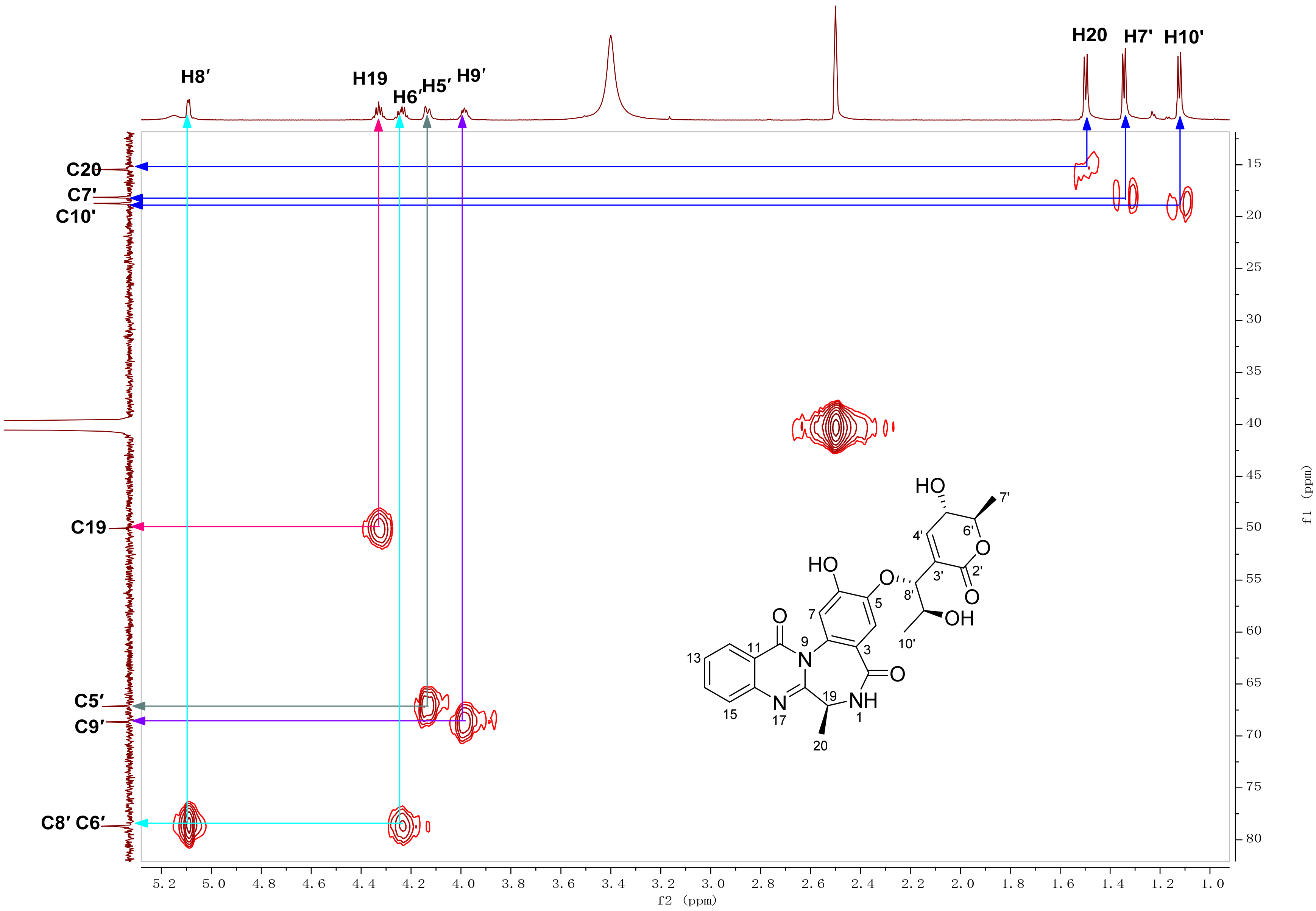

Figure S27. ^{1}H-^{1}H COSY spectrum of ochrazepine B (**2**) in DMSO-d_6 (1)

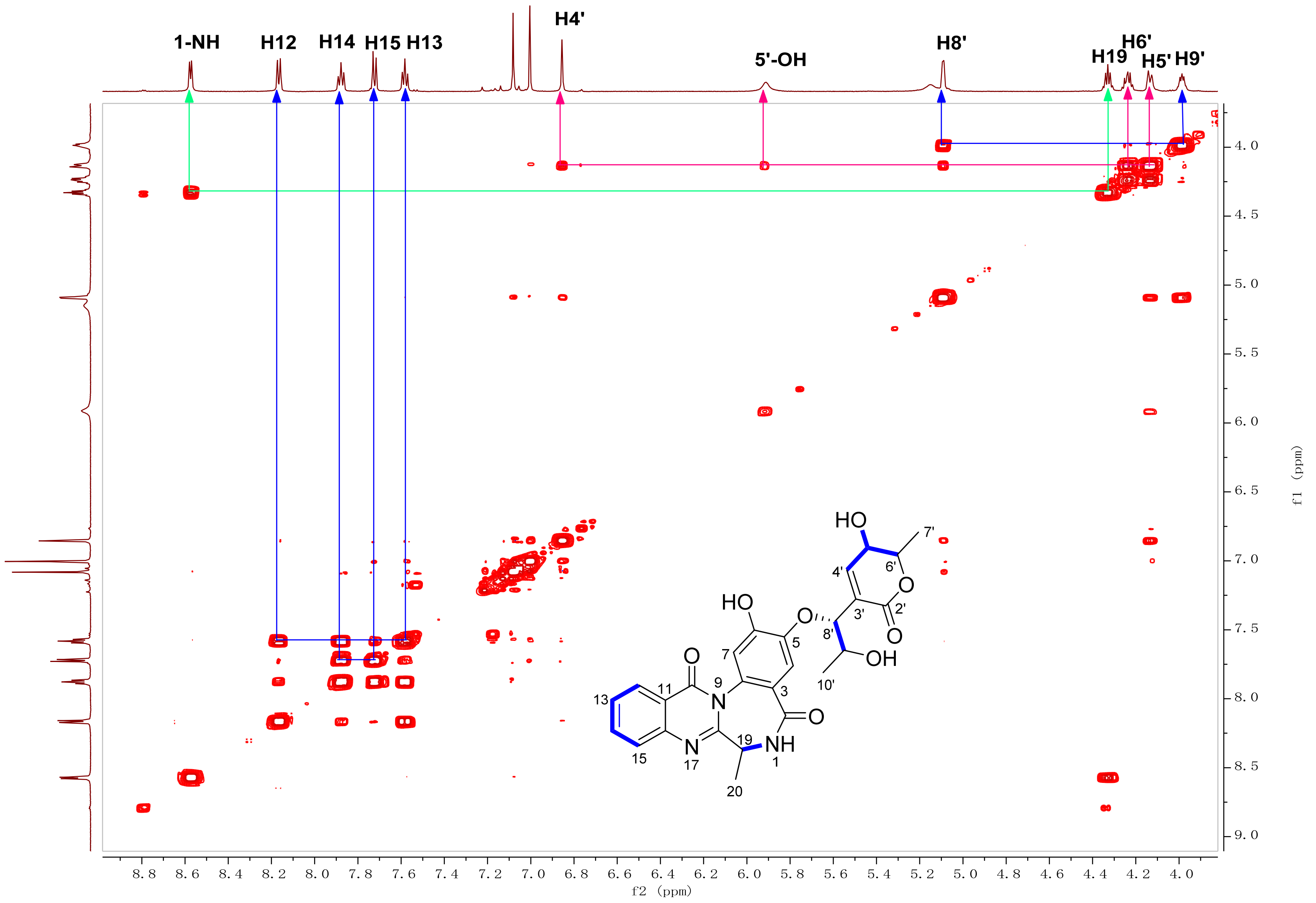

Figure S28. ^{1}H-^{1}H COSY spectrum of ochrazepine B (**2**) in DMSO-d_6 (2)

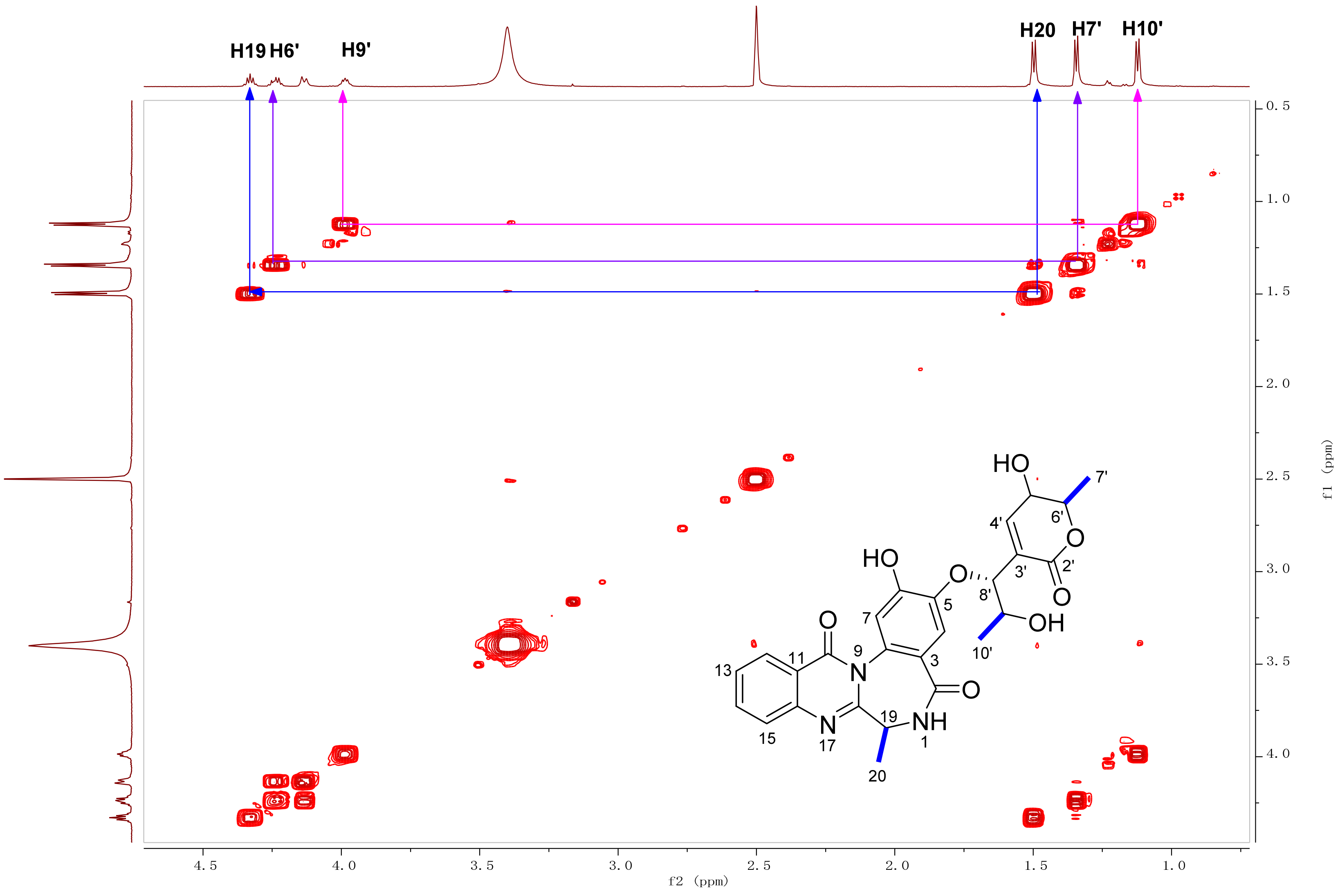

Figure S29. HMBC spectrum of ochrazepine B (**2**) in DMSO-d_6 (1)

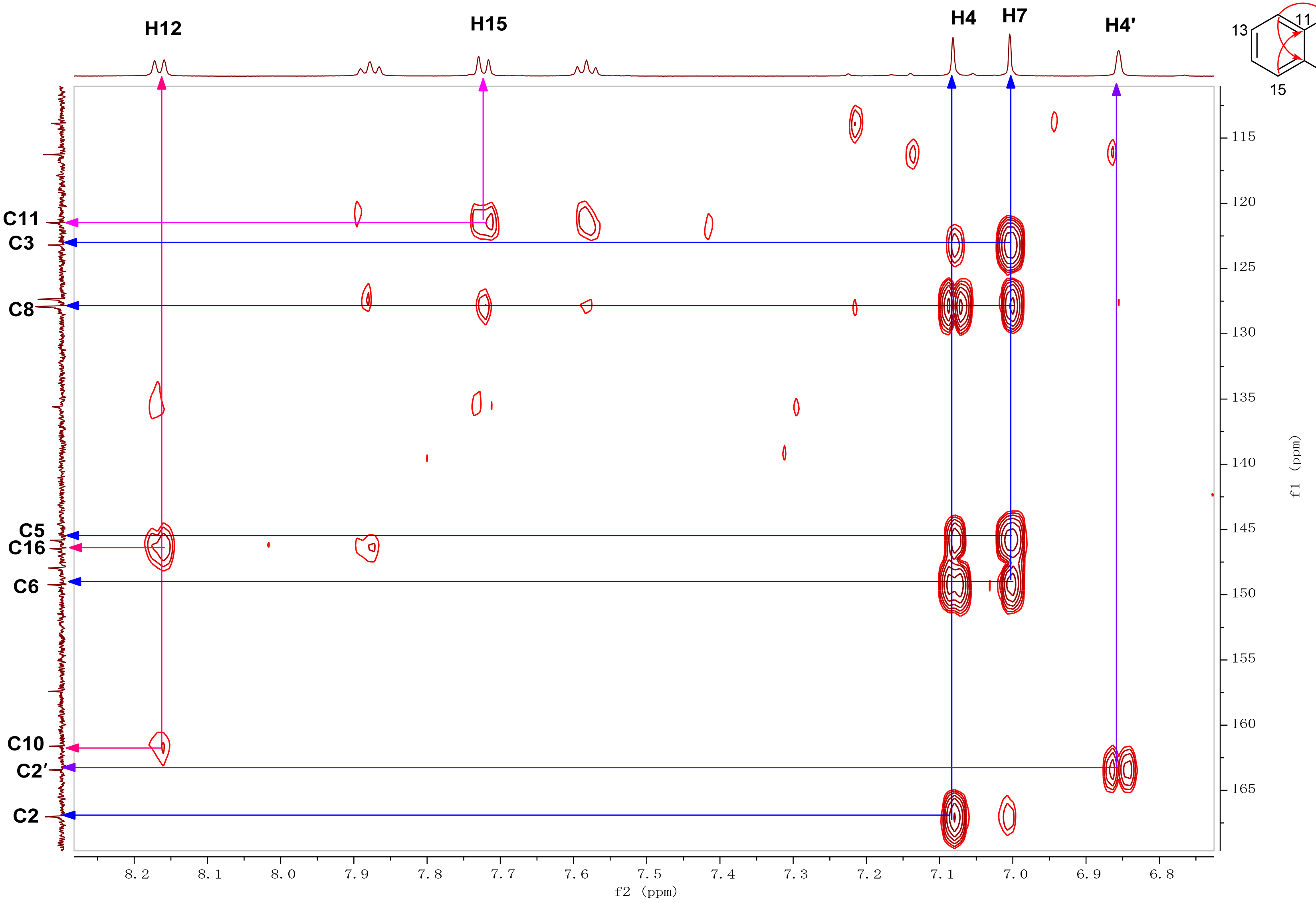

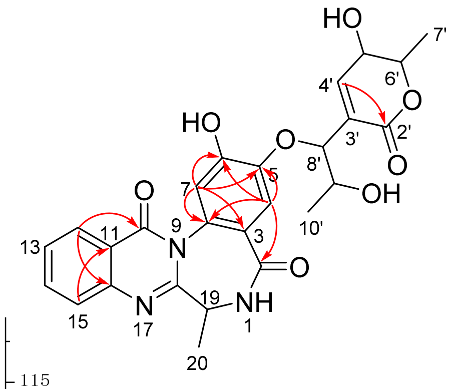

Figure S30. HMBC spectrum of ochrazepine B (**2**) in DMSO-d_6 (2)

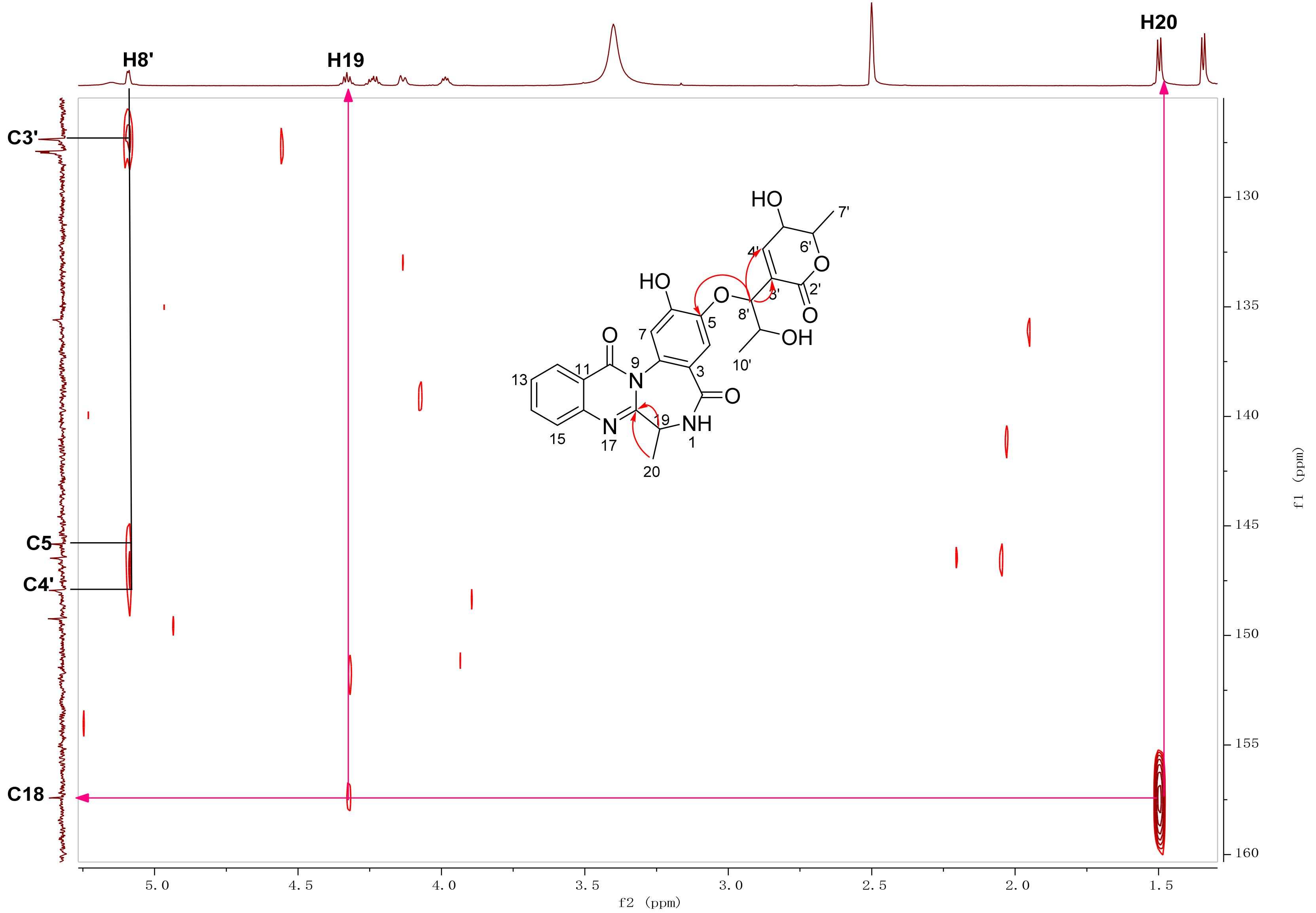

Figure S31. HMBC spectrum of ochrazepine B (**2**) in DMSO-d_6 (3)

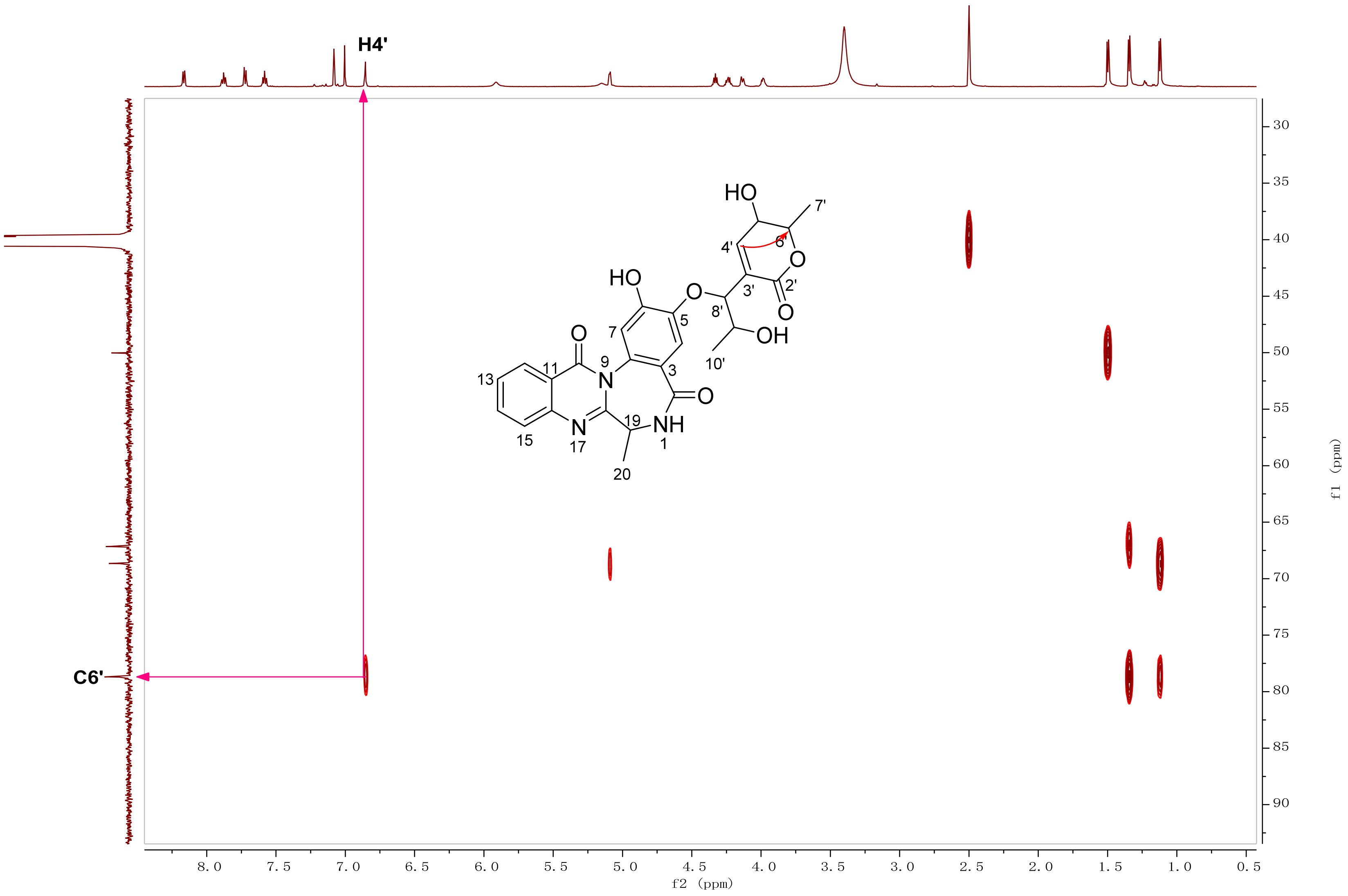

Figure S32. NOESY spectrum of ochrazepine B (**2**) in DMSO-d_6 at 25°C (1)

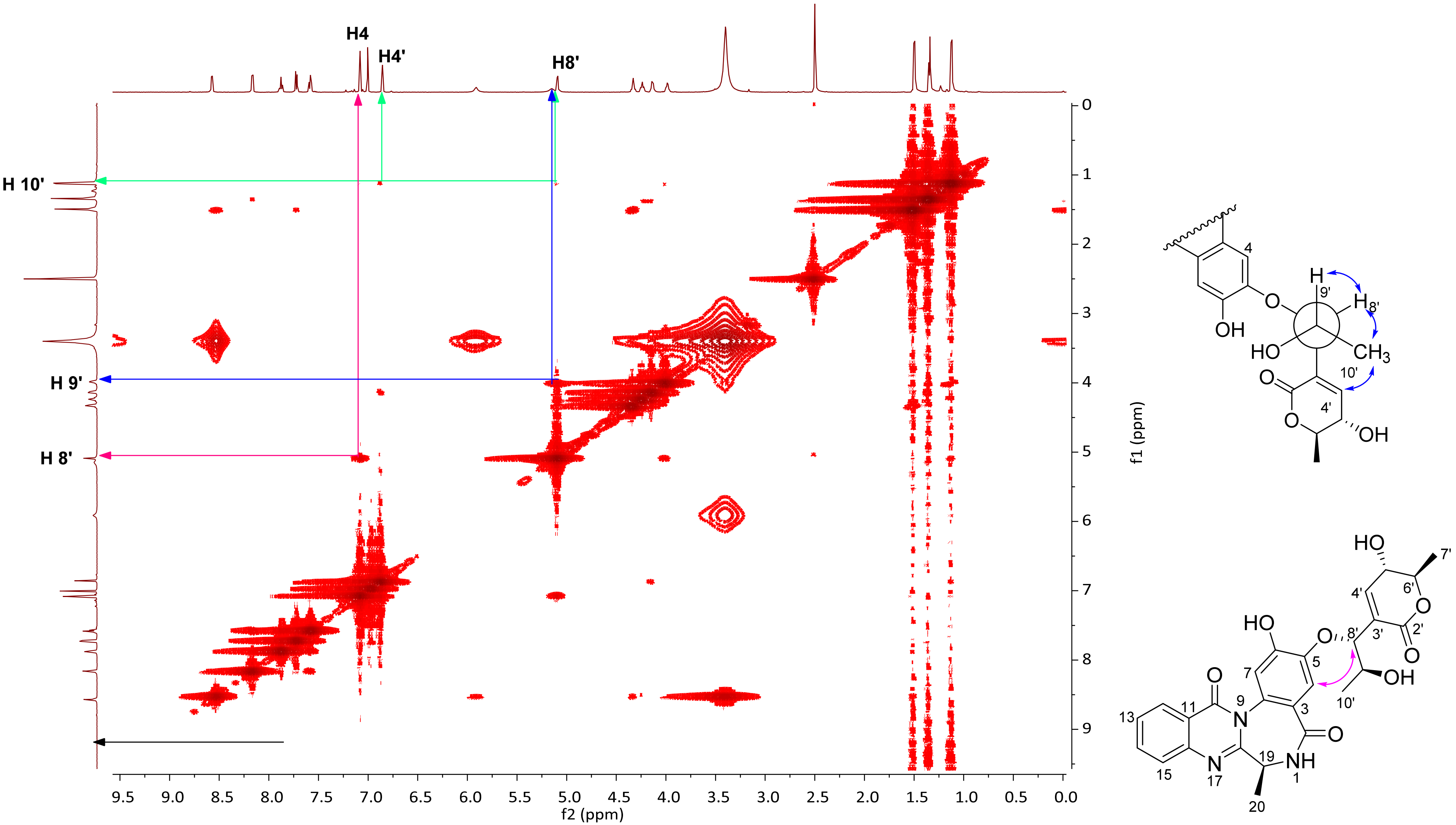

Figure S33. NOESY spectrum of ochrazepine B (**2**) in DMSO-d_6 at 25°C (2)

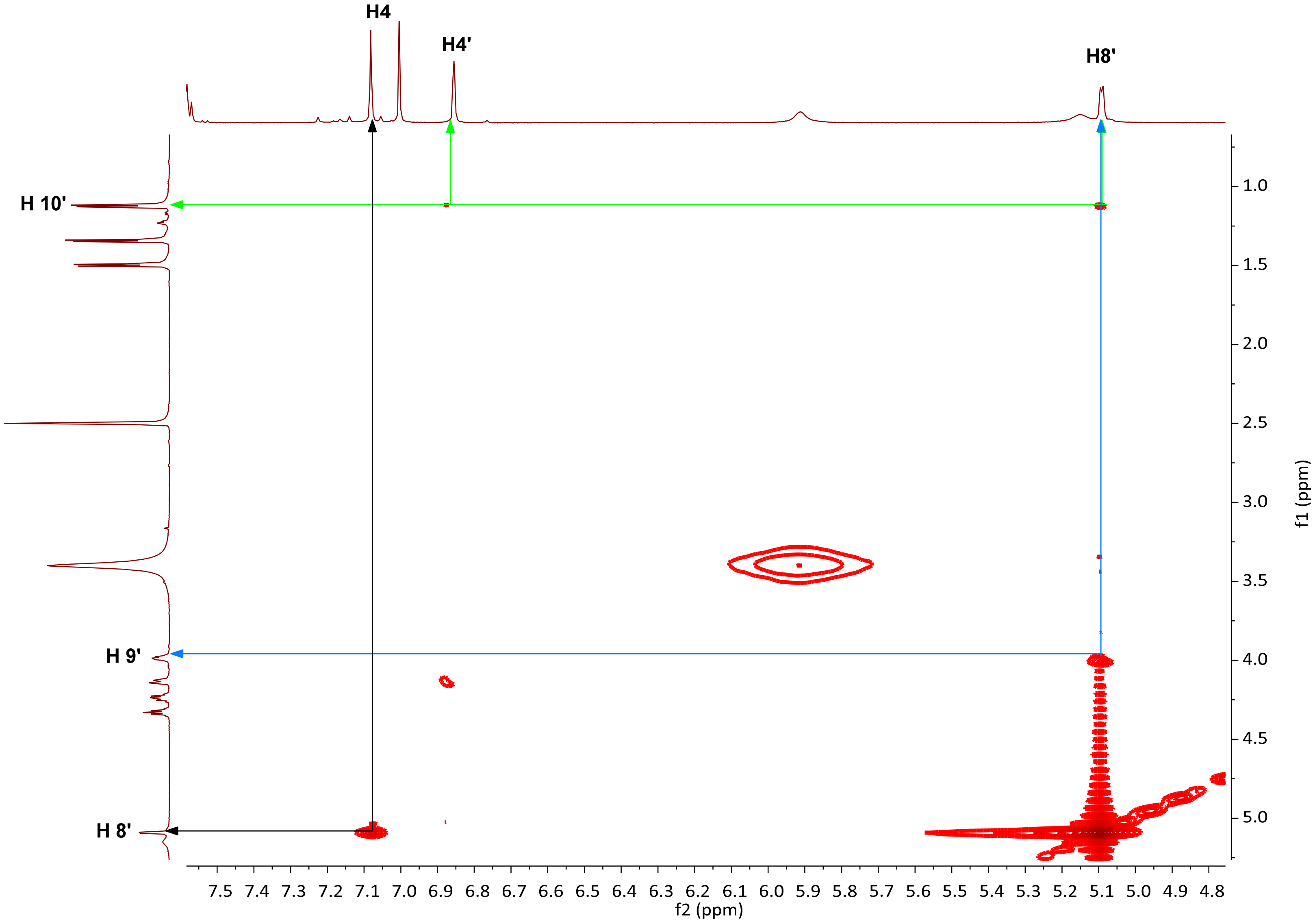

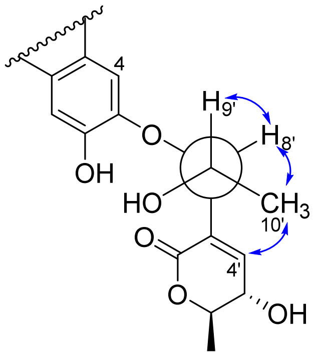

Figure S34. ^{1}H-NMR spectrum of ochrazepine B (**2**) in MeOH-d_4 at 1°C

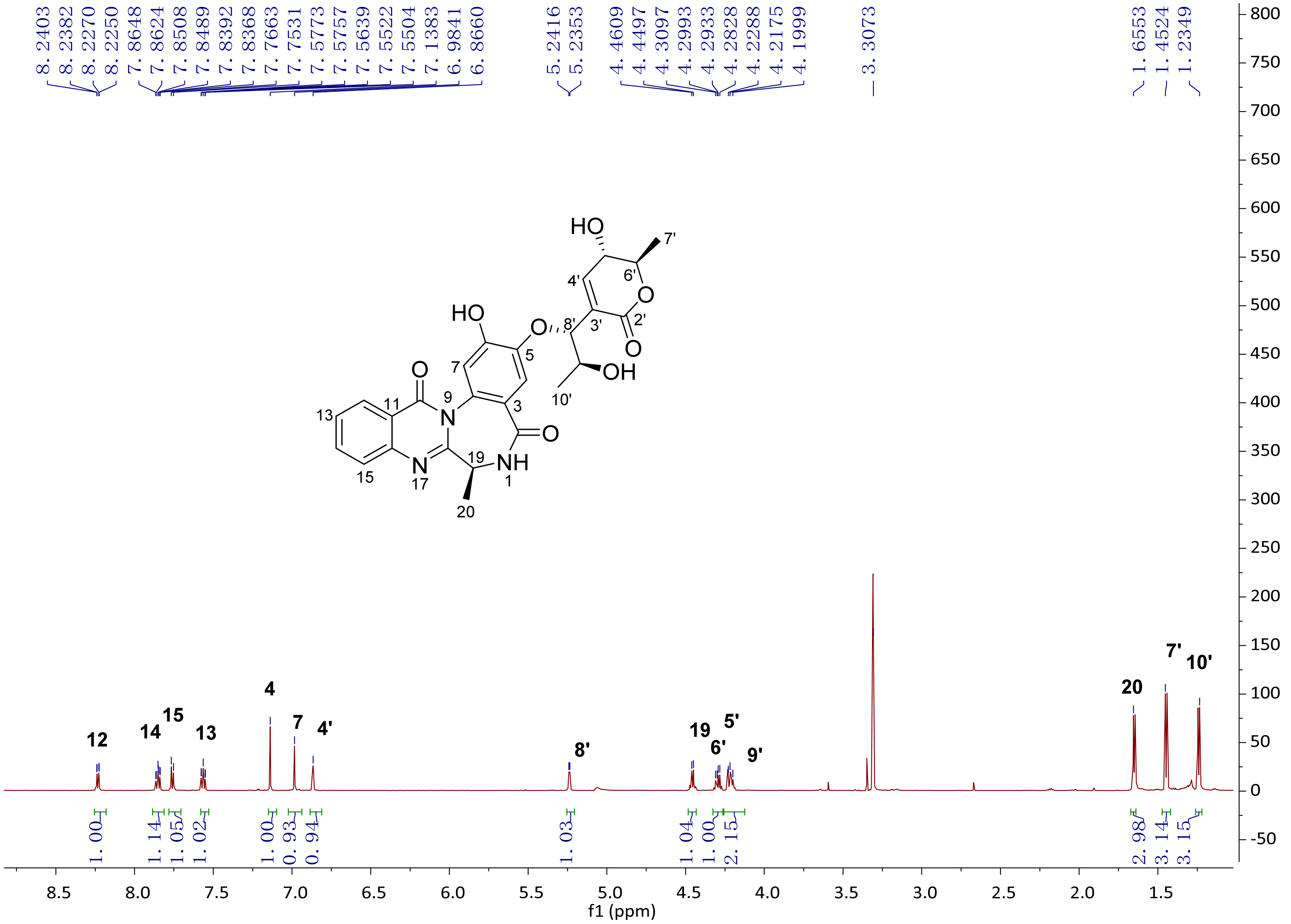

Figures S35. NOESY spectrum of ochrazepine B (**2**) in MeOH-d_4 at 1°C (1)

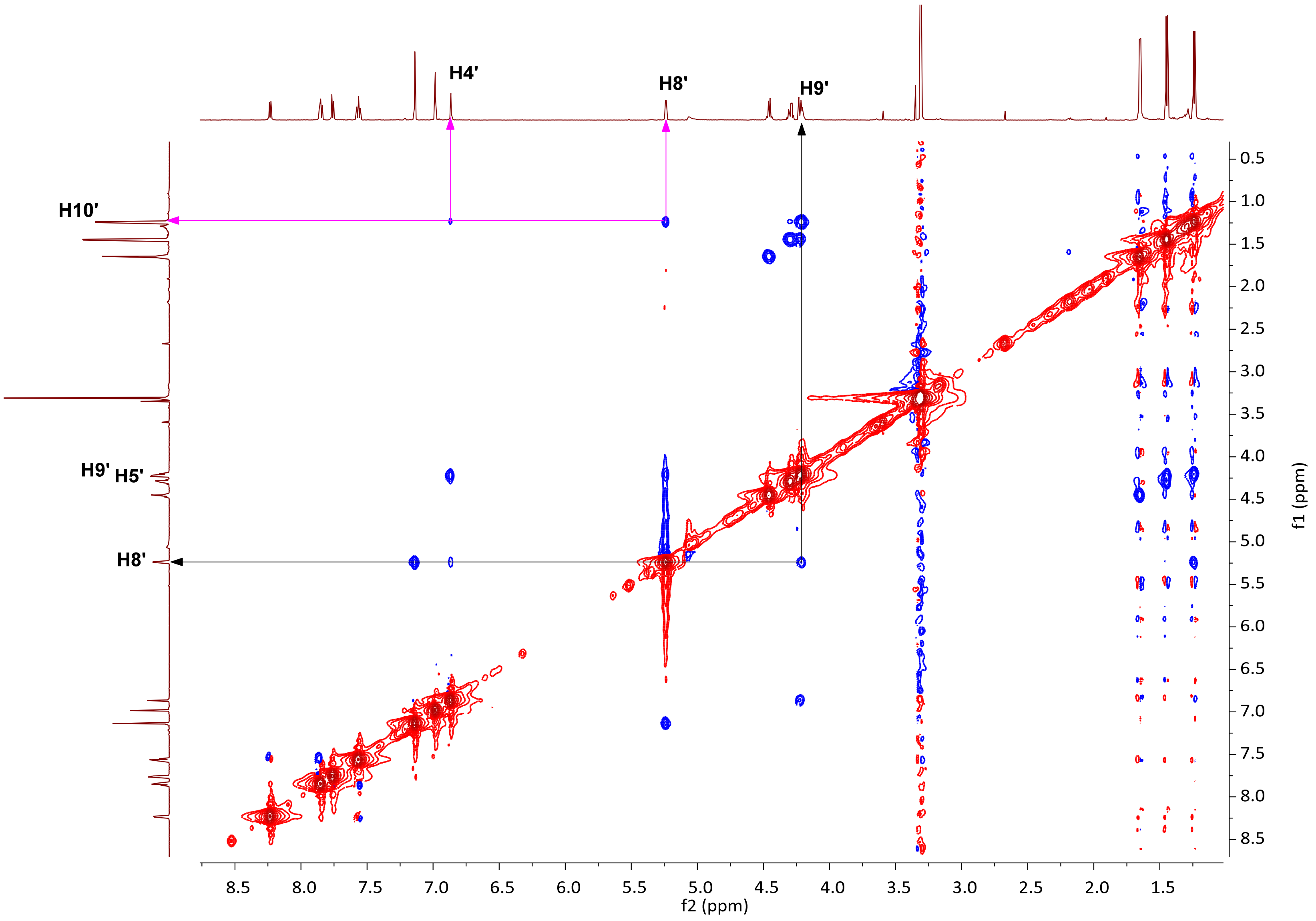

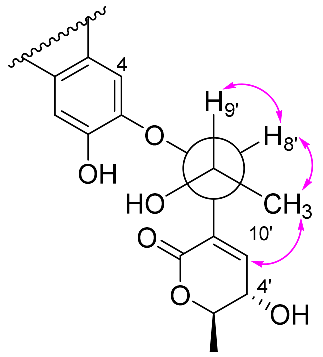

Figures S36. NOESY spectrum of ochrazepine B (**2**) in MeOH-d_4 at 1°C (2)

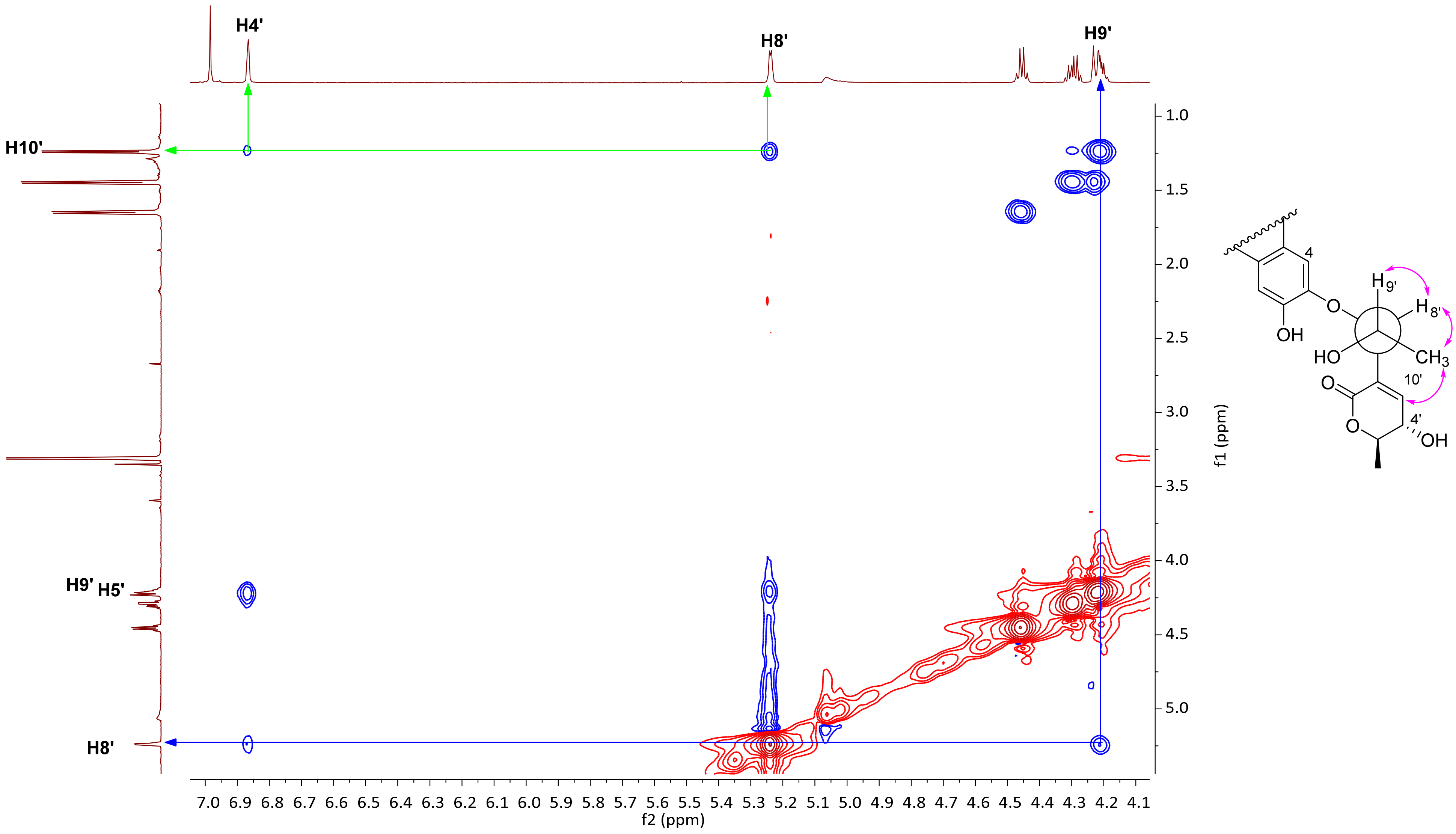

Figure S37. HRESIMS spectrum of ochrazepine C (**3**)

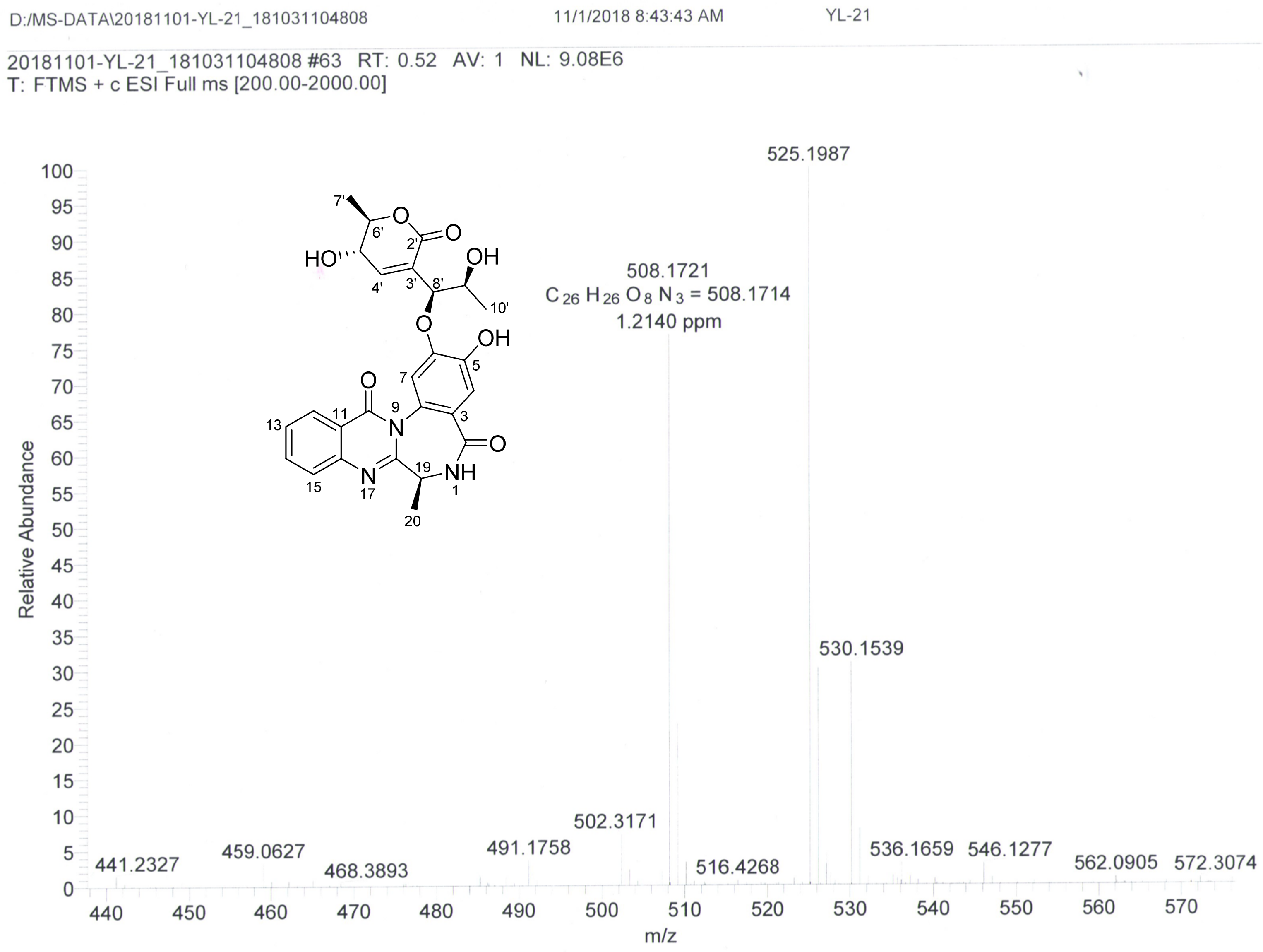

Figure S38. ^{1}H-NMR spectrum of ochrazepine C (**3**) in DMSO-d_6

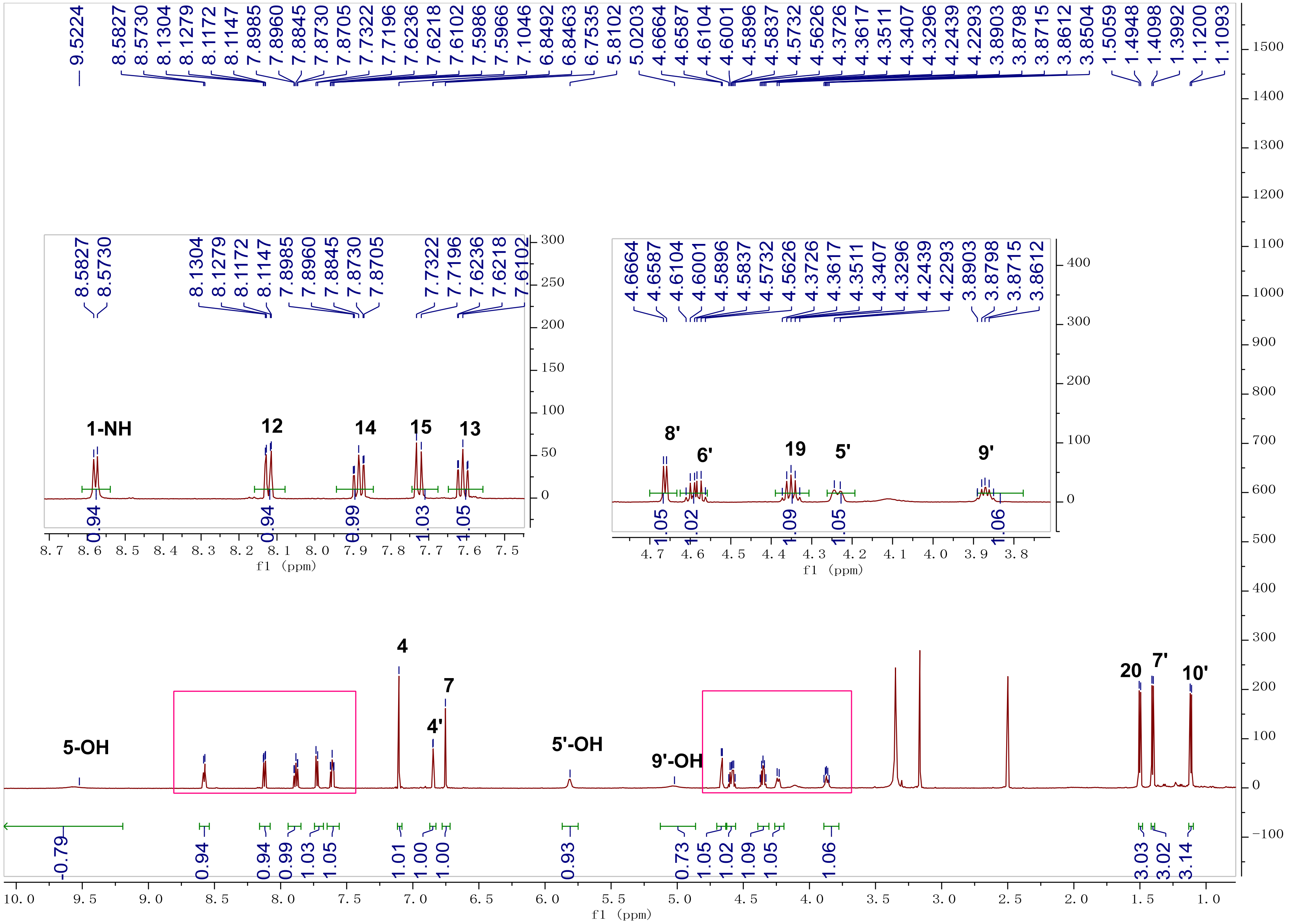

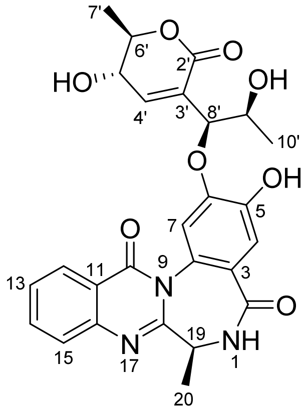

Figure S39. ^{13}C-NMR spectrum of ochrazepine C (**3**) in DMSO-d_6

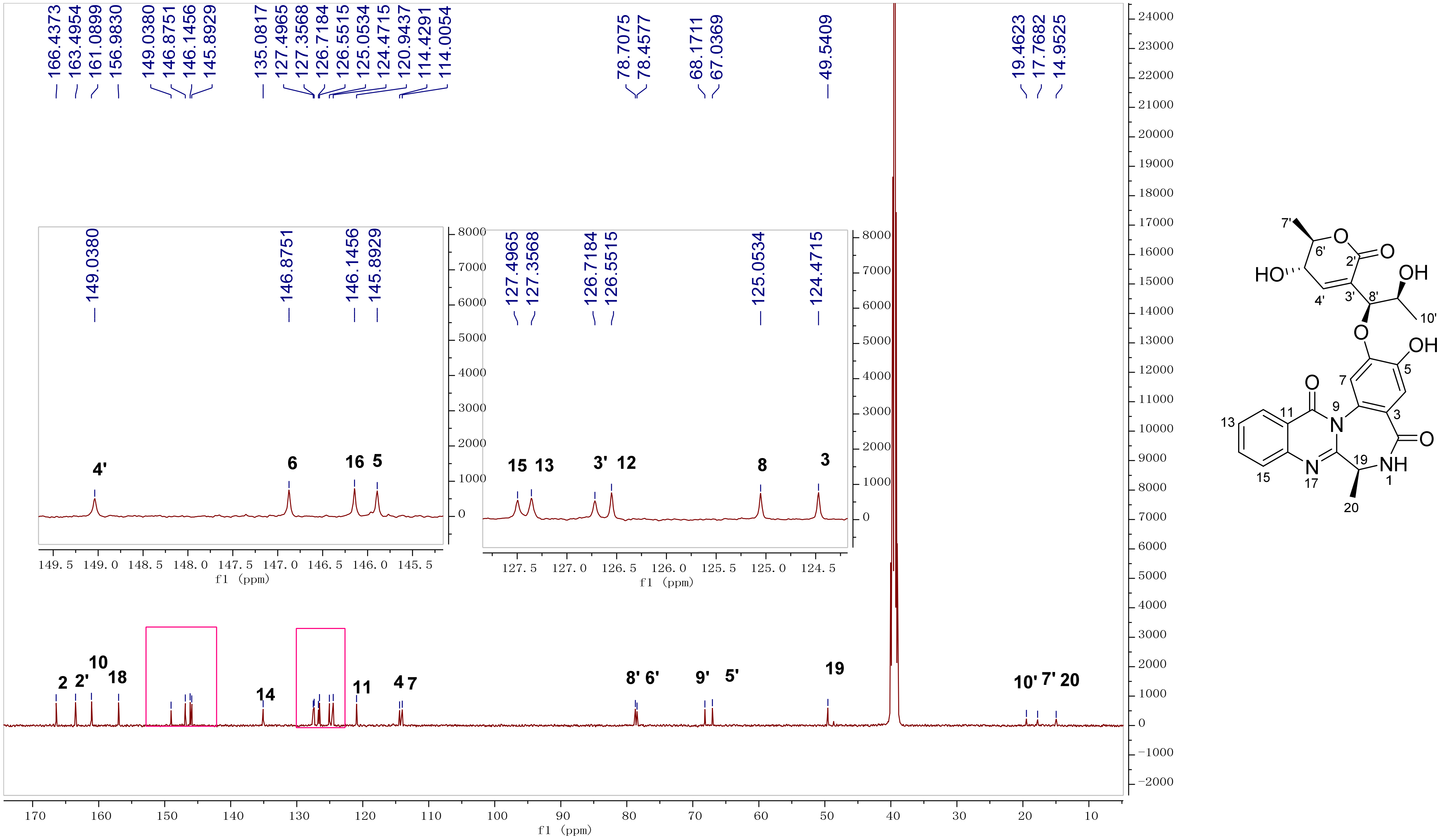

Figure S40. HMQC spectrum of ochrazepine C (**3**) in DMSO-d_6 (1)

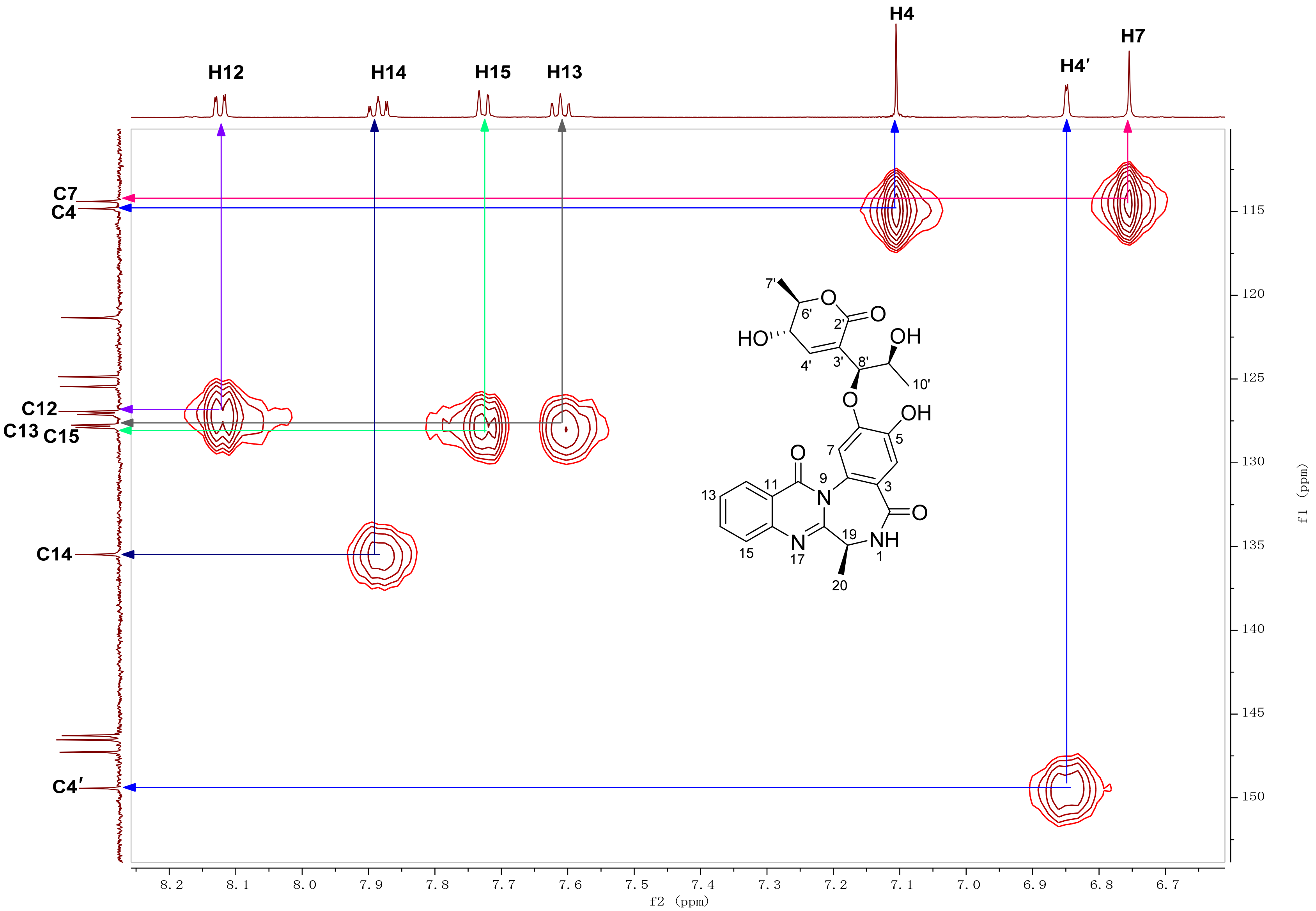

Figure S41. HMQC spectrum of ochrazepine C (**3**) in DMSO-d_6 (2)

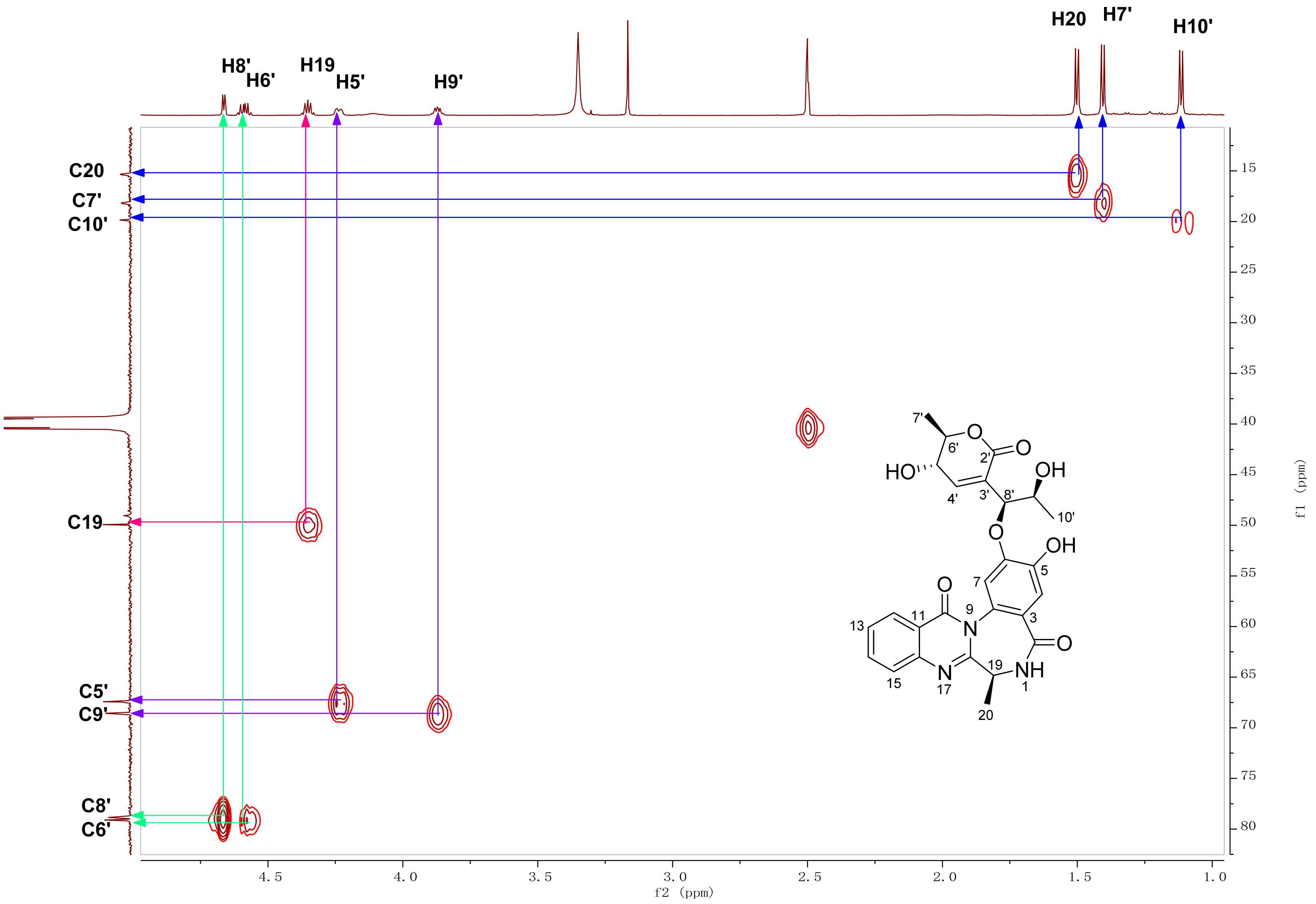

Figure S42. ^{1}H-^{1}H COSY spectrum of ochrazepine C (**3**) in DMSO-d_6 (1)

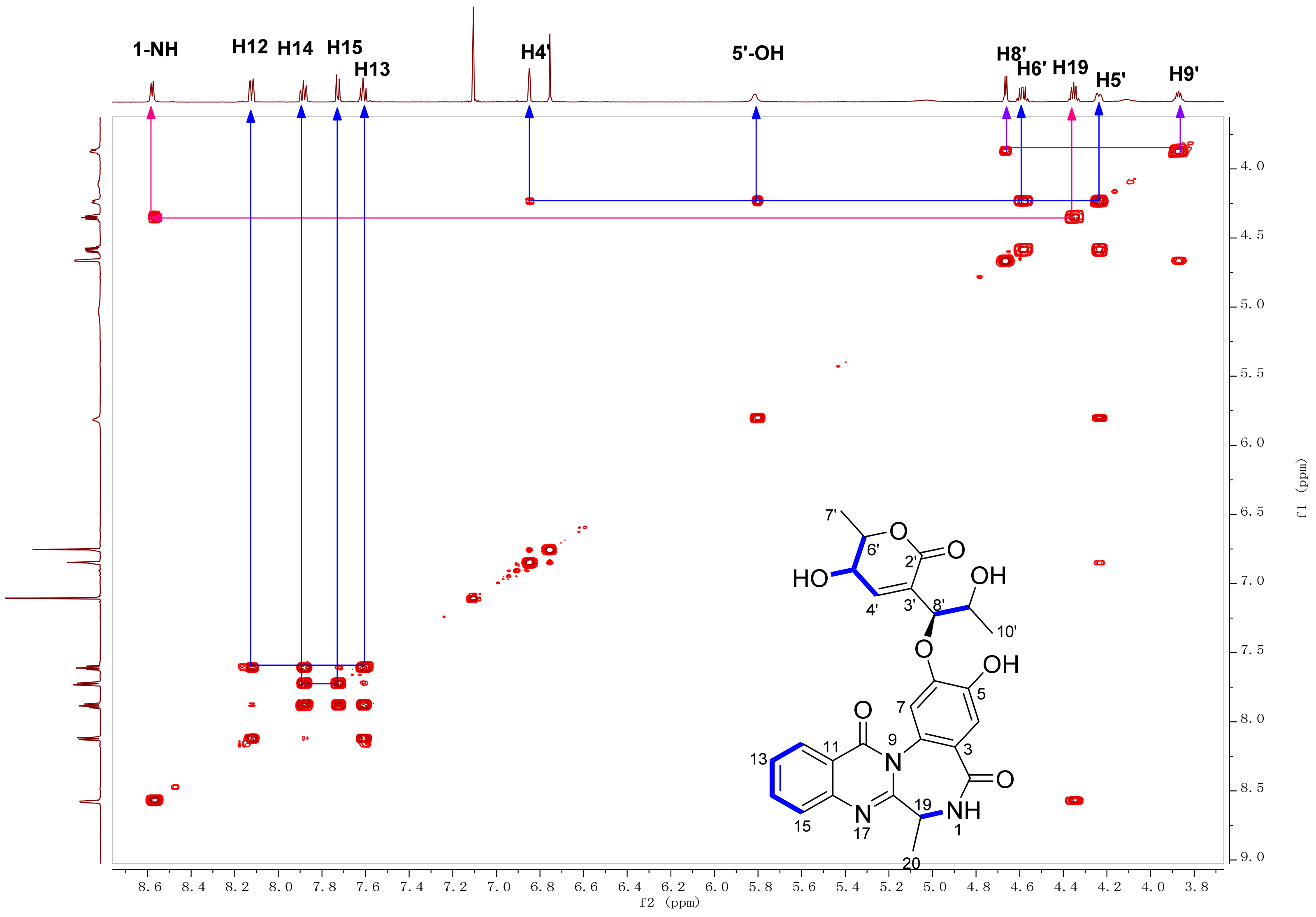

Figure S43. ^{1}H-^{1}H COSY spectrum of ochrazepine C (**3**) in DMSO-d_6 (2)

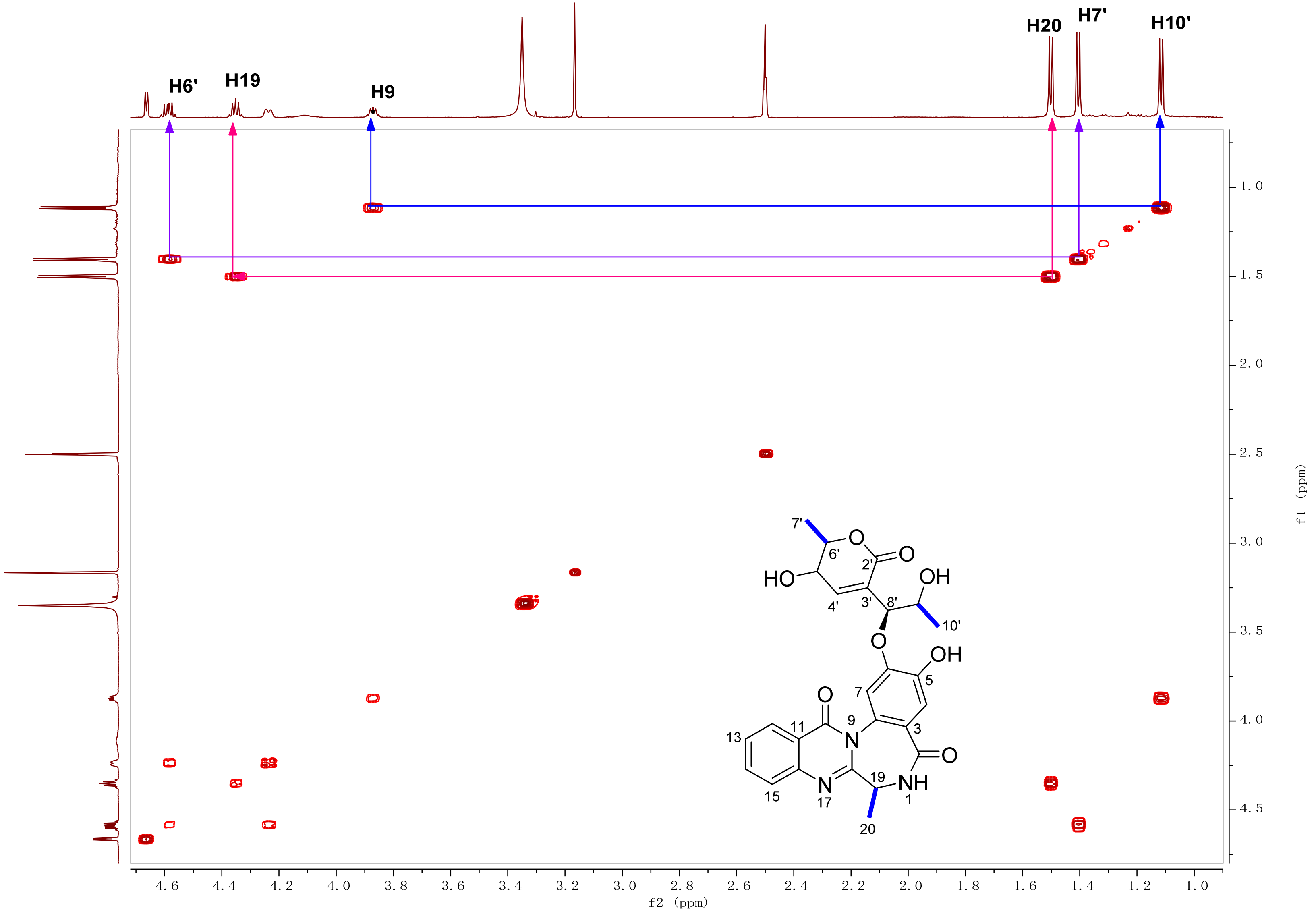

Figure S44. HMBC spectrum of ochrazepine C (**3**) in DMSO-d_6 (1)

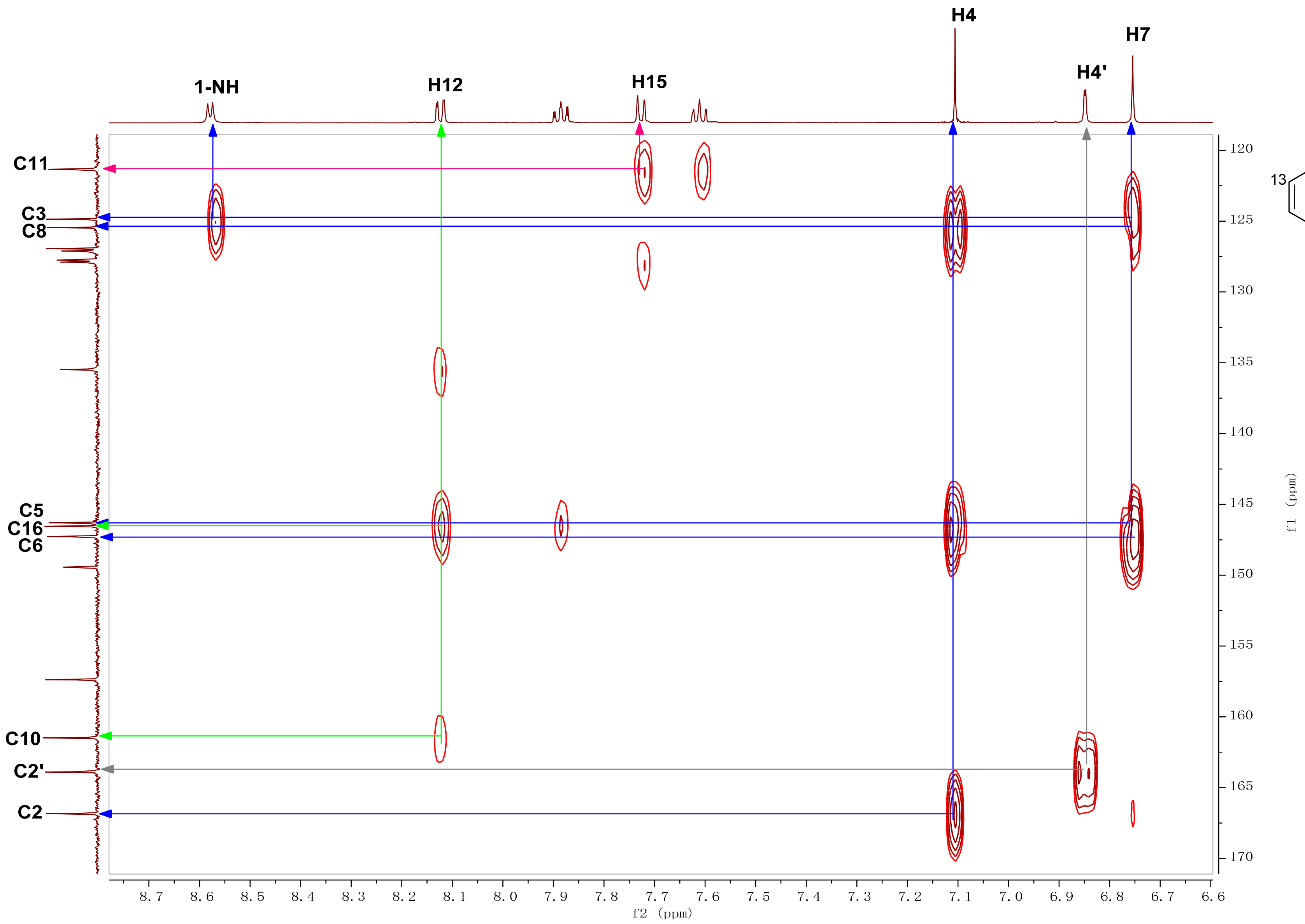

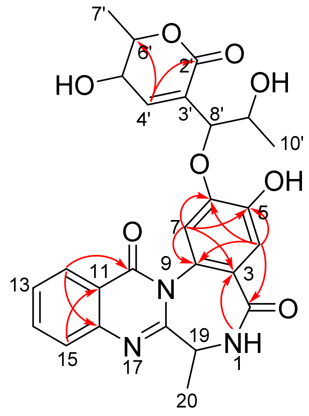

Figure S45. HMBC spectrum of ochrazepine C (**3**) in DMSO-d_6 (2)

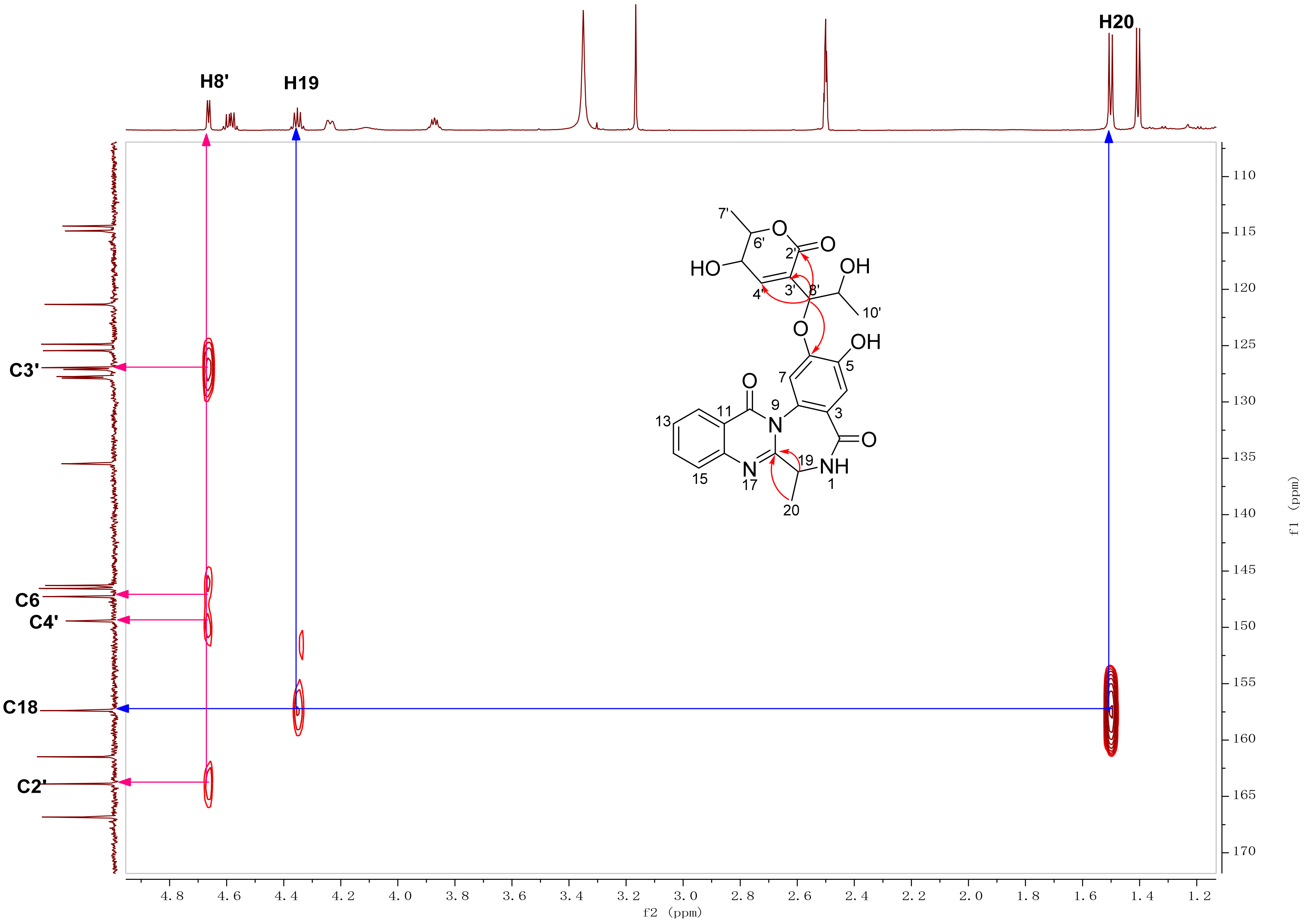

Figure S46. HMBC spectrum of ochrazepine C (**3**) in DMSO-d_6 (3)

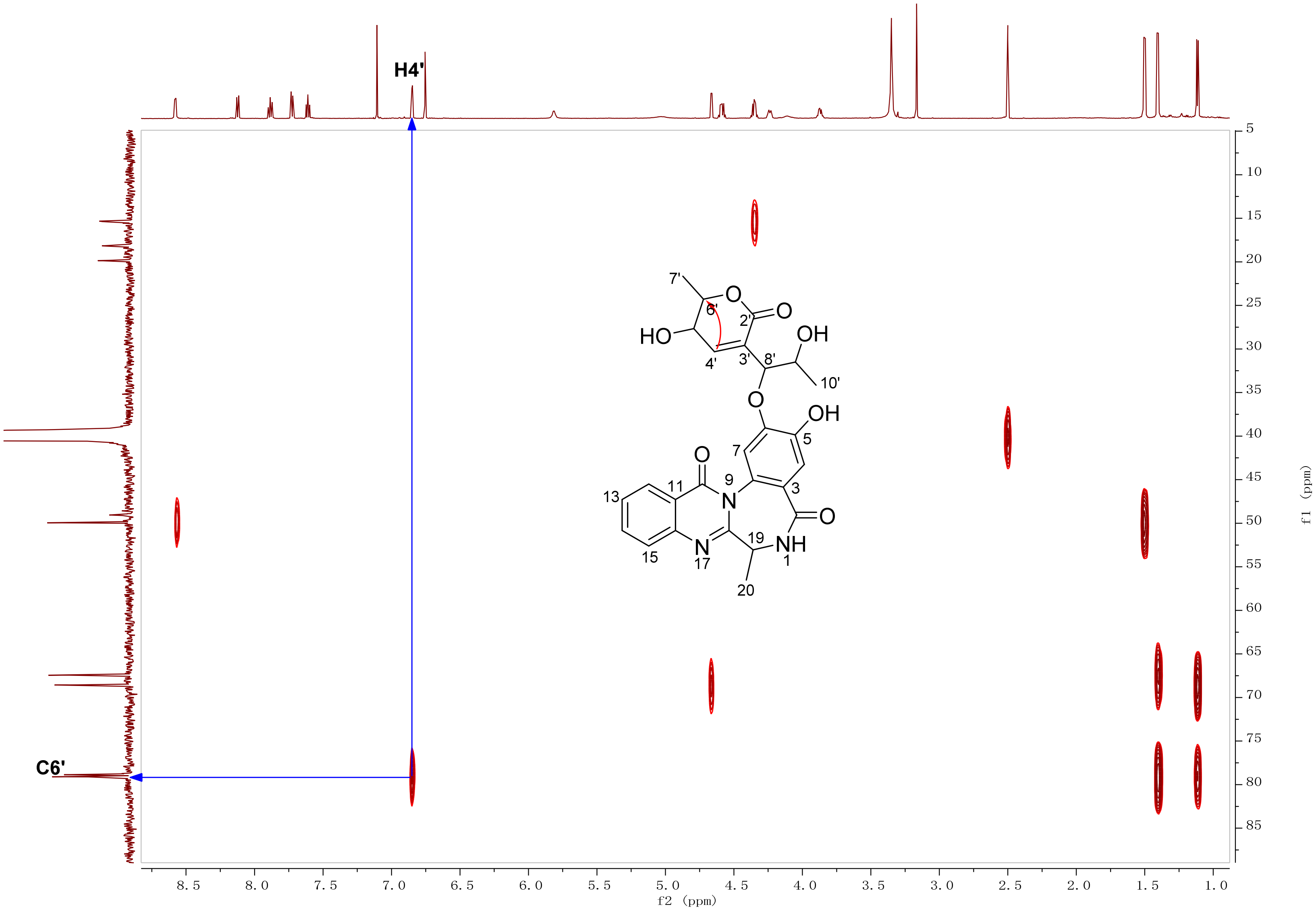

Figure S47. NOESY spectrum of ochrazepine C (**3**) in DMSO-d_6 at 25ºC (1)

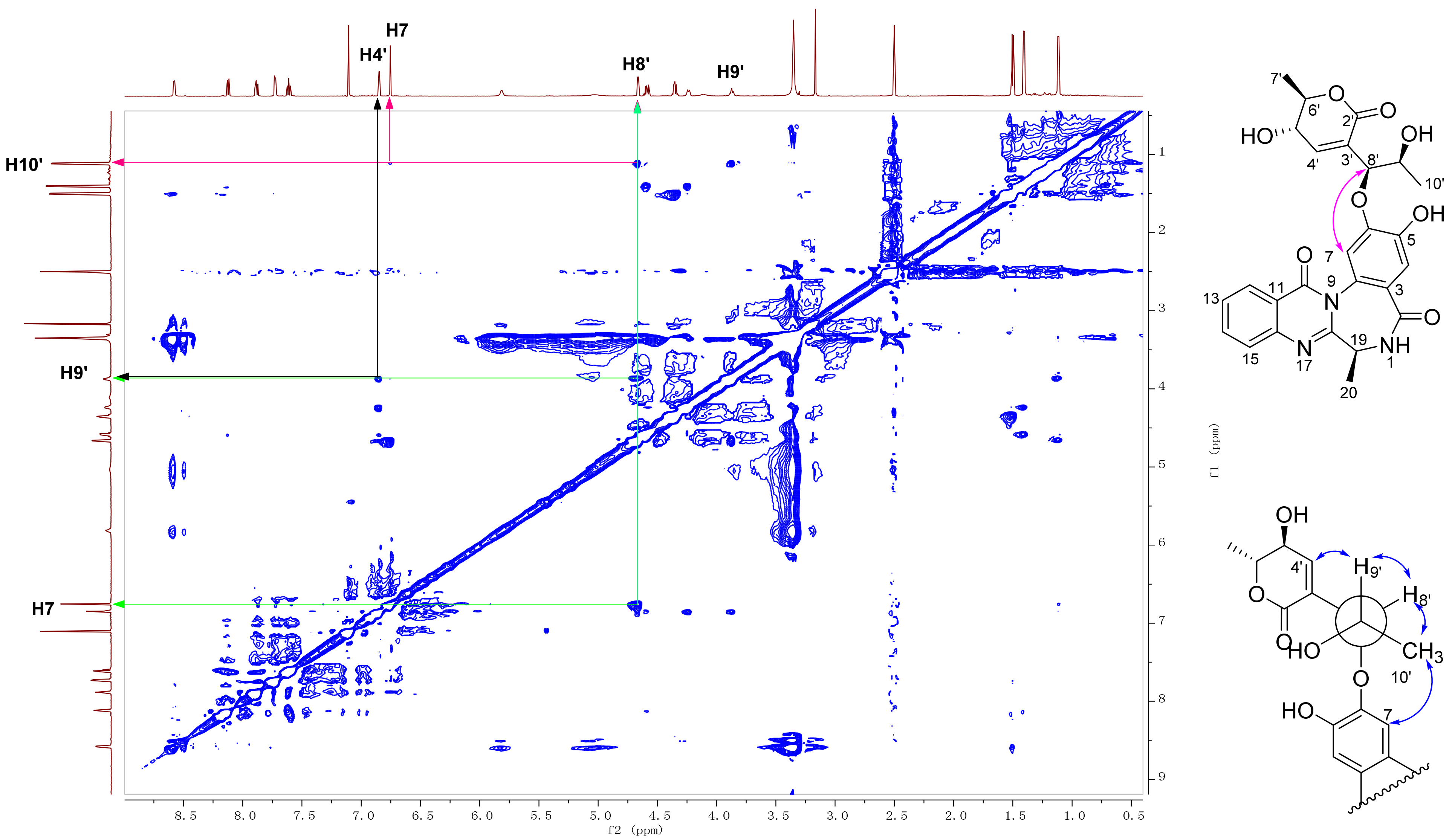

Figure S48. NOESY spectrum of ochrazepine C (**3**) in DMSO-d_6 at 25°C (2)

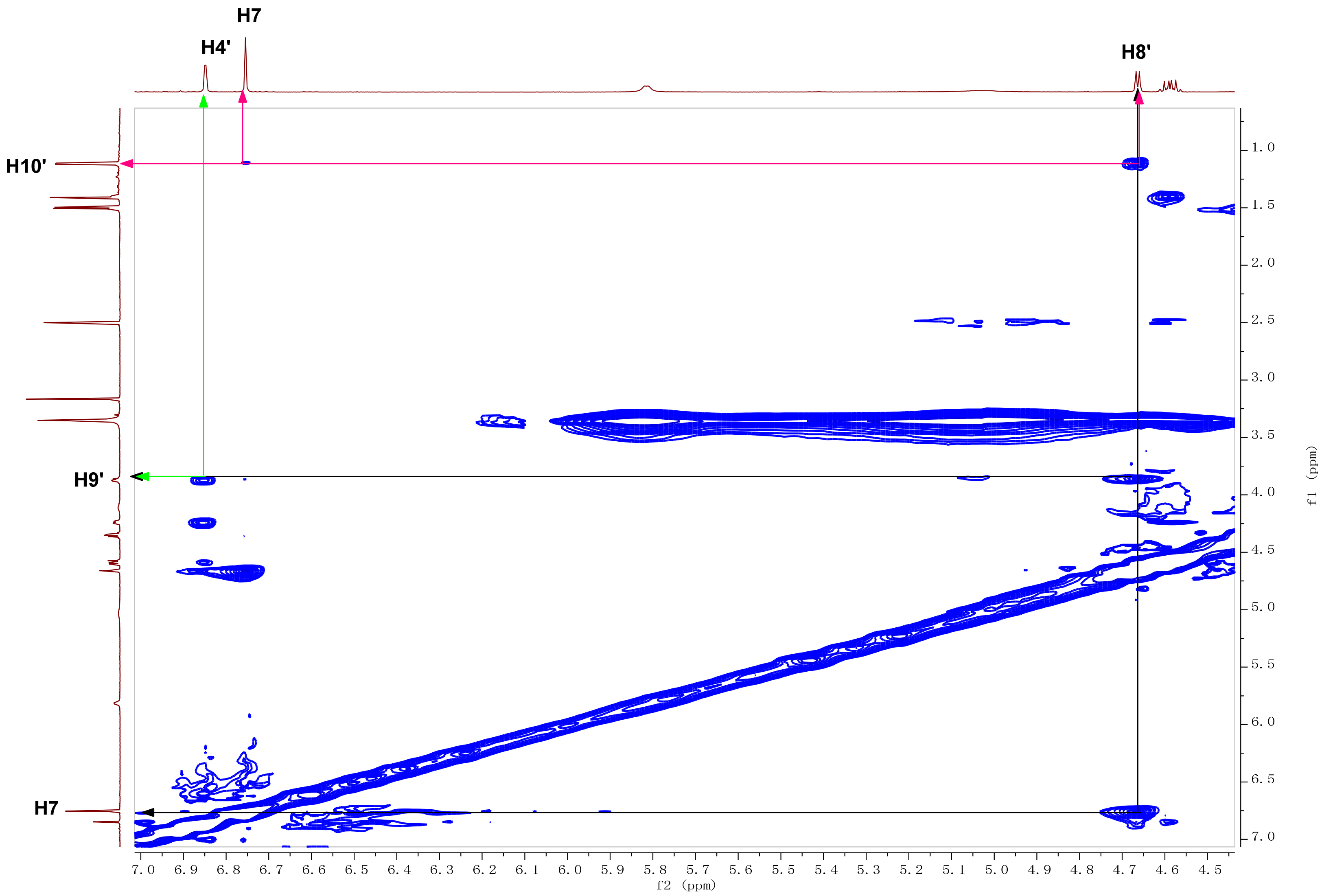

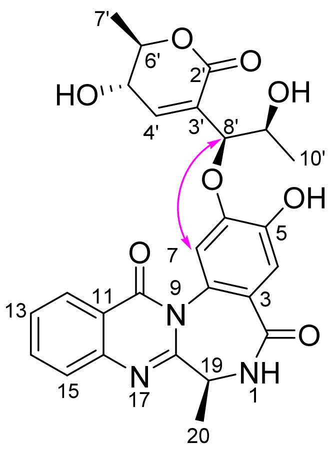

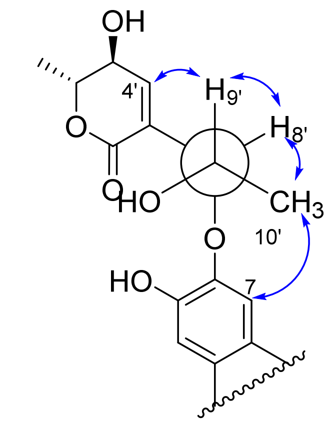

Figure S49. ^{1}H-NMR spectrum of ochrazepine C (**3**) in MeOH-d_4 at 1°C

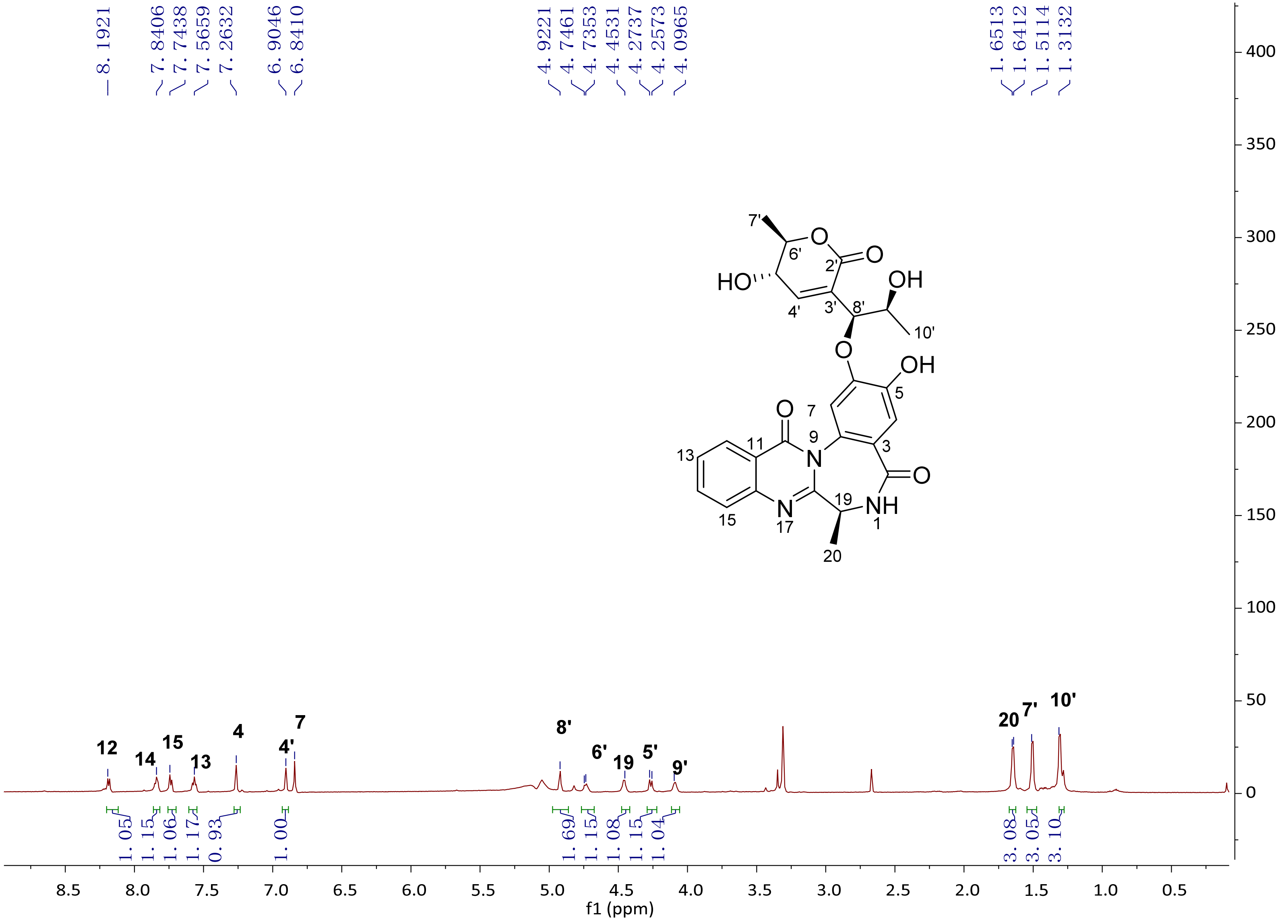

Figures S50. NOESY spectrum of ochrazepine C (**3**) in MeOH-d_4 at 1°C

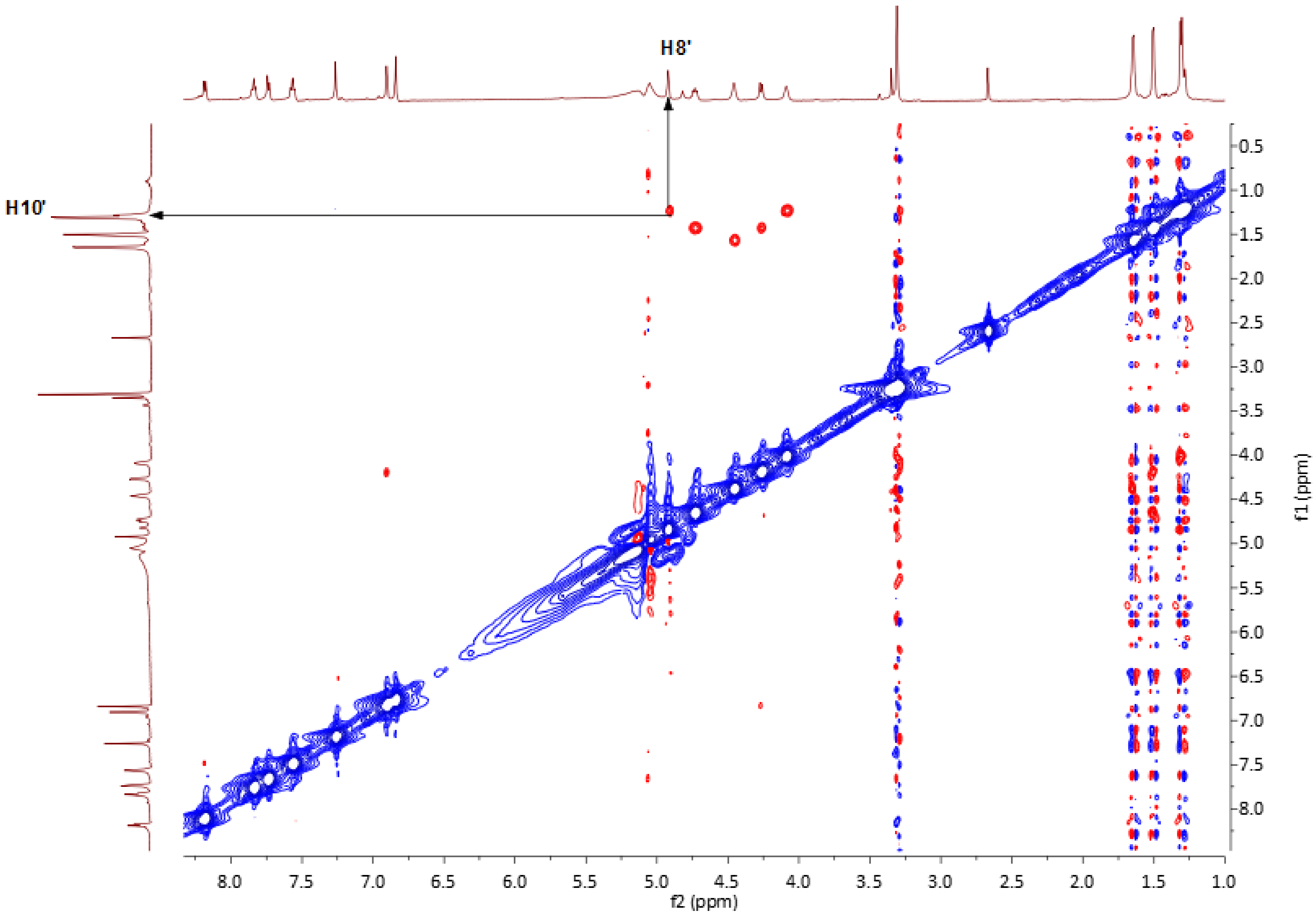

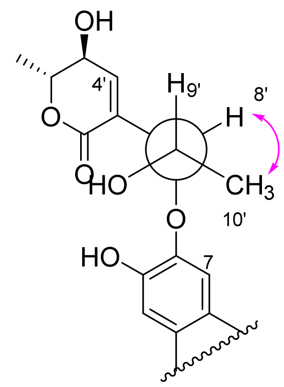

Figure S51. HRESIMS spectrum of ochrazepine D (**4**)

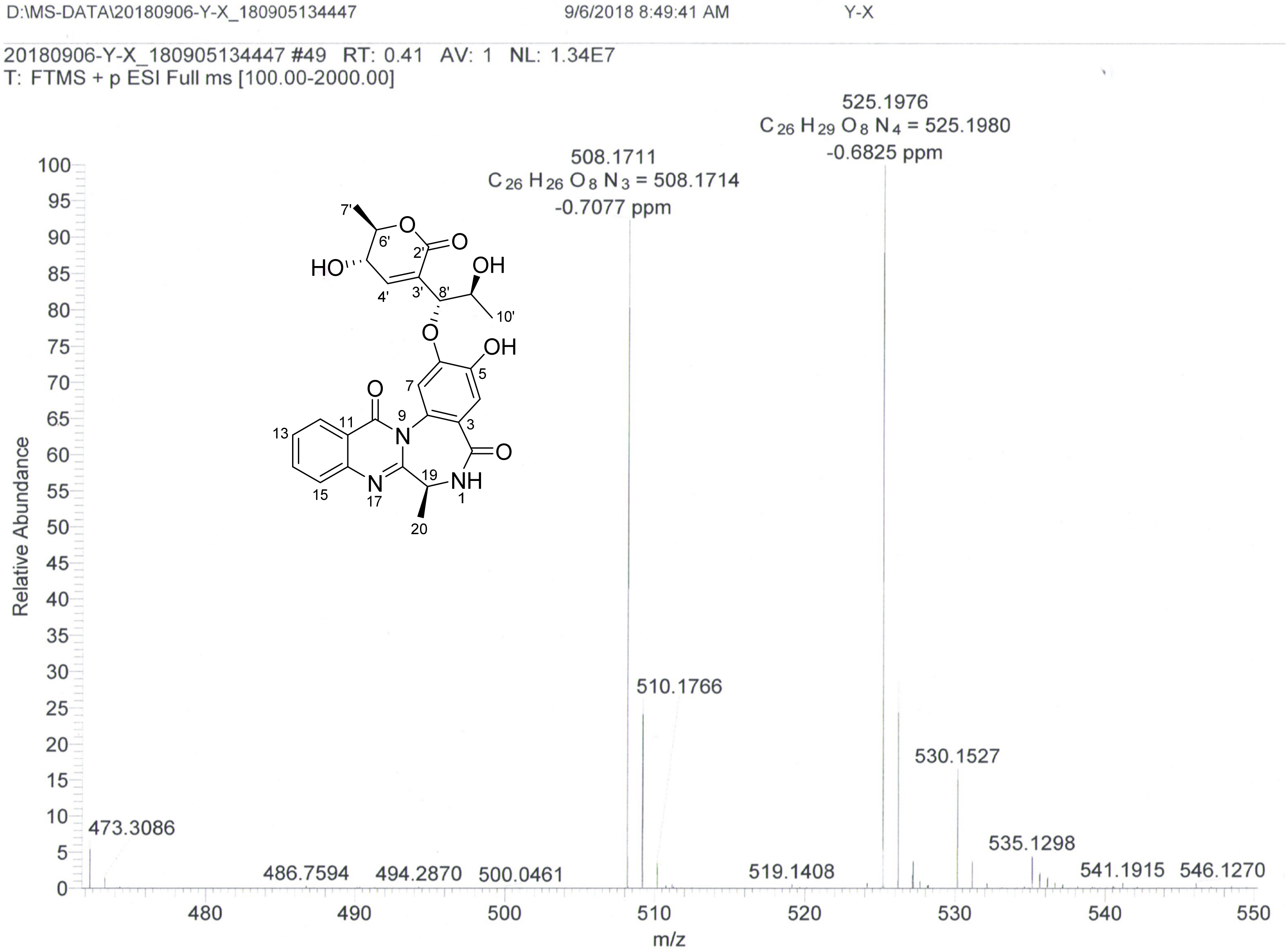

Figure S52. ^{1}H-NMR spectrum of ochrazepine D (**4**) in DMSO-d_6

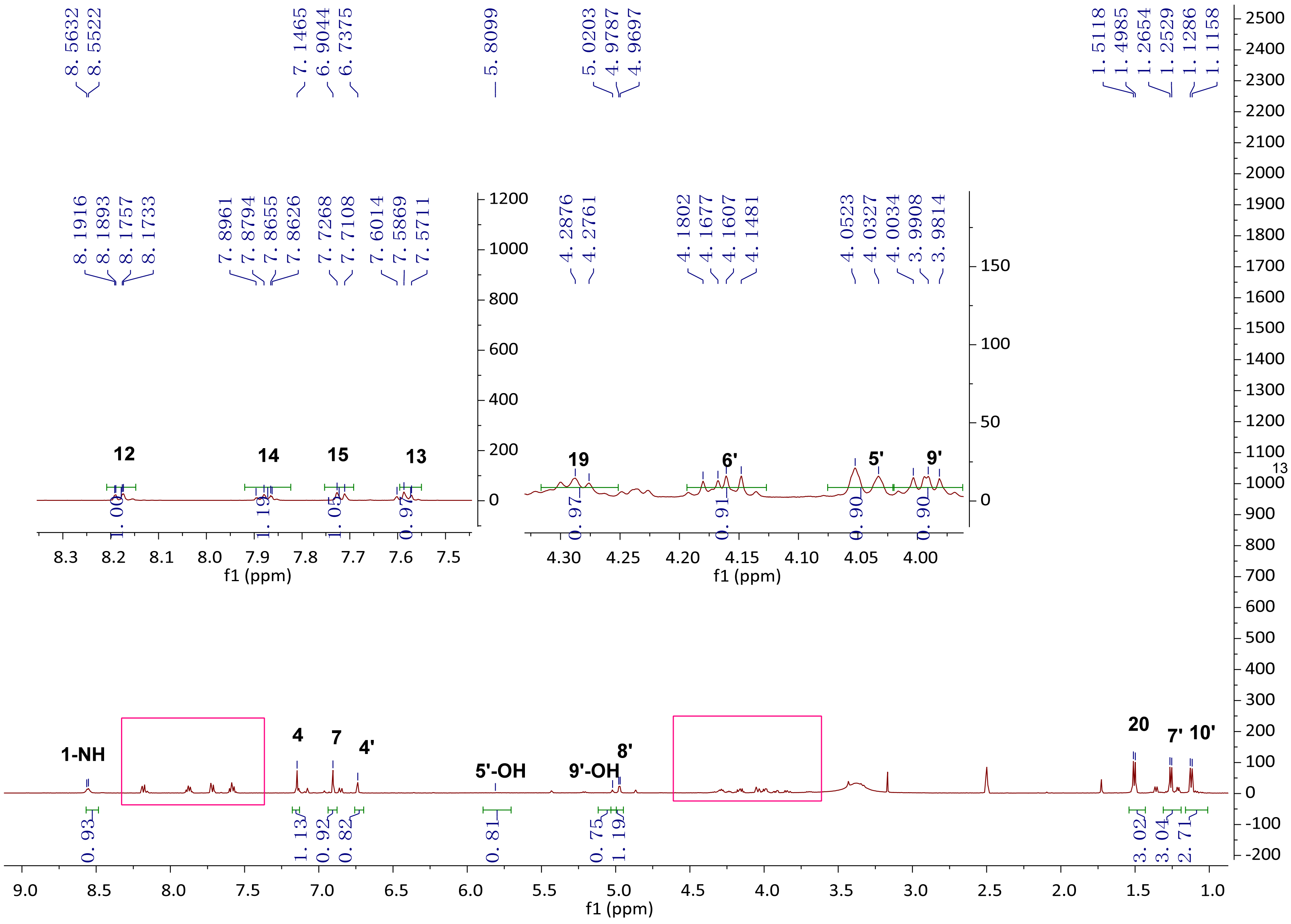

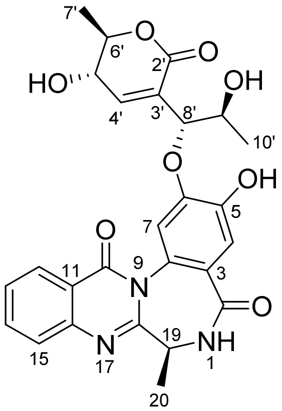

Figure S53. ^{13}C-NMR spectrum of ochrazepine D (**4**) in DMSO-d_6

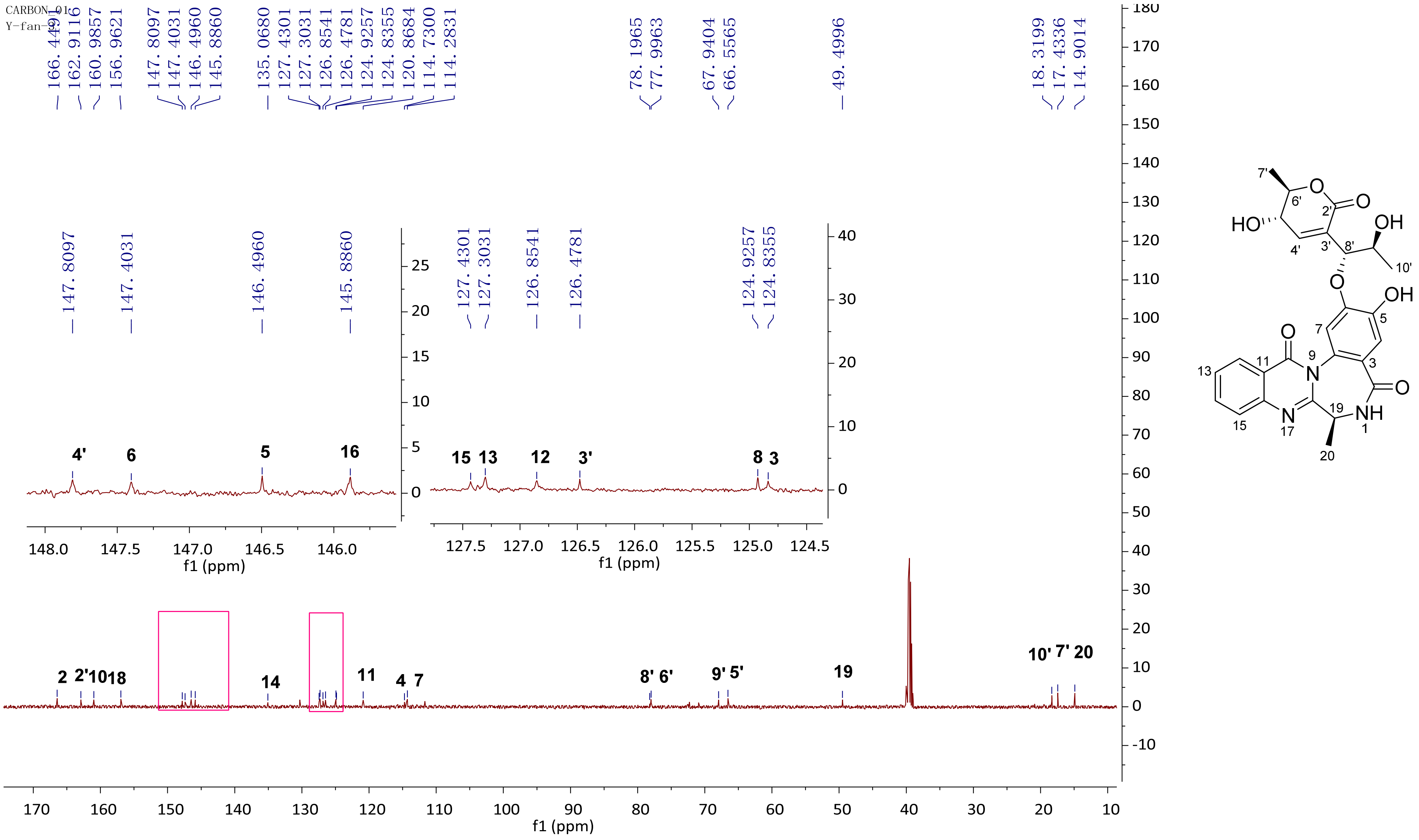

Figure S54. HSQC spectrum of ochrazepine D (**4**) in DMSO-d_6 (1)

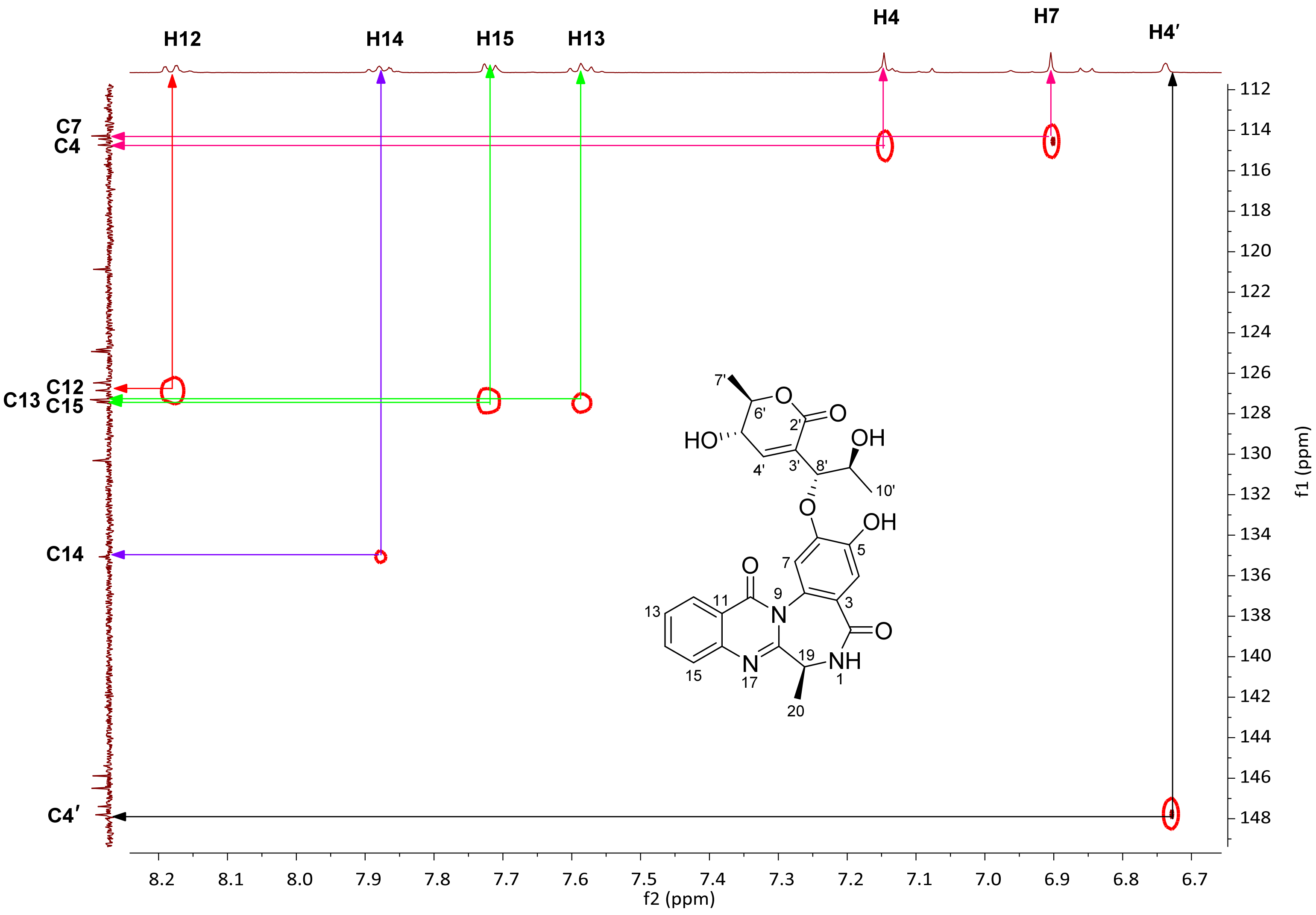

Figure S55. HSQC spectrum of ochrazepine D (**4**) in DMSO-d_6 (2)

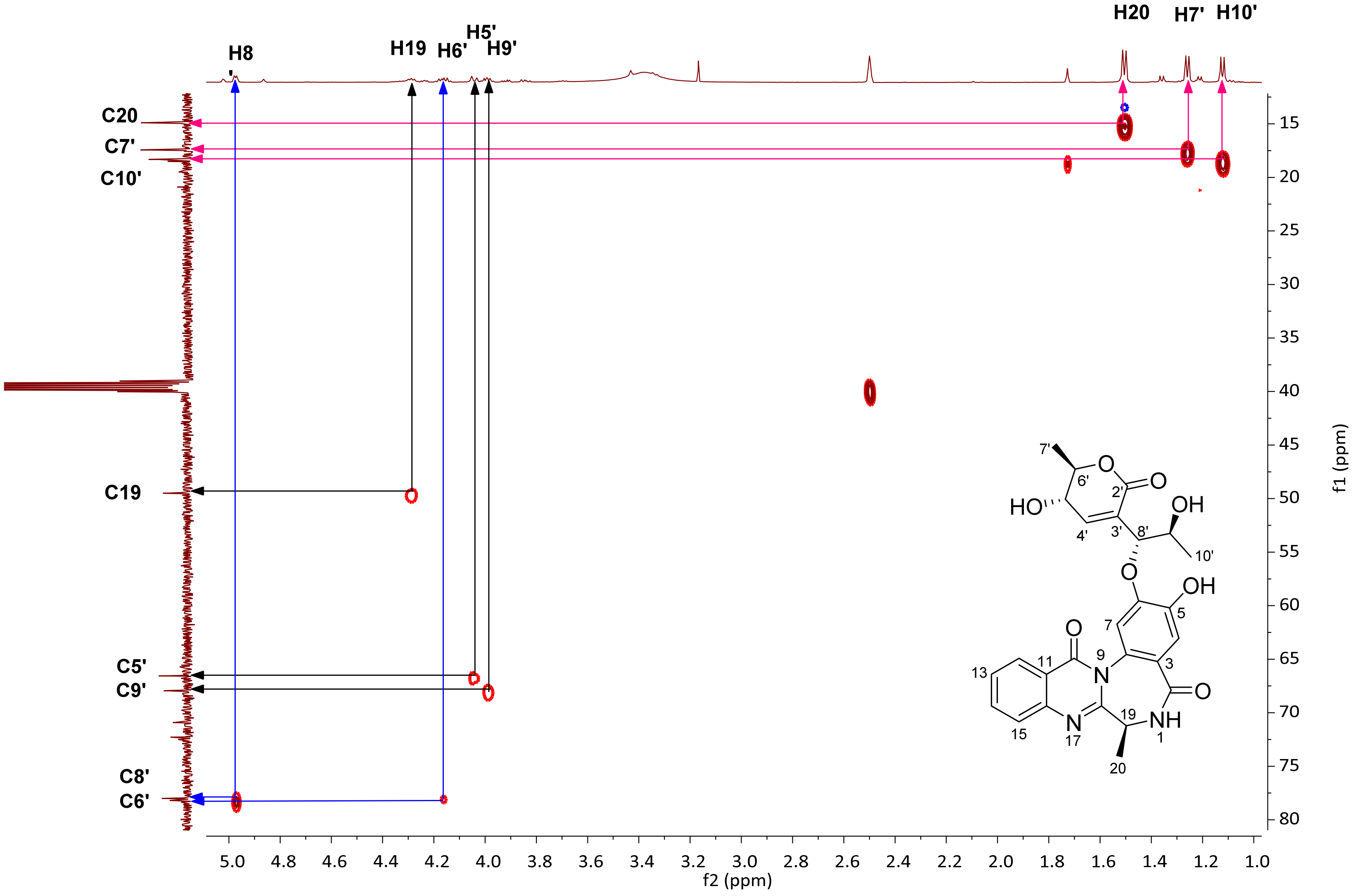

Figure S56. ^{1}H-^{1}H COSY spectrum of ochrazepine D (**4**) in DMSO-d_6 (1)

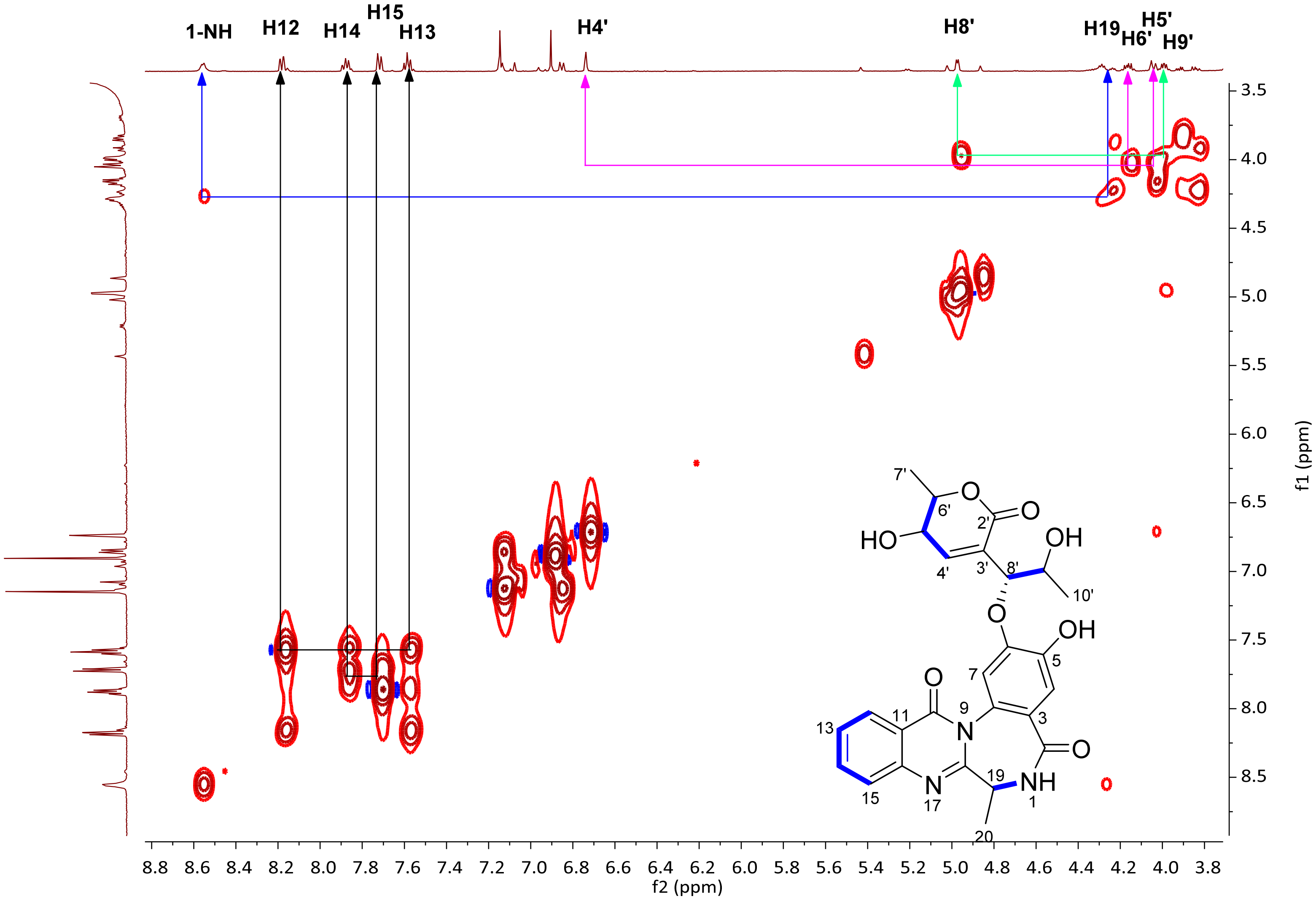

Figure S57. ^{1}H-^{1}H COSY spectrum of ochrazepine D (**4**) in DMSO-d_6 (2)

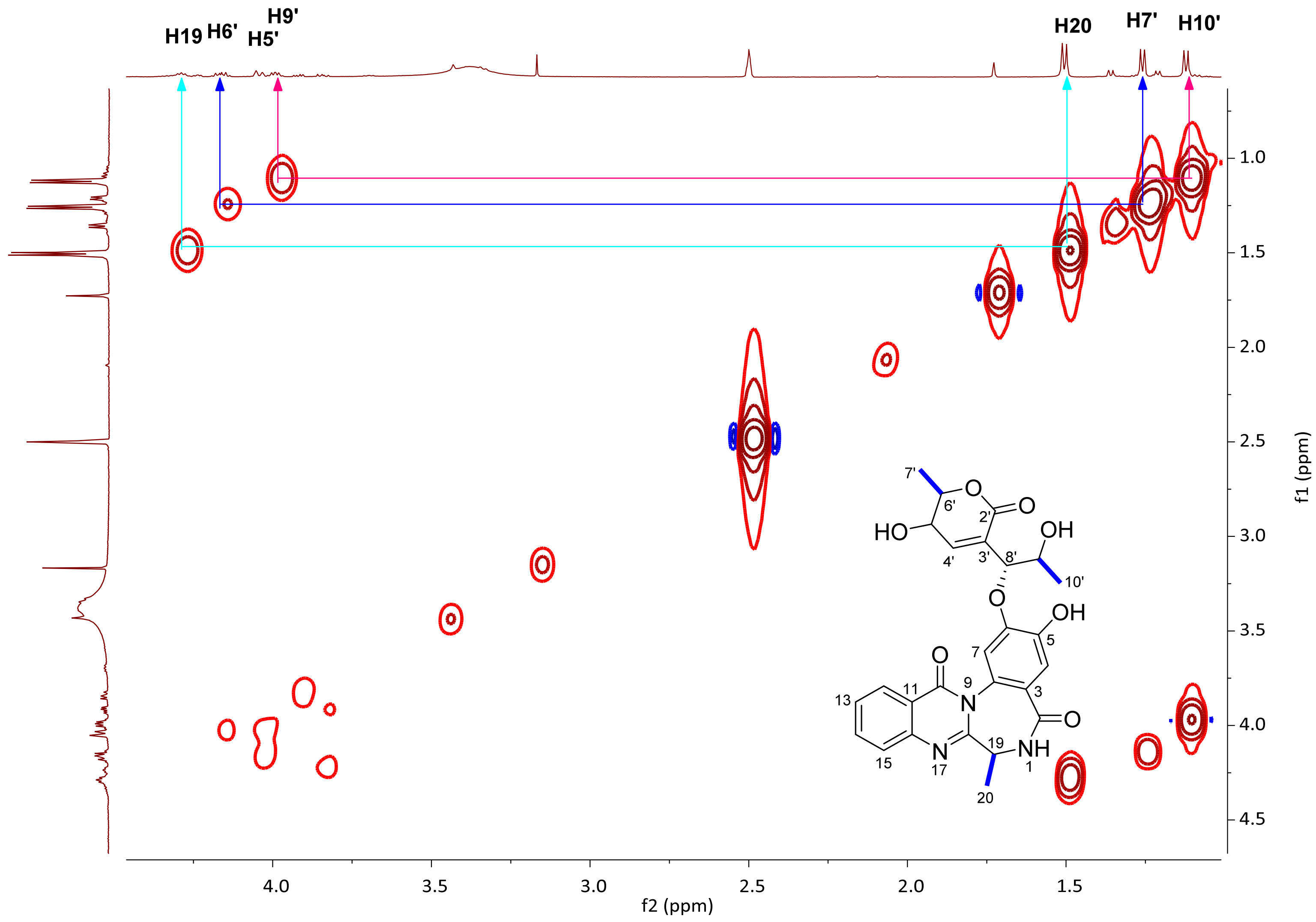

Figure S58. HMBC spectrum of ochrazepine D (**4**) in DMSO-d_6 (1)

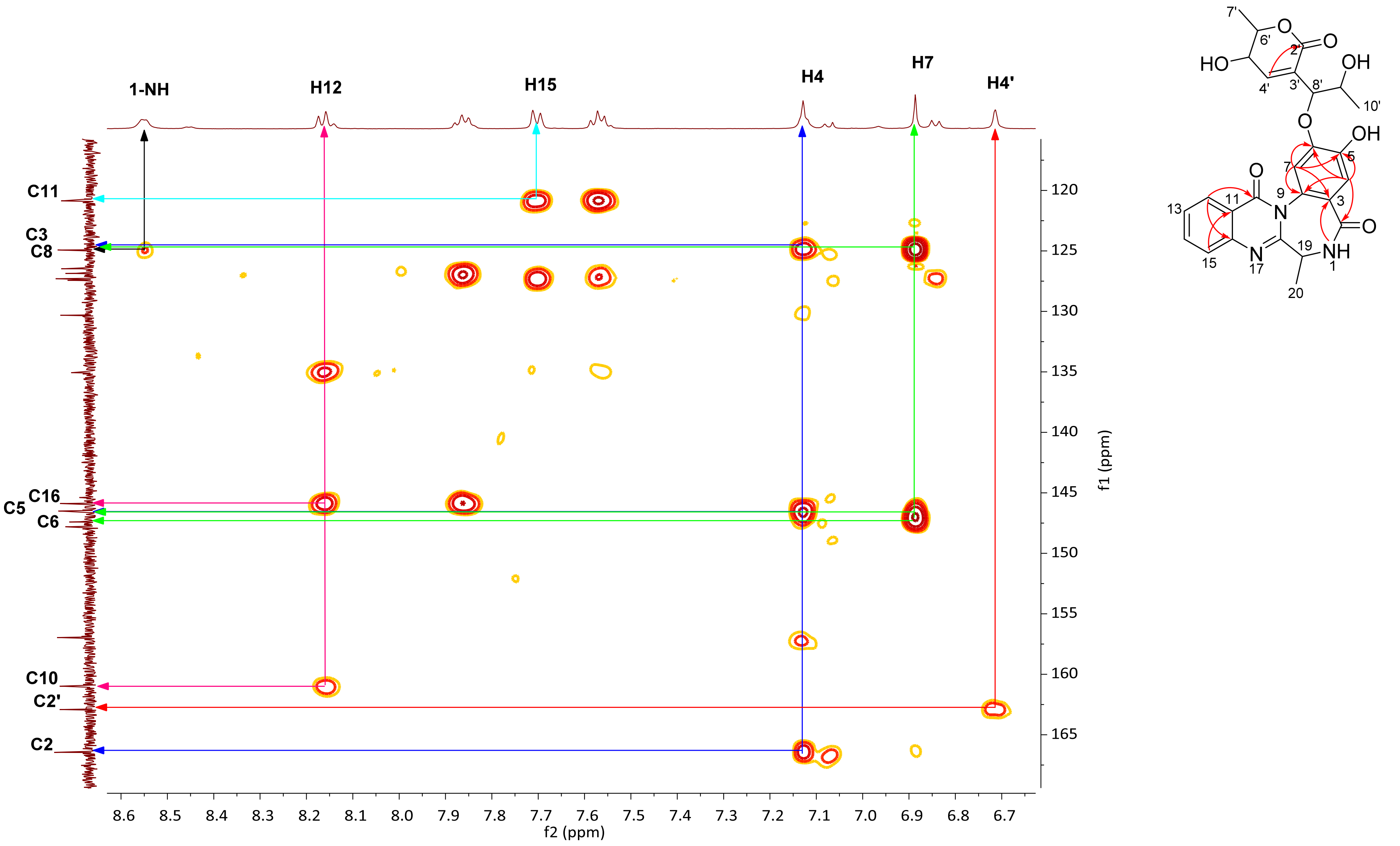

Figure S59. HMBC spectrum of ochrazepine D (**4**) in DMSO-d_6 (2)

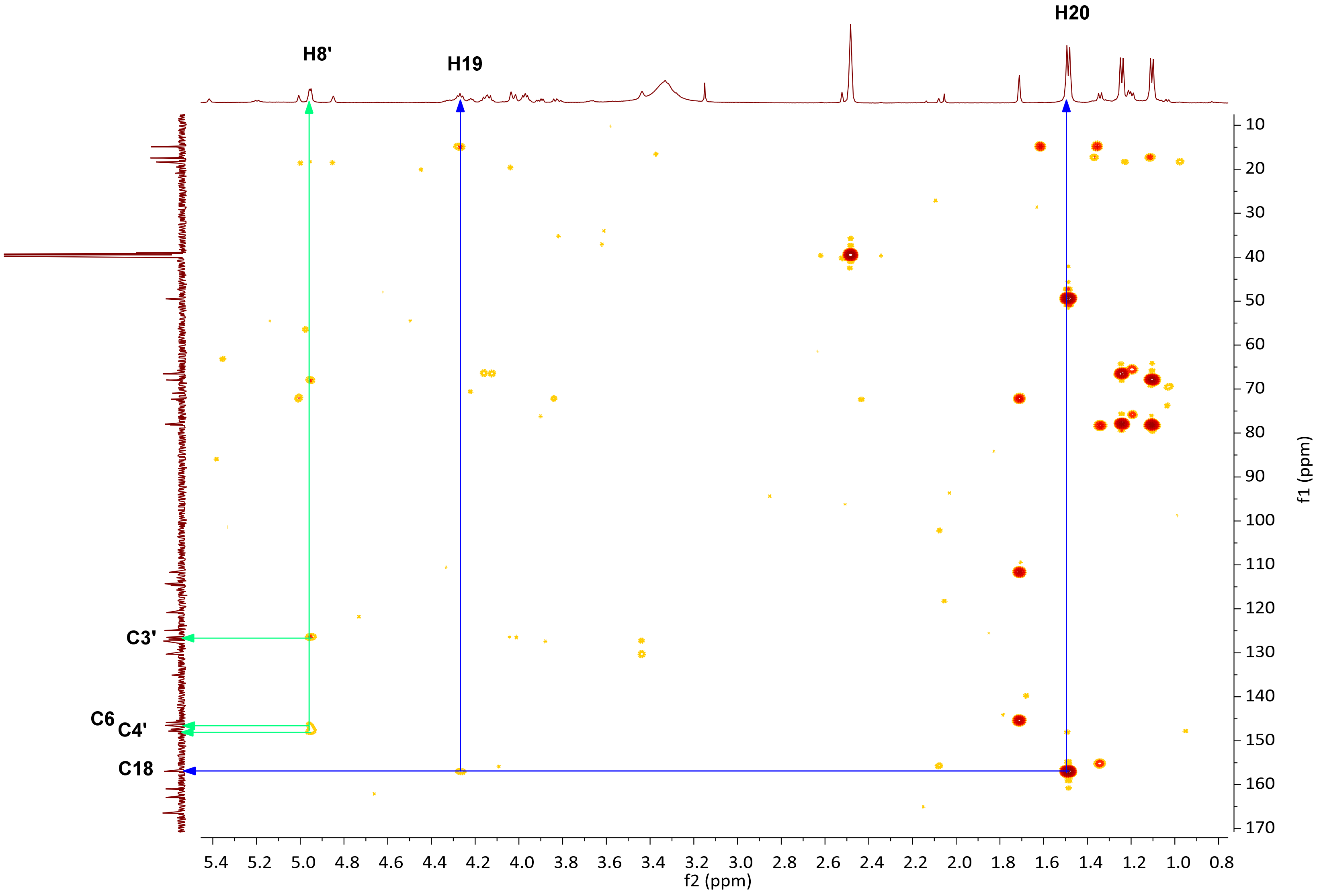

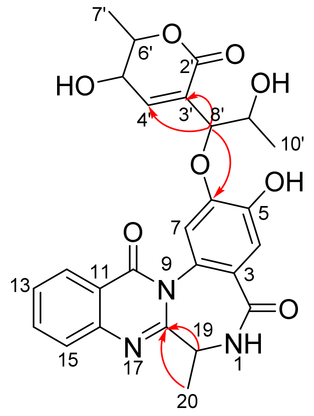

Figure S60. HMBC spectrum of ochrazepine D (**4**) in DMSO-d_6 (3)

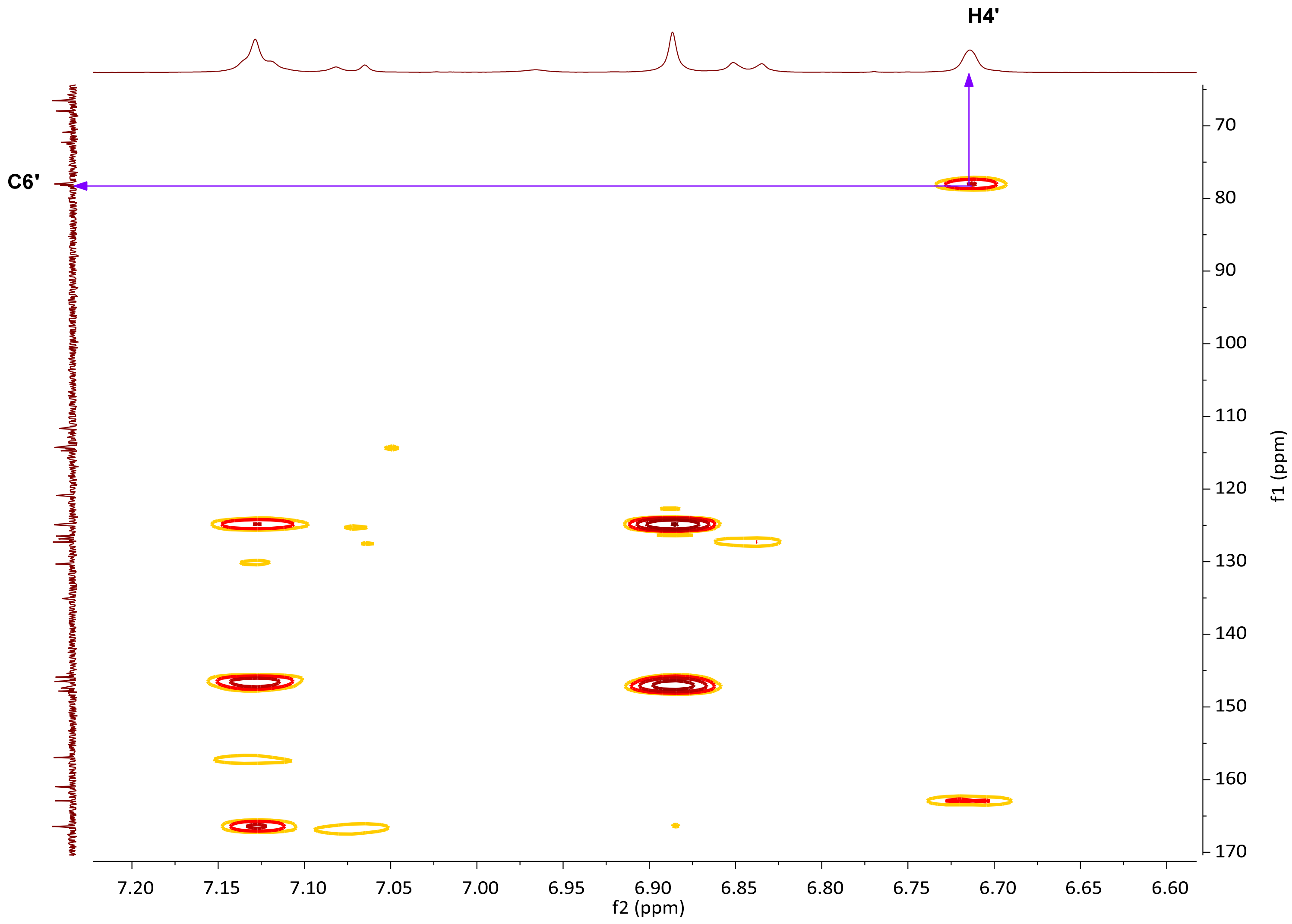

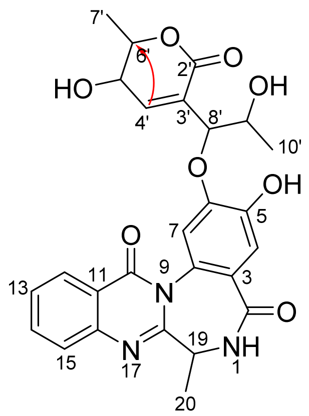

Figure S61. NOESY spectrum of ochrazepine D (**4**) in DMSO-*d*$_6$ (1) at 25°C (1)

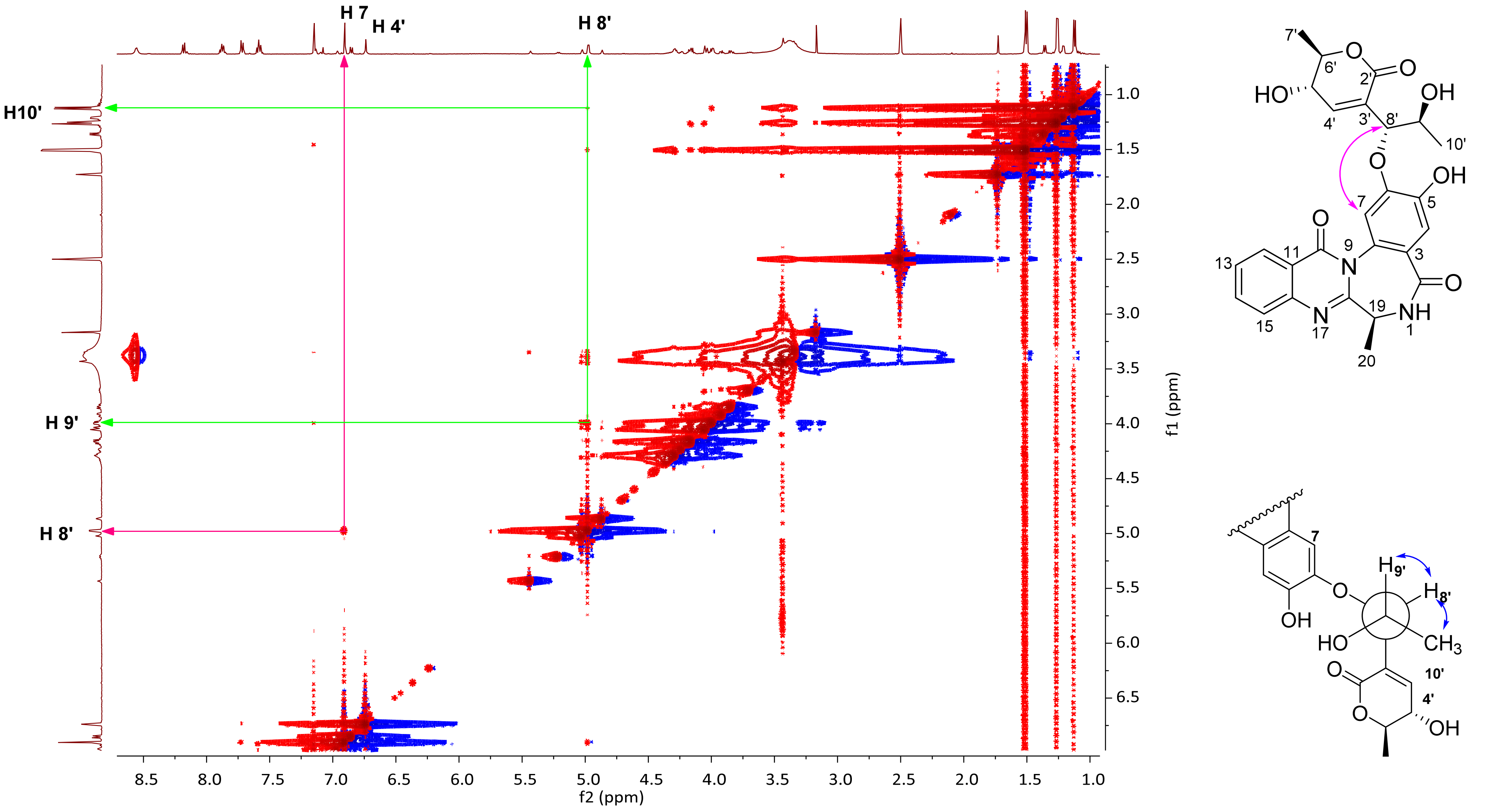

Figure S62. NOESY spectrum of ochrazepine D (**4**) in DMSO-d_6 at 25°C (2)

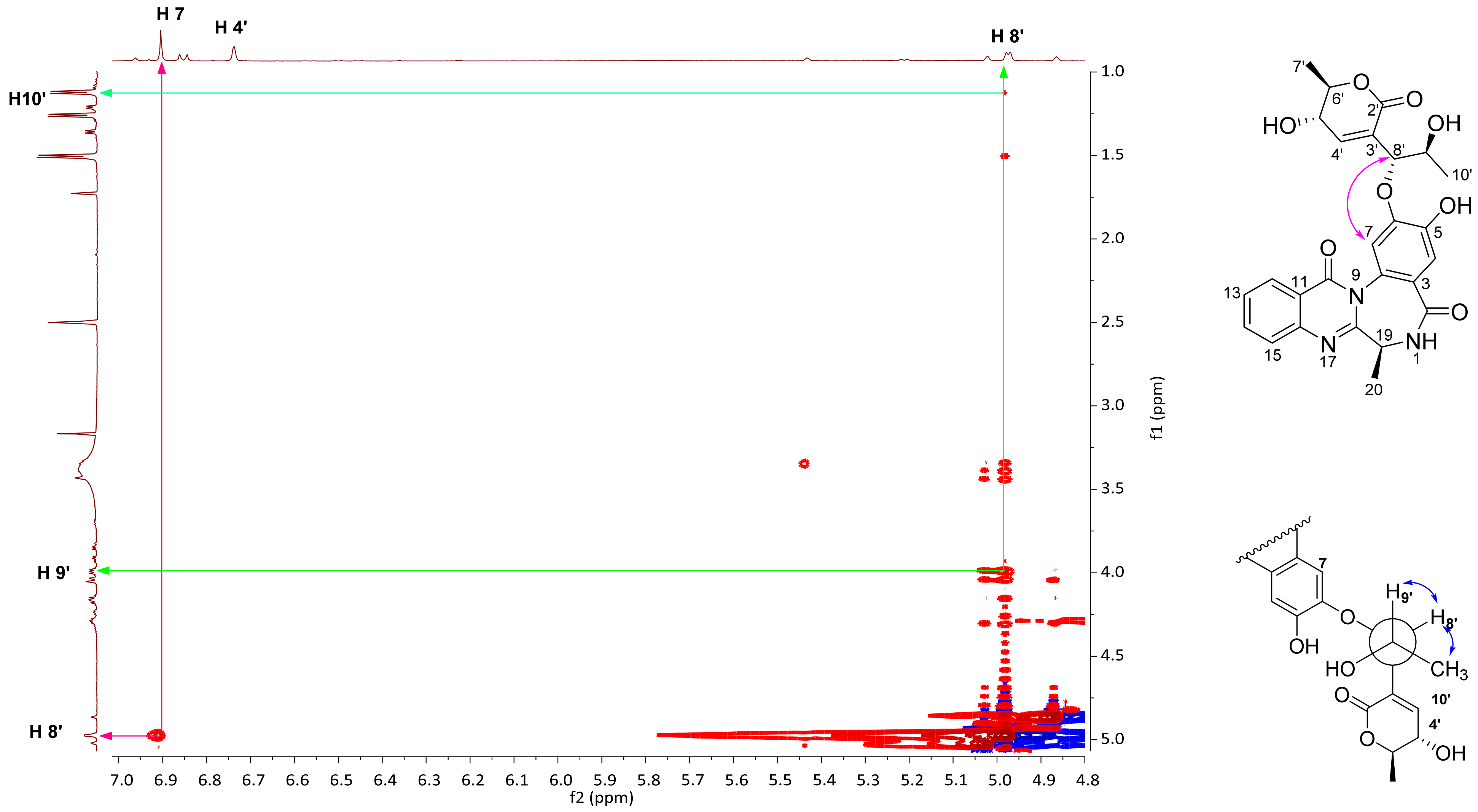

Figure S63. ^{1}H-NMR spectrum of ochrazepine D (**4**) in MeOH-d_4 at 1°C

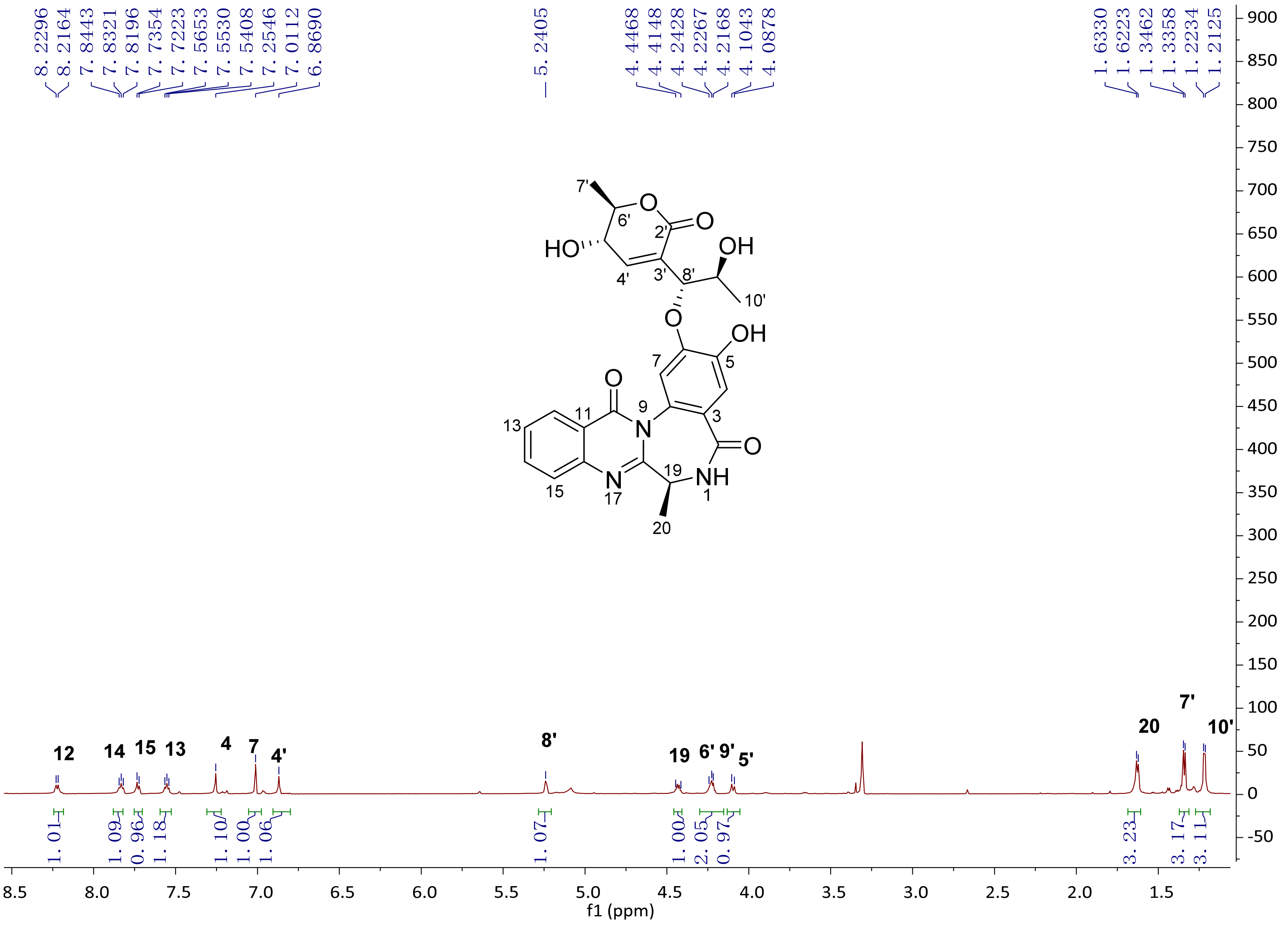

Figure S64. NOESY spectrum of ochrazepine D (**4**) in MeOH-d_4 at 1°C (1)

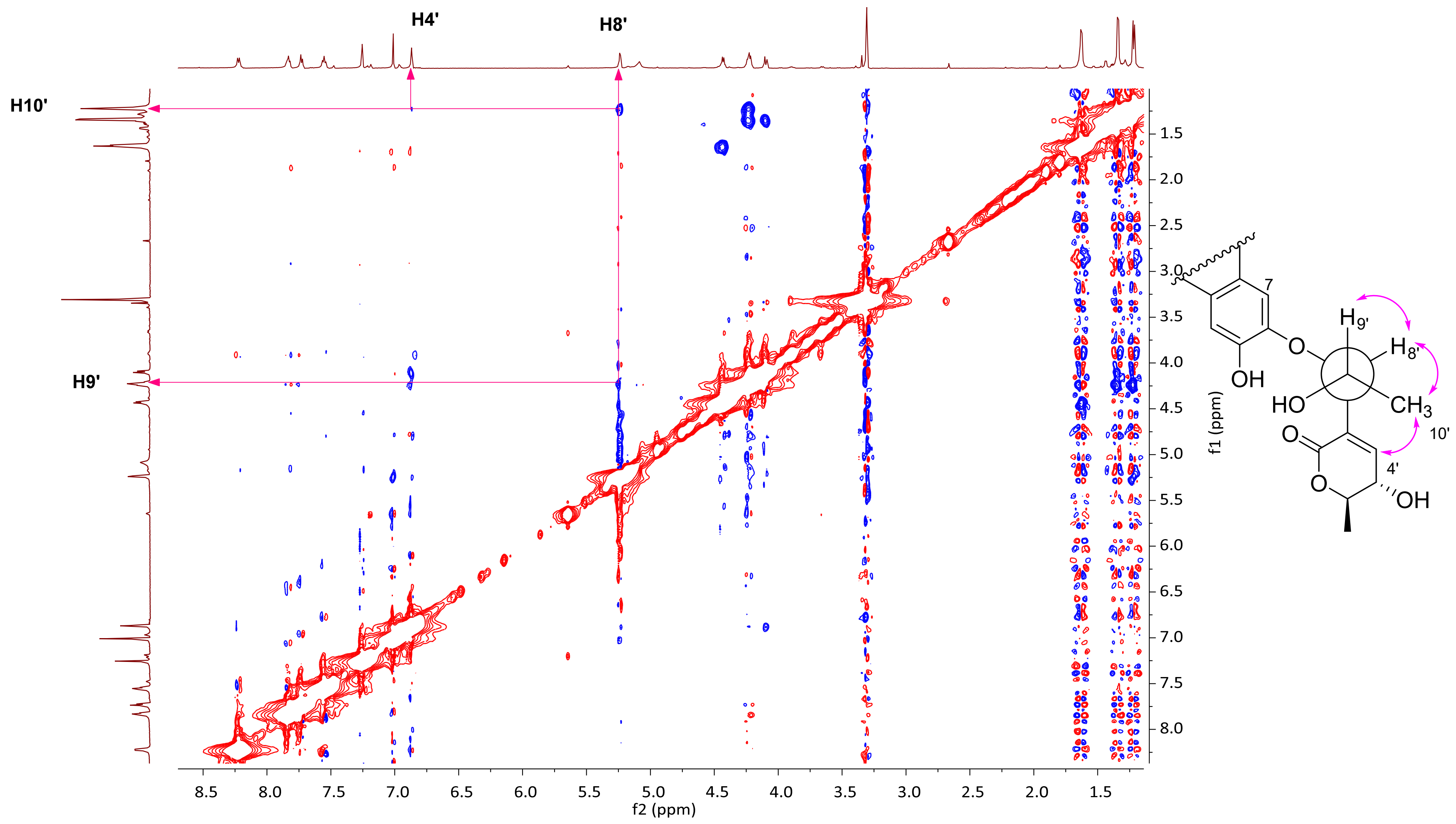

Figure S65. NOESY spectrum of ochrazepine D (**4**) in MeOH-d_4 at 1°C (2)

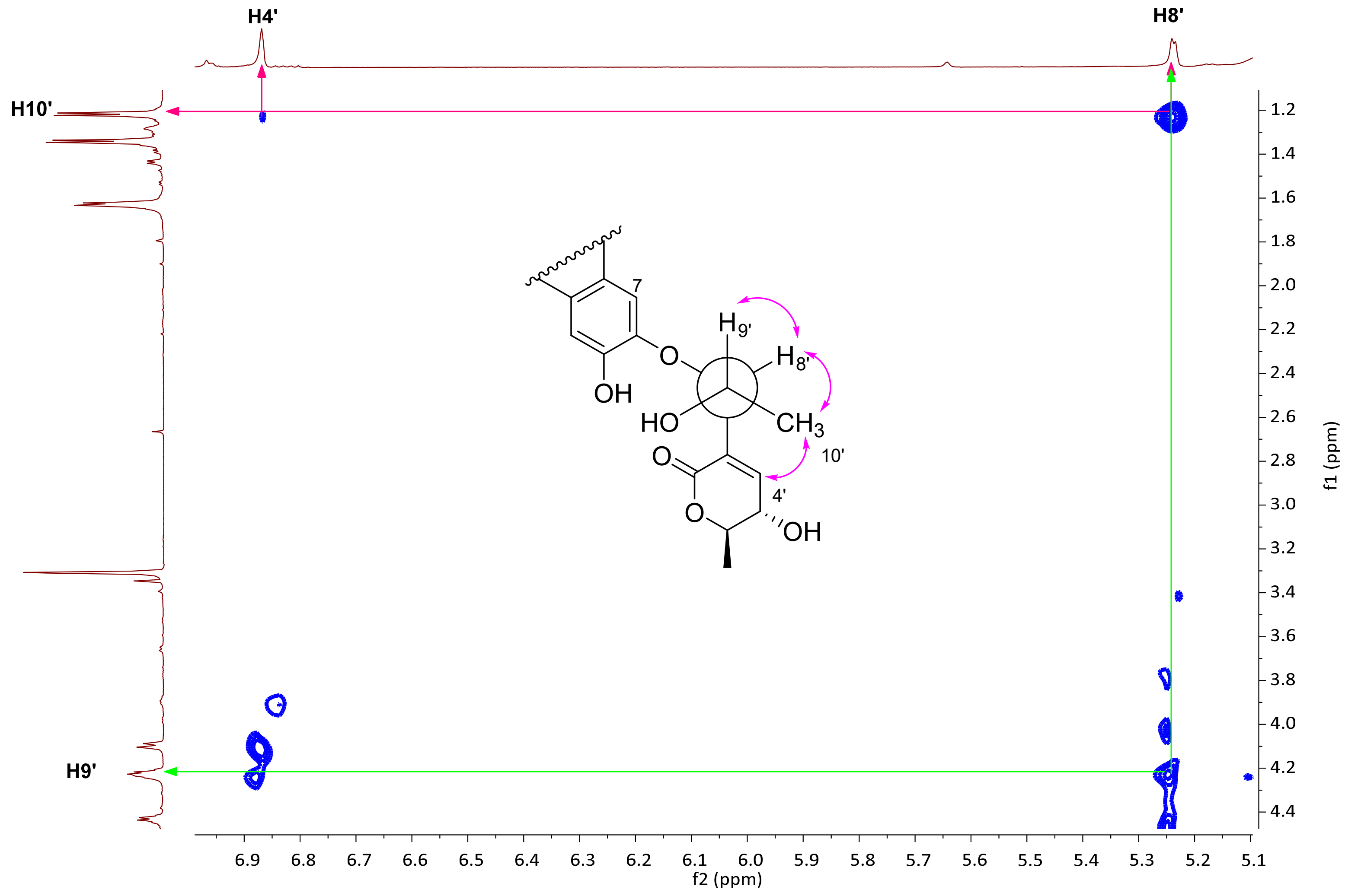

Figure S66. The determination of the absolute configuration of **5** by Marfey's method (Solvents: A water + 0.1% FA, B CH_3CN + 0.1% FA; linear gradient: 0 min, 10% B; 5 min, 99% B; 6 min, 99% B; temperature, 30 °C; column: ACQUITY UPLC BEH C_{18}, 2.1 × 50 mm, 1.7 μm; flow rate, 0.4 mL/min; UV detection at λ 340 nm; FDAA, 2.86 min)

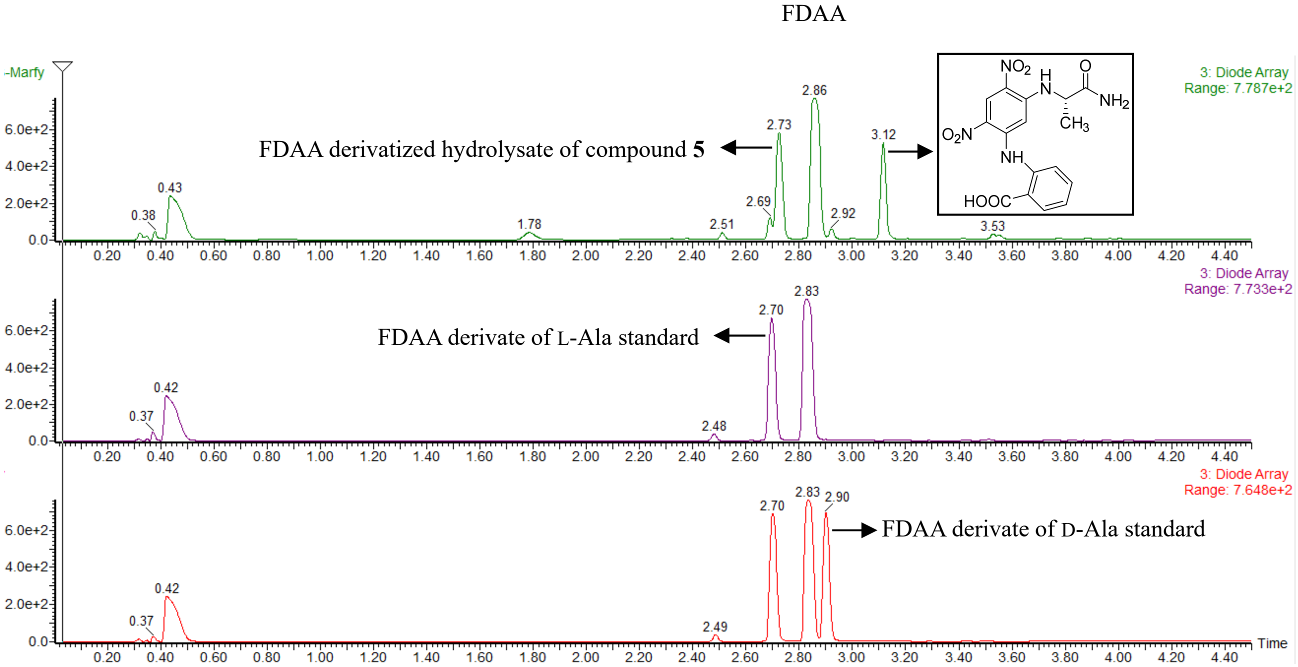

Figure S67. ECD curves of compounds **1**–**4**

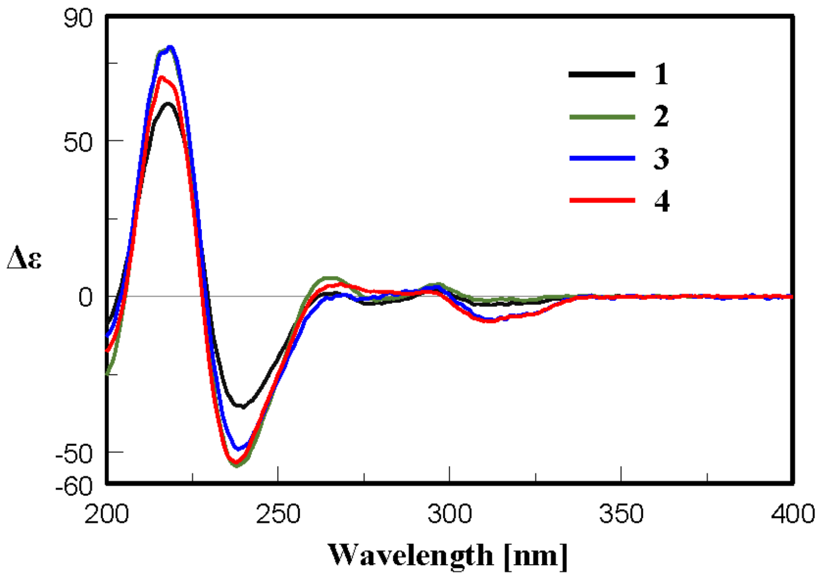

Supplement: Supplementary file 1 [file marinedrugs-17-00400-s001.pdf]
